# Synthesis and Biological Evaluation of Curcumin Derivatives with Water-Soluble Groups as Potential Antitumor Agents: An *in Vitro* Investigation Using Tumor Cell Lines

**DOI:** 10.3390/molecules201219772

**Published:** 2015-12-02

**Authors:** Luyang Ding, Shuli Ma, Hongxiang Lou, Longru Sun, Mei Ji

**Affiliations:** Department of Natural Products Chemistry, Key Lab of Chemical Biology (MOE), School of Pharmaceutical Sciences, Shandong University, No. 44 West Wenhua Road, Jinan 250012, China; sdu2013dly@sina.cn (L.D.); MASHULI789@126.com (S.M.); louhongxiang@sdu.edu.cn (H.L.); jimei@sdu.edu.cn (M.J.)

**Keywords:** curcumin, curcumin derivatives, synthesis, antitumor cell line growth activity, apoptosis

## Abstract

Three series of curcumin derivatives including phosphorylated, etherified, and esterified products of curcumin were synthesized, and their anti-tumor activities were assessed against human breast cancer MCF-7, hepatocellular carcinoma Hep-G2, and human cervical carcinoma HeLa cells. Compared with curcumin, compounds **3**, **8**, and **9** exhibited stronger antitumor cell line growth activities against HeLa cells. Compound **12** also showed higher antitumor cell line growth activities on MCF-7 cells than curcumin. Among them, 4-((1*E*,6*E*)-7-(4-Hydroxy-3-methoxyphenyl)-3,5-dioxohepta-1,6-dienyl)-2-methoxyphenyl dihydrogen phosphate(**3**) showed the strongest activity with an half maximal inhibitory concentration (IC_50_) of 6.78 µM against HeLa cells compared with curcumin with an IC_50_ of 17.67 µM. Stabilities of representatives of the three series were tested in rabbit plasma *in vitro*, and compounds **3** and **4** slowly released curcumin in plasma. The effect of compound **3** on HeLa cell apoptosis was determined by examining morphological changes by DAPI (4′,6-diamidino-2-phenylindole) staining as well as Annexin V-FITC/ Propidium Iodide (PI) double staining and flow cytometry. The results showed that **3** induced cellular apoptosis in a dose-dependent manner. Together our findings show that **3** merits further investigation as a new potential antitumor drug candidate.

## 1. Introduction

Curcumin, a phenolic compound isolated from the rhizome of *Curcuma longa* L. (*Zingiberaceae*), has been widely used as a food colorant and spicein China, India, and Southeast Asia [1]. Many investigations have shown that curcumin and its derivatives have several biological activities. The main pharmacological effects include anti-tumor [2,3], anti-inflammatory [4,5], anti-oxidation [6], anti-fungal [7], and anti-bacterial activities [8]. Accumulating evidence also suggests that curcumin modulates multiple signal transduction pathways, such as NF-κB and STAT3 signaling [9]. Importantly, curcumin is safe and exhibits non-toxicity even at high doses as shown by a dose escalation from 500 to 12,000 mg [10]. However, a phase I human clinical trial demonstrated that curcumin shows difficulty in reaching the blood circulatory system and target tissues by oral administration with a low oral bioavailability [11]. Another study showed that 500 mg/kg of curcumin given in rats gave a maximum serum curcumin level of 0.06 ± 0.01 µg/mL, with an elimination half-life of 28.1 ± 5.6 min oral bioavailability of approximately 1% [12].

To improve the drug effect of curcumin, numerous approaches have been undertaken. Some adjuvants like piperine were used to interfere with glucuronidation to extend the half-life of curcumin [13]. Lipidic formulation of curcumin can optimize oral delivery of curcumin by forming nanosized globules upon dilution with aqueous medium [14]. In recent years, several curcumin analogs or derivatives have been synthesized and a few have shown stronger anticancer activity than that of curcumin [15,16,17,18,19,20]. To analyze structure–activity relationships, different curcumin-related compounds were synthesized and systematically tested for their anticancer activities and anti-oxidation properties, and some are more effective in inhibiting the growth of certain cancer cells compared with curcumin [15,16,17]. Several monocarbonyl curcumin analogs also exhibit good anti-tumor effects [18]. In particular, some studies were focused on basic nitrogen heteroaromatics to improve both solubility in aqueous media and enhance potential ability to cross cellular membranes [19,20].

To improve the solubility of curcumin in aqueous medium, here we synthesized three series of curcumin derivatives, including phosphorylated, etherified, and esterified products of curcumin. All the derivatives were evaluated for their anti-tumor effects and stabilities in rabbit plasma *in vitro*.

## 2. Results and Discussion

### 2.1. Synthetic Chemistry

**Scheme 1:**
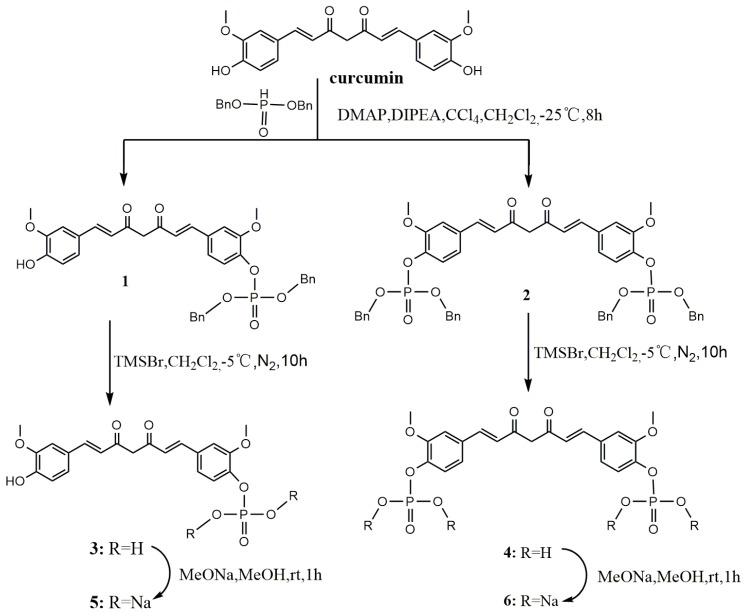
Synthesis of curcumin phosphorylated derivatives.

The first series included phosphorylated curcumin compounds. Phosphates and sodium phosphate salts of curcumin were synthesized as shown in Scheme 1. Curcumin was treated with dibenzyl phosphate in anhydrous ethyl acetate at −25 °C under a N_2_ atmosphere to obtain compounds **1** and **2**. Trimethylbromosilane (TMSBr) treatment is a debenzylation of **1** or **2** in anhydrous dichloromethane at −5 °C under a N_2_ atmosphere resulted in the formation of compound **3** or **4**, respectively. Compounds **5** and **6** were obtained by a reaction of **3** and **4** with MeONa in MeOH solution, respectively, for 1 h. The solubility of compounds **3**, **4**, **5**, or **6** in water was greatly increased.

**Scheme 2:**
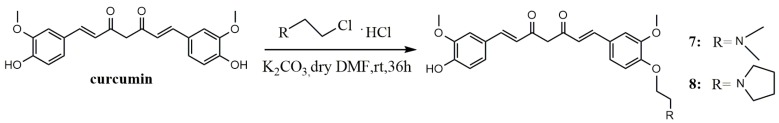
Synthesis of curcumin etherified derivatives.

The synthetic route to the curcumin etherification derivatives is depicted in Scheme 2. With the introduction of nitrogen polar groups, compounds **7** and **8** have greatly improved solubility in water and stability in plasma.

**Scheme 3:**
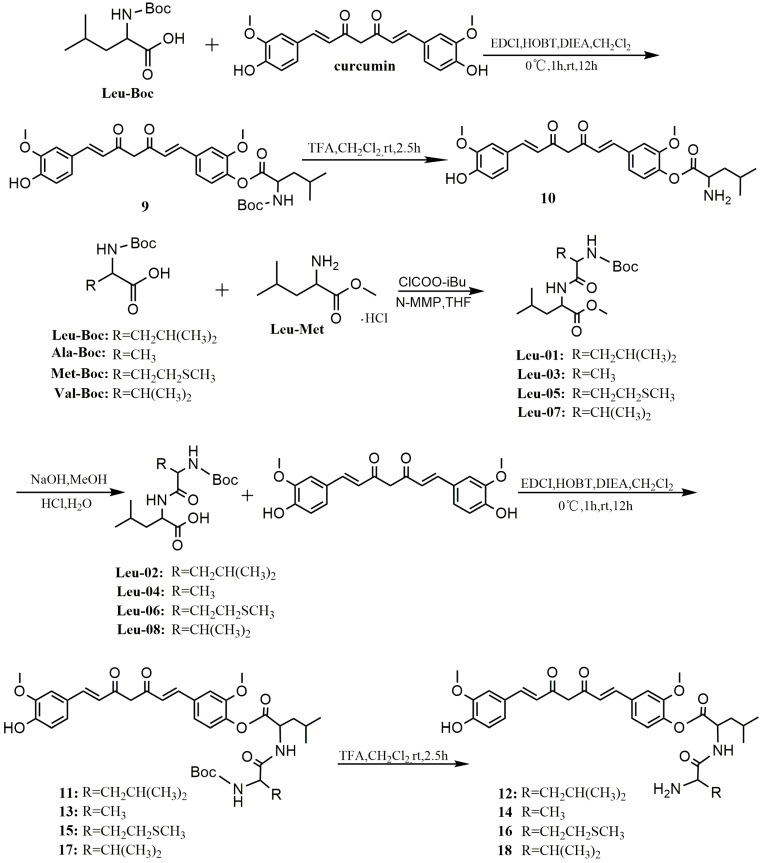
Synthesis of curcumin esterified derivatives.

The last series of derivatives of curcumin, compounds **10**, **12**, **14**, **16**, and **18**, were synthesized by condensation between carboxylic group of amino acid and phenolic group of curcumin as shown in Scheme 3. First, compounds **Leu-01**, **-03**, **-05**, and **-07**, as main raw materials, were prepared using l-leucine methyl ester hydrochloride through amidation reactions with different amino acids in tetrahydrofuran (THF), and then translated into compounds **Leu-02**, **-04**, **-06**, or **-08**, respectively, by basic hydrolysis. **Leu-Boc**, **Leu-02**, **-04**, **-06**, and **-08** were reacted with curcumin in the presence of EDCI/HOBT to give **9**, **11**, **13**, **15**, and **17**, respectively. The final products **10**, **12**, **14**, **16**, and **18** were obtained through removing the protecting tert-butyloxycarbonyl (Boc) group from **9**, **11**, **13**, **15**, and **17**, respectively, in anhydrous dichloromethane with trifluoroacetic acid (TFA).

The general process for synthesis, synthetic yields, ^1^H-NMR, ^13^C-NMR, ESI-MS, and HRESI-MS analysis of these compounds are described in the following experimental section. The spectra are shown in Supplementary Materials.

### 2.2. Stability of Derivatives in Plasma in Vitro

**Figure 1:**
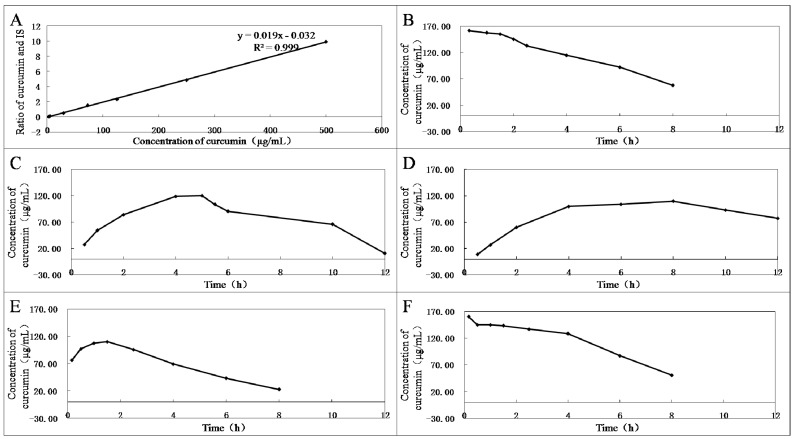
(**A**) Standard curve of the ratio of curcumin standard (2.9, 5, 29, 72.5, 125, 250, and 500 μg/mL) to dexamethasone acetate (25.5 μg/mL, IS). The mixtures of 90 µL rabbit plasma and 10 µL curcumin (**B**); and compounds **3** (**C**); **4** (**D**); **10** (**E**); or **12** (**F**) (1.1 mmol/L) were vortexed for 1 min and incubated for different times at 37 °C.

Several representative compounds **3**, **4**, **8**, **10**, **12**, and curcumin were selected to test their stabilities in plasma *in vitro* (Figure 1). First, the standard curve of the ratio of curcumin standard to dexamethasone acetate (internal standard, IS) in blank rabbit plasma was established (Figure 1A). Then, by incubation with the same plasma *in vitro* at 37 °C for different times, the hydrolysate curcumin of compounds **3**, **4**, **8**, **10**, **12**, and curcumin were measured using high-performance liquid chromatography (HPLC) method. Curcumin showed instability, and plasma concentrations were reduced over time (Figure 1B). Among the derivatives, compound **8** had the best stability and almost no curcumin could be detected in plasma within 12 h. In contrast, **12** was completely decomposed as soon as it was added into plasma (Figure 1F). Compound **10** was almost decomposed into curcumin in plasma within 1 h (Figure 1E). Compounds **3** and **4** could slowly release curcumin in plasma, and the curcumin content reached peak levels in the fifth and the eighth hours (Figure 1C,D), respectively. Therefore, compounds **3** and **4** are beneficial in maintaining curcumin in the blood for longer periods of time, which will help increase their antitumor effects.

### 2.3. Biological Activity Evaluation

#### 2.3.1. Antitumor Cell Line Growth Activity of Curcumin Derivatives

Compounds **1**–**18** were evaluated for inhibitory activities against human breast cancer MCF-7 cells as well as hepatocellular carcinoma Hep-G2 and HeLa cells using MTT (3-(4,5-dimethylthiazol-2-yl)-2,5-diphenyltetrazolium bromide) assays (Table 1). The results showed that compounds **3**, **8**, and **9** displayed better anticancer cell line growth activities against HeLa cells than other compounds, and compound **3** showed best activity (Table 1 and Supplementary Material Figures S1–S5). With regard to MCF-7 cells, **12** had a better antitumor cell line growth effect than other compounds (Table 1 and Supplementary Material Figure S6). The antitumor cell line growth activities of all derivatives against Hep-G2 cells were similar to or lower than that of curcumin.

**Table 1:** Antitumor cell line growth activities of compounds against three tumor cell lines *in vitro.* Data are expressed as mean ± SD (*n* = 3).

Compound	IC_50_ (μM)		
	MCF-7	HeLa	Hep-G2
Curcumin	9.40 ± 0.49	17.67 ± 1.10	22.88 ± 1.26
**1**	21.91 ± 1.73	23.32 ± 3.95	40.31 ± 1.10
**2**	53.46 ± 4.73	>80	>80
**3**	13.80 ± 0.88	6.78 ± 0.19	37.80 ± 0.71
**4**	45.74 ± 3.32	19.47 ± 1.54	81.71 ± 0.69
**5**	26.94 ± 1.20	15.60 ± 2.33	42.39 ± 5.58
**6**	>80	67.39 ± 5.59	>80
**7**	28.15 ± 1.37	13.66 ± 0.56	56.37 ± 2.18
**8**	12.90 ± 0.53	8.43 ± 0.32	17.37 ± 0.68
**9**	17.52 ± 2.60	9.57 ± 0.22	22.54 ± 1.99
**10**	39.22 ± 4.90	29.81 ± 1.46	48.82 ± 3.30
**11**	8.60 ± 0.20	18.14 ± 0.77	25.66 ± 1.97
**12**	6.64 ± 0.46	15.61 ± 0.65	19.70 ± 0.69
**13**	8.99 ± 0.49	11.52 ± 0.58	19.04 ± 1.22
**14**	9.35 ± 0.76	20.80 ± 1.74	31.83 ± 1.22
**15**	8.71 ± 0.86	13.72 ± 0.89	22.36 ± 0.99
**16**	9.22 ± 0.14	16.41 ± 0.09	29.07 ± 0.96
**17**	9.21 ± 0.56	10.56 ± 0.31	22.37 ± 1.92
**18**	9.61 ± 1.03	14.28 ± 1.17	28.47 ± 1.37

#### 2.3.2. Morphological Analysis with DAPI

**Figure 2:**
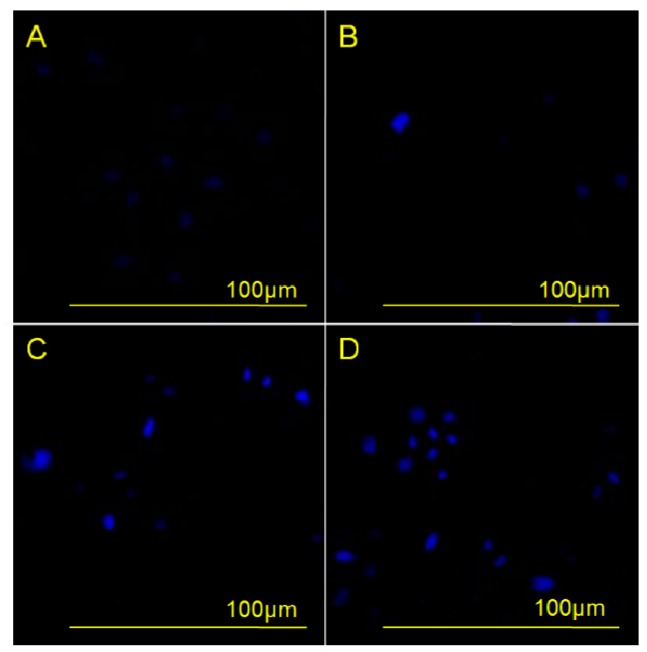
Effects of compound **3** on human cervical carcinoma HeLa cell apoptosis were observed by DAPI staining under an inversion fluorescent microscope. HeLa cells were incubated with 0 µM (**A**); 2.5 µM (**B**); 5 µM (**C**); or 10 µM (**D**) of compound **3** for 48 h.

The morphological change of nuclei during apoptosis was evaluated by DAPI (4′,6-diamidino-2-phenylindole) staining of HeLa cells incubated with different concentrations of compound **3** for 48 h. The results showed that apoptotic cell nuclei presented obvious nuclear shrinkage, and even some nuclei had fragments, as shown in Figure 2. Furthermore, as the concentration of **3** increased, the frequency of shrunk nuclei and fragments also increased, indicating that apoptosis of HeLa cells increased along with increasing concentration of **3**. These results demonstrated a concentration-dependent effect of **3** on the induction of apoptosis.

#### 2.3.3. Cell Apoptosis via Annexin V-FITC/ Propidium Iodide (PI) Double Staining Assay

**Figure 3:**
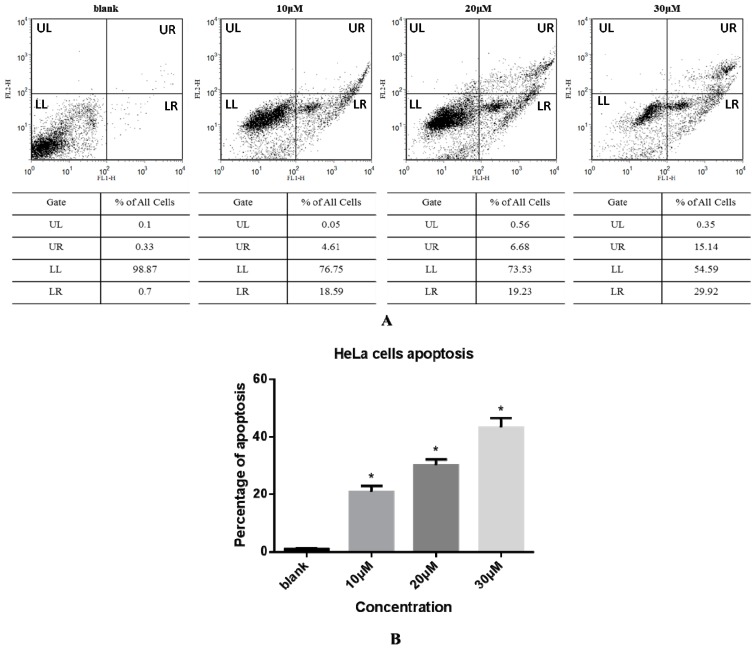
(**A**) HeLa cells were treated with compound **3** (0, 10, 20, or 30 µM) for 24 h and then analyzed by Annexin V-FITC/PI staining and flow cytometry. The apoptotic cell scatter plot is divided into four quadrants: upper left (UL), upper right (UR), lower left (LL), and lower right (LR) quadrants; (**B**) Apoptosis rate of HeLa cells treated with various concentrations of compound **3**. Data represent mean ± SD (*n* = 3). * Significantly different from respective control, *p* < 0.05.

The effect of compound **3** on apoptosis of HeLa cells was next evaluated by an Annexin V-FITC and PI double staining method and flow cytometry as shown in Figure 3. The apoptotic cell scatter plot is divided into four quadrants, including the upper left (UL), upper right (UR), lower left (LL), and lower right (LR) quadrants, which represent damaged cells, late apoptotic cells, living cells, and early apoptotic cells, respectively. The total cells in the UR and LR quadrants were regarded as apoptotic cells (Figure 3A). The total percentages of apoptotic cells upon treatment with 0, 10, 20, and 30 µM compound **3** were 1.03%, 23.20%, 25.91%, and 45.06%, respectively (Figure 3B). These data show that the rate of apoptosis increased sharply with increasing concentration of **3** in a dose-dependent manner.

## 3. Experimental Section

### 3.1. Chemistry

#### 3.1.1. Synthesis of curcumin phosphorylated derivatives.

*Dibenzyl 4-((1E,6E)-7-(4-hydroxy-3-methoxyphenyl)-3,5-dioxohepta-1,6-dienyl)-2-methoxyphenyl phosphate* (**1**). To a solution of curcumin (0.3 g, 0.81 mmol), CCl_4_ (0.78 mL, 8.10 mmol), DIPEA (0.44 g, 3.42 mmol) and DMAP (19.90 mg, 0.16 mmol) in anhydrous ethyl acetate (30 mL), dibenzyl phosphate (0.641 g, 2.44 mmol) was added dropwise at −25 °C under a N_2_ atmosphere. The resulting homogeneous mixture was stirred for 8 h at −25 °C and evaporated to dryness under reduced pressure. The residue was diluted to 100 mL with ethyl acetate, successively washed with water (3 × 100 mL) and brine (3 × 100 mL), and dried over anhydrous Na_2_SO_4_. After filtration, the filtrate was evaporated under reduced pressure to give crude product, which was purified by silica gel chromatography to give yellow oil (0.1 g, 20%). ^1^H-NMR (600 MHz, DMSO-*d*_6_) δ: 9.74 (s, 1H), 7.60 (d, *J =* 6.2 Hz, 1H), 7.58 (d, *J =* 6.2 Hz, 1H), 7.50 (s, 1H), 7.46–7.35 (m, 10H), 7.34 (d, *J =* 5.2 Hz, 1H), 7.28 (d, *J =* 8.3 Hz, 1H), 7.23 (d, *J =* 8.3 Hz, 1H), 7.17 (d, *J =* 8.1 Hz, 1H), 6.95 (d, *J =* 15.9 Hz, 1H), 6.83 (d, *J =* 8.1 Hz, 1H), 6.80 (d, *J =* 15.8 Hz, 1H), 6.12 (d, *J =* 8.6 Hz, 1H), 5.19 (d, *J =* 8.1 Hz, 4H), 3.85 (d, *J =* 10.4 Hz, 6H);^13^C-NMR (150 MHz, DMSO-*d*_6_) δ: 185.3, 181.8, 151.1, 149.9, 148.4, 142.0, 140.8, 139.3, 136.2, 133.4, 128.9 (5C), 128.7, 128.3 (5C), 127.9, 126.6, 124.9, 123.8, 121.8, 121.6, 116.1, 112.8, 111.8, 101.8, 69.8 (2C), 56.5, 56.1; ESI-MS [M − H]^−^ *m/z*: 627.5.

*Dibenzyl 4,4′-((1E,6E)-3,5-dioxohepta-1,6-diene-1,7-diyl)bis(2-methoxy-4,1-phenylene) diphosphate* (**2**). The synthetic method of **2** was similar to that of compound **1**, affording yellow oil (0.28 g, 39%). ^1^H-NMR (600 MHz, DMSO-*d*_6_) δ: 7.64 (d, *J =* 15.8 Hz, 2H), 7.52 (s, 2H), 7.38 (s, 20H), 7.30 (d, *J =* 8.4 Hz, 2H), 7.24 (d, *J =* 8.2 Hz, 2H), 6.99 (d, *J =* 15.9 Hz, 2H), 6.20 (s, 1H), 5.19 (d, *J =* 8.0 Hz, 8H), 3.85 (d, *J =* 10.3 Hz, 6H); ^13^C-NMR (150 MHz, DMSO-*d*_6_) δ: 183.6 (2C), 151.1 (2C), 141.0 (2C), 140.1 (2C), 136.1 (4C), 133.3 (2C), 128.9 (12C), 128.3 (10C), 124.9 (2C), 121.8 (2C), 112.9 (2C), 102.1, 69.8 (4C), 56.4 (2C); ESI-MS [M − H]^−^ *m/z*: 887.6.

*4-((1E,6E)-7-(4-Hydroxy-3-methoxyphenyl)-3,5-dioxohepta-1,6-dienyl)-2-methoxyphenyl dihydrogen phosphate* (**3**). TMSBr (0.13 mL, 0.982 mmol) was added dropwise to a stirred solution of **1** (0.308 g, 0.491 mmol) in anhydrous dichloromethane (5 mL) at −5 °C under a N_2_ atmosphere. The reaction mixture was stirred for 10 h at 0 °C. The mixture was poured into methanol (20 mL) and then evaporated to dryness under reduced pressure. The residue was purified by Sephadex LH-20 column chromatography to afford yellow solid (0.153 g, 70%). m.p. 144–146 °C. ^1^H-NMR (600 MHz, MeOD-*d*_4_) δ: 7.57 (d, *J =* 9.4 Hz, 1H), 7.54 (d, *J =* 9.4 Hz, 1H), 7.38 (d, *J =* 8.2 Hz, 1H), 7.24 (s, 1H), 7.18 (d, *J =* 1.0 Hz, 1H), 7.13 (d, *J =* 7.8 Hz, 1H), 7.08 (d, *J =* 8.2 Hz, 1H), 6.81 (d, *J =* 8.1 Hz, 1H), 6.68 (d, *J =* 15.8 Hz, 1H), 6.61 (d, *J =* 15.8 Hz, 1H), 3.89 (d, *J =* 7.4 Hz, 6H); ^13^C-NMR (150 MHz, MeOD-*d*_4_) δ: 184.5, 181.9, 151.3, 149.1, 147.9, 143.2, 141.2, 139.5, 131.5, 129.8, 127.0, 122.9, 122.8, 121.0, 120.9, 115.1, 111.4, 110.3, 55.1, 55.0; ^31^P-NMR (243 MHz, MeOD-*d*_4_) δ:−4.31 (1P); ESI-MS [M − H]^−^ *m/z*: 447.4 ; HR-ESI-MS [M − H]^−^ *m/z*: 447.0854, Calcd. for C_21_H_21_O_9_P (M − H) 447.0923.

*4,4′-((1E,6E)-3,5-Dioxohepta-1,6-diene-1,7-diyl)bis(2-methoxy-4,1-phenylene) bis(dihydrogen phosphate)* (**4**). TMSBr (0.5 mL, 3.7 mmol) was added dropwise to a stirred solution of **2** (0.736 g, 0.83 mmol) in anhydrous dichloromethane (5 mL) at −5 °C under a N_2_ atmosphere. The reaction mixture was stirred for 10 h at 0 °C. The mixture was poured into methanol (20 mL) and evaporated to dryness under reduced pressure. The residue was purified by Sephadex LH-20 column chromatography to afford yellow solid (0.32 g, 72%). m.p. 163–166 °C; ^1^H-NMR (600 MHz, MeOD-*d*_4_) δ: 7.54 (d, *J =* 15.9 Hz, 2H), 7.27 (d, *J =* 8.3 Hz, 2H), 7.24 (s, 2H), 7.12 (dd, *J =* 8.3, 1.7 Hz, 2H), 6.69 (d, *J =* 15.9 Hz, 2H), 3.83 (s, 6H); ^13^C-NMR (150 MHz, MeOD-*d*_4_) δ: 183.2 (2C), 151.4 (2C), 142.6 (2C), 139.9 (2C), 132.1 (2C), 123.3 (2C), 121.1 (2C), 120.9 (2C), 111.5 (2C), 55.1 (2C); ^31^P-NMR (243 MHz, MeOD-*d*_4_) δ: −4.96 (2P); ESI-MS [M − 2H/2]^−^ *m/z*: 263; HR-ESI-MS [M − H]^−^ *m/z*: 527.0203, Calcd. for C_21_H_22_O_12_P_2_ (M − H) 527.0586.

*Sodium 4-((1E,6E)-7-(4-hydroxy-3-methoxyphenyl)-3,5-dioxohepta-1,6-dienyl)-2-methoxyphenyl phosphate* (**5**). Compound **3** (0.049 g, 0.11 mmol) was dissolved in anhydrous methanol (5 mL), and was added to a solution of MeONa (0.012 g, 0.22 mmol) in methanol (1 mL) with stirring for 1 h at room temperature. The mixture was evaporated to dryness and then dissolved in water (1 mL) and acetonitrile (6 mL) solution. The product was crystallized and collected by filtration to give pure dark yellow solid (0.049 g, 91%). ^1^H-NMR (600 MHz, MeOD-*d*_4_) δ: 7.57 (d, *J =* 9.4 Hz, 1H), 7.54 (d, *J =* 9.4 Hz, 1H), 7.38 (d, *J =* 8.2 Hz, 1H), 7.24 (s, 1H), 7.18 (d, *J =* 1.0 Hz, 1H), 7.13 (d, *J =* 7.8 Hz, 1H), 7.08 (d, *J =* 8.2 Hz, 1H), 6.81 (d, *J =* 8.1 Hz, 1H), 6.68 (d, *J =* 15.8 Hz, 1H), 6.61 (d, *J*= 15.8 Hz, 1H), 3.89 (d, *J =* 7.4 Hz, 6H); ^13^C-NMR (150 MHz, MeOD-*d*_4_) δ: 184.5, 181.9, 151.3, 149.1, 147.9, 143.2, 141.2, 139.5, 131.5, 129.8, 127.0, 122.9, 122.8, 121.0, 120.9, 115.1, 111.4, 110.3, 55.1, 55.0; ^31^P-NMR (243 MHz, MeOD-*d*_4_) δ: −4.31 (1P).

*Sodium 4,4′-((1E,6E)-3,5-dioxohepta-1,6-diene-1,7-diyl)bis(2-methoxy-4,1-phenylene) diphosphate* (**6**). The synthetic method of **6** was similar to that of compound **5**, giving dark yellow solid (0.063 g, 91%). ^1^H-NMR (600 MHz, MeOD-*d*_4_) δ: 7.54 (d, *J =* 15.9 Hz, 2H), 7.27 (d, *J =* 8.3 Hz,2H), 7.24 (s, 2H), 7.12 (dd, *J =* 8.3, 1.7 Hz, 2H), 6.69 (d, *J =* 15.9 Hz, 2H), 3.83 (s, 6H); ^13^C-NMR (150 MHz, MeOD-*d*_4_) δ: 183.2 (2C), 151.4 (2C), 142.6 (2C), 139.9 (2C), 132.1 (2C), 123.3 (2C), 121.1 (2C), 120.9 (2C), 111.5 (2C), 55.1 (2C); ^31^P-NMR (243 MHz, MeOD-*d*_4_) δ: −4.96 (2P).

#### 3.1.2. General Procedure for Synthesis of Curcumin Etherified Derivatives 

A mixture of curcumin (0.5 g, 1.36 mmol), *N*,*N*-Dimethyl-2-chloroethylamine hydrochloride (0.196 g, 1.36 mmol) or 1-(2-ethyl chloride) pyrrolidine hydrochloride (0.23 g, 1.36 mmol) and anhydrous potassium carbonate (0.376 g, 2.72 mmol) was dissolved in anhydrous dimethyl formamide (DMF) (10 mL), stirred for 36 h at room temperature, and evaporated to dryness under reduced pressure. The residue was diluted to 100 mL with dichloromethane, successively washed with water (3 × 100 mL) and brine (3 × 100 mL), and dried over anhydrous MgSO_4_. By filtration and evaporation of filtrate under reduced pressure, a crude product was obtained, then purified by thin-layer chromatography (TLC) and Sephadex LH-20 column chromatography, respectively, to give the pure product.

*(1E,6E)-1-(4-(2-(Dimethylamino)ethoxy)-3-methoxyphenyl)-7-(4-hydroxy-3-methoxyphenyl)hepta-1,6-diene-3,5-dione* (**7**). Dark red solid; yield: 18.3%. m.p. 129–132 °C; ^1^H-NMR (400 MHz, DMSO-*d*_6_) δ: 7.58 (d, *J =* 3.8 Hz, 1H), 7.54 (d, *J =* 3.8 Hz, 1H), 7.34 (dd, *J =* 9.0, 1.5 Hz, 2H), 7.25 (d, *J =* 8.4 Hz, 1H), 7.16 (dd, *J =* 8.2, 1.6 Hz, 1H), 7.04 (d, *J =* 8.4 Hz, 1H), 6.84 (d, *J =* 5.3 Hz, 1H), 6.81 (d, *J =* 2.1 Hz, 1H), 6.77 (d, *J =* 15.8 Hz, 1H), 6.08 (s, 1H), 4.09 (t, *J =* 5.9 Hz, 2H), 3.83 (t, *J =* 5.7 Hz, 6H), 2.64 (dd, *J =* 13.5, 7.7 Hz, 2H), 2.22 (s, 6H); ^13^C-NMR (100 MHz, DMSO-*d*_6_) δ: 184.2, 183.2, 150.7, 149.9, 149.7, 148.5, 141.4, 140.6, 128.2, 126.8, 123.7, 123.3, 122.6, 121.6, 116.2, 113.3, 111.9, 111.2, 101.4, 67.1, 58.1, 56.2 (2C), 46.1 (2C); ESI-MS [M + H]^+^ *m/z*: 440; HR-ESI-MS [M + H]^+^ *m/z*: 440.2068, Calcd. for C_25_H_29_NO_6_ (M + H) 440.2028.

*(1E,6E)-1-(4-Hydroxy-3-methoxyphenyl)-7-(3-methoxy-4-(2-(pyrrolidin-1-yl)ethoxy)phenyl)hepta-1,6-diene-3,5-dione* **(8)**. Dark red solid; yield:24%. m.p. 128–132 °C; ^1^H-NMR (600 MHz, MeOD-*d*_4_) δ: 7.51 (d, *J =* 2.8 Hz, 1H), 7.49 (d, *J =* 2.8 Hz, 1H), 7.16 (s, 1H), 7.13 (s, 1H), 7.10 (d, *J =* 8.2 Hz, 1H), 7.03 (d, *J =* 8.2 Hz, 1H), 6.90 (d, *J =* 8.3 Hz, 1H), 6.75 (d, *J =* 8.2 Hz, 1H), 6.60 (d, *J =* 15.8 Hz, 1H), 6.55 (d, *J =* 15.8 Hz, 1H), 4.11 (t, *J =* 5.6 Hz, 2H), 3.83 (d, *J =* 12.0 Hz, 6H),2.95 (t, *J =* 5.6 Hz, 2H), 2.71 (d, *J =* 5.7 Hz, 4H), 1.82–1.77 (m, 4H); ^13^C-NMR (150 MHz, MeOD-*d*_4_) δ: 183.9, 182.6, 150.1, 149.7, 149.4, 148.1, 140.9, 139.9, 128.7, 126.9, 122.9, 122.3, 121.9, 120.7, 115.2, 112.9, 110.3, 110.2, 67.1, 55.0, 54.9, 54.3, 54.2 (2C), 22.8 (2C); ESI-MS [M + H]^+^ *m/z*: 466; HR-ESI-MS [M + H]^+^ *m/z*: 466.2224, Calcd. for C_27_H_31_NO_6_ (M + H) 466.2185.

#### 3.1.3. General Procedure for Synthesis of Dipeptide Methyl Ester

To a stirred solution of *N*-tert-butoxycarbonyl-l-leucine(or alanine, methionine, valine) (9.3 mmol) and *N*-methylmorpholine (2.1 mL, 19 mmol) in THF (20 mL), isobutyl chloroformate (1.4 mL, 11 mmol) was added at −5 °C and the mixture was stirred over 30 min. l-leucine methyl ester hydrochloride (1.69 g, 9.3 mmol) was added to the mixture. The stirring was continued for 1 h at −5 °C, and then the mixture was stirred at room temperature for 5 h. The solution was concentrated with a rotary evaporator in vacuo. The residue was dissolved in EtOAc and successively washed with 5% NaHCO_3_, 10% acetic acid, and brine. The EtOAc solution was dried over anhydrous Na_2_SO_4_ and concentrated with a rotary evaporator to afford a product.

*Methyl2-(2-(tert-butoxycarbonylamino)-4-methylpentanamido)-4-methylpentanoate* (**Leu-01**). White powder; yield: 92%; ^1^H-NMR (400 MHz, DMSO-*d*_6_) δ: 8.08 (d, *J =* 7.6 Hz, 1H), 6.83 (d, *J =* 8.4 Hz, 1H), 4.34–4.21 (m, 1H), 4.07–3.89 (m, 1H), 3.60 (s, 3H), 1.59 (ddd, *J =* 15.1, 12.6, 5.0 Hz, 3H), 1.47 (ddd, *J =* 13.8, 9.3, 4.9 Hz, 1H), 1.40–1.38(m, 1H), 1.37 (s, 9H), 0.96–0.74 (m, 13H); ^13^C-NMR (100 MHz, DMSO-*d*_6_) δ: 173.4, 173.1, 155.7, 78.4, 53.0, 52.2, 50.5, 41.1, 40.2,28.62 (3C), 24.6, 24.5, 23.4, 23.3, 22.2, 21.6; ESI-MS [M + Na]^+^ *m/z*: 381.

*(2S)-Methyl2-(2-(tert-butoxycarbonylamino)propanamido)-4-methylpentanoate* (**Leu-03**). White powder; yield: 92%; ^1^H-NMR (400 MHz, DMSO-*d*_6_) δ: 8.06 (d, *J =* 7.6 Hz, 1H), 6.85 (d, *J =* 7.6 Hz, 1H), 4.37–4.20 (m, 1H), 4.14–3.85 (m, 1H), 3.61 (d, *J =* 3.5 Hz, 3H), 1.70–1.59 (m, 1H), 1.59–1.43 (m, 2H), 1.37 (s,9H), 1.20–1.12 (m, 3H), 0.89–0.79 (m, 6H); ^13^C-NMR (100 MHz, DMSO-*d*_6_) δ: 173.4 (2C), 155.5, 78.5, 52.3, 50.6, 49.9, 40.3, 28.6 (3C), 24.6, 23.2, 21.8, 18.4; ESI-MS [M + Na]^+^ *m/z*: 339.

*(2S)-Methyl2-(2-(tert-butoxycarbonylamino)-4-(methylthio)butanamido)-4-methylpentanoate* (**Leu-05**). White powder; yield: 92%; ^1^H-NMR (400 MHz, DMSO-*d*_6_) δ: 8.14 (d, *J =* 7.6 Hz, 1H), 6.91 (d, *J =* 8.1 Hz, 1H), 4.59–4.13 (m, 1H), 4.13–3.93 (m, 1H), 3.61 (m, 3H), 2.45 (t, *J =* 7.6 Hz, 2H), 2.01 (d, *J =* 20.1 Hz, 3H), 1.90–1.71 (m, 2H), 1.71–1.53 (m, 2H), 1.52–1.45 (m, 1H), 1.37 (s, 9H), 0.92–0.80 (m, 6H); ^13^C-NMR (100 MHz, DMSO-*d*_6_) δ: 173.3, 172.3, 155.7, 78.6, 53.8, 52.2, 50.6, 40.2, 32.2, 30.0, 28.6 (3C), 24.6, 23.3, 21.7, 15.1; ESI-MS [M + Na]^+^ *m/z*: 399.

*(2S)-Methyl2-(2-(tert-butoxycarbonylamino)-3-methylbutanamido)-4-methylpentanoate* (**Leu-07**). White powder; yield: 92%; ^1^H-NMR (400 MHz, DMSO-*d*_6_) δ: 12.47 (s, 1H), 7.97 (d, *J =* 7.9 Hz, 1H), 6.62 (d, *J =* 9.1 Hz, 1H), 4.24 (td, *J =* 9.8, 5.2 Hz, 1H), 3.76 (m, 1H), 1.92 (m, 1H), 1.66 (d, *J =* 5.7 Hz, 1H), 1.53 (m, 2H), 1.38 (s, 9H), 0.87 (m, 12H); ^13^C-NMR (100 MHz, DMSO-*d*_6_) δ: 173.3, 172.1, 155.8, 78.5, 60.1, 52.2, 50.5, 40.2, 30.8, 28.6 (3C), 24.5, 23.2, 21.6, 19.5, 18.6; ESI-MS [M + Na]^+^ *m/z*: 367.

#### 3.1.4. General Procedure for Synthesis of Dipeptide 

**Leu-01**, **-03**, **-05**, or **-07** (0.005 mmol) was dissolved in MeOH (100 mL), and 10 mL of 1 mol/L NaOH was added over 5 min with stirring. The reaction mixture was continuously stirred for 12 h at room temperature. After the pH value of the resulting solution was adjusted to 2–3 with 1 mol/L hydrochloric acid, a white precipitated solid was produced, collected by filtration, and dried to give the pure product.

*2-(2-(Tert-butoxycarbonylamino)-4-methylpentanamido)-4-methylpentanoic acid* (**Leu-02**). Pale yellow oil; yield: 93%; ^1^H-NMR (400 MHz, DMSO-*d*_6_) δ: 12.38 (s, 1H), 7.89 (d, *J =* 8.0 Hz, 1H), 6.82 (d, *J =* 8.5 Hz, 1H), 4.24 (m, 1H), 3.98 (m, 1H), 1.78–1.57 (m, 2H), 1.57–1.48 (m, 2H), 1.40 (m, 1H), 1.38 (s, 9H), 0.95–0.77 (m, 13H); ^13^C-NMR (100 MHz, DMSO-*d*_6_) δ: 174.4, 171.7, 155.7, 78.4, 53.2, 50.5, 43.2, 41.2, 28.6 (3C), 24.6,24.6, 23.4, 23.3,22.1, 21.8. ESI-MS [M − H]^−^ *m/z*: 343.

*(2S)-2-(2-(Tert-butoxycarbonylamino)propanamido)-4-methylpentanoic acid* (**Leu-04**). Pale yellow oil; yield: 93%; ^1^H-NMR (400 MHz, DMSO-*d*_6_) δ: 12.51 (s, 1H), 7.89 (d, *J =* 7.9 Hz, 1H), 6.86 (d, *J =* 7.7 Hz, 1H), 4.26–4.20 (m, 1H), 4.03–3.94 (m, 1H), 1.67–1.62 (m, 1H), 1.58–1.44 (m, 2H), 1.37 (s, 9H), 1.16 (d, *J =* 7.1 Hz, 3H), 0.82–0.9 (m, 6H); ^13^C-NMR (100 MHz, DMSO-*d*_6_) δ: 174.4, 173.1, 155.4, 78.5, 55.3, 50.5, 49.9, 28.6 (3C), 24.6, 23.3, 21.8, 18.5; ESI-MS [M − H]^−^ *m/z*: 301.

*(2S)-2-(2-(Tert-butoxycarbonylamino)-4-(methylthio)butanamido)-4-methylpentanoic acid* (**Leu-06**). Pale yellow oil; yield: 93%; ^1^H-NMR (400 MHz, DMSO-*d*_6_) δ: 12.55 (s, 1H), 8.00 (d, *J =* 7.7 Hz, 1H), 6.94 (d, *J =* 8.2 Hz, 1H), 4.25–4.19 (m, 1H), 4.04–3.99 (m,1H), 2.45 (t, *J =* 7.7 Hz, 1H), 2.03 (s, 3H), 1.88–1.72 (m, 2H), 1.66 (m, 1H), 1.58–1.47 (m, 2H), 1.38 (s, 9H), 0.90–0.83 (m, 6H); ^13^C-NMR (100 MHz, DMSO-*d*_6_) δ: 174.4, 172.1, 155.7, 78.6, 55.4, 53.9, 50.6, 32.2, 30.0, 28.6 (3C), 24.6, 23.4, 21.7, 15.1; ESI-MS [M − H]^−^ *m/z*: 361.

*(2S)-2-(2-(Tert-butoxycarbonylamino)-3-methylbutanamido)-4-methylpentanoic acid* (**Leu-08**). Pale yellow oil; yield: 93%; ^1^H-NMR (400 MHz, DMSO-*d*_6_) δ: 8.15 (d, *J =* 7.6 Hz, 1H), 6.60 (t, *J =* 23.2 Hz, 1H), 4.30 (m, 1H), 3.79 (m, 1H), 3.60 (s, 3H), 1.89 (dt, *J =* 26.8, 10.0 Hz, 1H), 1.66 (m, 1H), 1.56 (m, 1H), 1.48 (ddd, *J =* 13.8, 9.2, 5.0 Hz, 1H), 1.38 (s, 9H), 0.85 (m, 12H); ^13^C-NMR (100 MHz, DMSO-*d*_6_) δ: 173.3, 172.1, 155.8, 78.5, 60.1, 52.2, 50.5, 30.8, 28.6 (3C), 24.5, 23.3, 21.6, 19.6, 18.7; ESI-MS [M − H]^−^ *m/z*: 329.

#### 3.1.5. General Procedure of Curcumin Dipeptide Conjugation 

A mixture of **Leu-Boc**, **Leu-02**, **-04**, **-06**, or **-08** (1.36 mmol), EDCI (0.244 g, 1.36 mmol), HOBT (0.184 g, 1.36 mmol), and DIEA (240 µL, 1.36 mmol) in anhydrous dichloromethanewas stirred for 1 h at 0 °C. Curcumin (0.5 g, 1.36 mmol) was dissolved in anhydrous dichloromethaneand added dropwise to the above solution and was stirred overnight at room temperature. The mixture was diluted to 200 mL with dichloromethane, successively washed with 1N HCl (3 × 200 mL), water (3 × 200 mL) and brine (3 × 200 mL), and dried over anhydrous MgSO_4_. After filtration, the filtrate was evaporated under reduced pressure to give crude product, which was purified by preparative TLC and silica gel column chromatography (petroleum ether/ethyl acetate 8:1) to give the pure product.

*4-((1E,6E)-7-(4-Hydroxy-3-methoxyphenyl)-3,5-dioxohepta-1,6-dienyl)-2-methoxyphenyl 2-(tert-butoxycarbonylamino)-4-methylpentanoate* (**9**). Yellow powder; yield: 21%; m.p. 93–96 °C; ^1^H-NMR (600 MHz, MeOD-*d*_4_) δ: 7.56 (d, *J =* 7.9 Hz, 1H), 7.53 (d, *J =* 8.0 Hz, 1H), 7.26 (s, 1H), 7.16 (s, 2H), 7.06 (d, *J =* 8.2 Hz, 1H), 7.03 (d, *J =* 8.1 Hz, 1H), 6.78 (d, *J =* 8.1 Hz, 1H), 6.72 (d, *J =* 15.8 Hz, 1H), 6.60 (d, *J =* 15.8 Hz, 1H), 4.35 (dd, *J =* 10.0, 4.9 Hz, 1H), 3.84 (d, *J =* 31.8Hz, 6H), 1.84–1.78 (m, 1H), 1.77–1.71 (m, 1H), 1.71.64 (m, 1H), 1.43 (s, 9H), 0.96 (dd, *J =* 13.6, 6.5 Hz, 6H); ^13^C-NMR (150 MHz, MeOD-*d*_4_) δ: 185.1, 181.2, 171.6, 156.8, 151.5, 149.2, 147.9, 141.5, 141.1, 138.9, 134.4, 126.9, 124.0, 122.9, 122.7, 120.9, 120.7, 115.1, 111.3, 110.3, 101.1, 79.2, 55.1, 55.0, 52.2, 40.1, 27.3 (3C), 24.6, 21.9, 20.5; ESI-MS [M − H]^−^ *m*/*z*: 580.

*4-((1E,6E)-7-(4-Hydroxy-3-methoxyphenyl)-3,5-dioxohepta-1,6-dienyl)-2-methoxyphenyl 2-(2-(tert-butoxycarbonylamino)-4-methylpentanamido)-4-methylpentanoate* (**11**). Yellow powder; yield: 28%; m.p. 100–102 °C; ^1^H-NMR (400 MHz, MeOD-*d*_4_) δ: 8.43 (d, *J =* 7.8 Hz, 1H), 7.60 (d, *J =* 15.6 Hz, 2H), 7.32 (s, 1H), 7.22 (s, 2H), 7.11 (d, *J =* 8.3 Hz, 1H), 7.06 (d, *J =* 7.7 Hz, 1H), 6.82 (d, *J =* 8.1 Hz, 1H), 6.73 (d, *J =* 16.2 Hz, 1H), 6.65 (d, *J =* 16.1 Hz, 1H), 6.01 (s, 1H), 4.76–4.69 (m, 1H), 4.19–4.05 (m, 1H), 3.88 (d, *J =* 20.3 Hz, 6H), 1.90–1.79 (m, 3H), 1.77–1.66 (m, 1H), 1.63–1.48 (m, 2H), 1.43 (s, 9H), 1.05–0.89 (m, 12H); ^13^C-NMR (100 MHz, MeOD-*d*_4_) δ: 185.2, 181.2, 173.8, 170.6, 156.4, 151.5, 149.3, 148.1, 141.5, 141.1, 138.9, 134.5, 127.1, 124.2, 122.9, 122.7, 121.0, 120.7, 115.2, 111.4, 110.5, 101.0, 79.1, 55.1 (2C), 52.9, 50.8, 40.8, 40.1, 27.3 (3C), 24.5 (2C), 21.9 (4C); ESI-MS [M − H]^−^ *m*/*z*: 693.

*(2S)-4-((1E,6E)-7-(4-Hydroxy-3-methoxyphenyl)-3,5-dioxohepta-1,6-dienyl)-2-methoxyphenyl 2-(2-(tert-butoxycarbonylamino)propanamido)-4-methylpentanoate* (**13**). Yellow powder; yield: 33%; m.p. 100–102 °C; ^1^H-NMR (400 MHz, MeOD-*d*_4_) δ: 7.49 (dd, *J =* 15.8, 3.7 Hz, 2H), 7.19 (d, *J =* 20.1 Hz, 1H), 7.16 (s, 2H), 7.02–6.96 (m, 2H), 6.72 (d, *J =* 8.1 Hz, 1H), 6.67 (d, *J =* 15.7 Hz, 1H), 6.54 (d, *J =* 15.7 Hz, 1H), 4.61 (m, 1H), 4.01 (m, 1H), 3.78 (d, *J =* 20.1 Hz, 6H) , 1.85–1.62 (m, 3H), 1.34 (s, 9H), 1.23 (dd, *J =* 7.2, 3.7 Hz, 3H), 0.98–0.84 (m, 6H); ^13^C-NMR (100 MHz, MeOD-*d*_4_) δ: 185.0, 181.2, 174.8, 170.6, 156.1, 151.4, 149.2, 147.9, 141.5, 140.9, 138.9, 134.5, 127.1, 124.2, 122.9, 122.8, 121.0, 120.7, 115.3, 111.5, 110.6, 79.3, 55.1 (2C), 53.4, 50.6, 40.1, 27.4 (3C), 24.6, 22.0, 20.6, 17.2; ESI-MS [M − H]^−^ *m*/*z*: 651.

*(2S)-4-((1E,6E)-7-(4-Hydroxy-3-methoxyphenyl)-3,5-dioxohepta-1,6-dienyl)-2-methoxyphenyl 2-(2-(tert-butoxycarbonylamino)-4-(methylthio)butanamido)-4-methylpentanoate* (**15**). Yellow powder; yield: 35%; m.p. 95–97 °C; ^1^H-NMR (400 MHz, MeOD-*d*_4_) δ: 7.61 (d, *J =* 11.0 Hz, 2H), 7.32 (s, 1H), 7.22 (s, 2H), 7.11 (m, 2H), 6.84 (d, *J =* 8.0 Hz, 1H), 6.77 (d, *J =* 13.5 Hz, 1H), 6.66 (d, *J =* 15.3 Hz, 1H), 4.78–4.65 (m, 1H), 4.25 (m, 1H), 3.90 (d, *J =* 20.2 Hz, 6H), 2.57 (m, 2H), 2.07 (m, 2H), 2.01 (s, 3H), 1.94–1.79 (m, 3H), 1.46 (d, *J =* 1.8 Hz, 9H), 1.02 (dd, *J =* 15.1, 4.4 Hz, 6H); ^13^C-NMR (100 MHz, MeOD-*d*_4_) δ: 185.1, 181.3, 173.8, 170.6, 156.3, 151.5, 149.3, 148.1, 141.5, 141.0, 138.9, 134.5, 127.1, 124.2, 122.9, 122.8, 121.0, 120.7, 115.2, 111.4, 110.5, 79.3, 55.1 (2C), 53.6, 50.9, 39.9, 31.7, 29.6, 27.3 (3C), 24.6, 21.9, 20.5, 13.9; ESI-MS [M − H]^−^ *m*/*z*: 711.

*(2S)-4-((1E,6E)-7-(4-Hydroxy-3-methoxyphenyl)-3,5-dioxohepta-1,6-dienyl)-2-methoxyphenyl 2-(2-(tert-butoxycarbonylamino)-3-methylbutanamido)-4-methylpentanoate* (**17**). Yellow powder; yield: 35%; m.p. 106–109 °C; ^1^H-NMR (400 MHz, MeOD-*d*_4_) δ: 7.61 (dd, *J =* 15.8, 6.4 Hz, 2H), 7.32 (s, 1H), 7.22 (d, *J =* 6.1 Hz, 2H), 7.11 (m, 2H), 6.84 (d, *J =* 8.2 Hz, 1H), 6.78 (d, *J =* 15.9 Hz, 1H), 6.66 (d, *J =* 15.8 Hz, 1H), 6.02 (s, 1H), 4.70 (m, 1H), 3.95 (m, 1H), 3.89 (d, *J =* 22.4 Hz, 6H), 2.06 (dt, *J =* 13.6, 6.7 Hz, 1H), 1.86 (m, 3H), 1.46 (d, *J =* 2.5 Hz, 9H), 1.01 (m, 12H); ^13^C-NMR (100 MHz, MeOD-*d*_4_) δ: 185.2, 181.3, 173.4, 170.6, 156.5, 151.5, 149.3, 148.0, 141.5, 141.0, 138.9, 134.5, 127.1, 124.2, 122.9, 122.7, 121.0, 120.7, 115.2, 111.4, 110.5, 101.1, 79.1, 60.2, 55.1 (2C), 50.8, 39.9, 30.7, 27.3(3C), 24.5, 21.9, 20.4, 18.4, 17.1; ESI-MS [M − H]^−^ *m*/*z*: 679.

#### 3.1.6. General Procedure for Deprotection of Boc Group

TFA (200 µL) was added dropwise with stirring to a solution of compounds **9**, **11**, **13**, **15**, and **17** (0.136 mmol) in anhydrous dichloromethane at 0 °C. The reaction mixture was stirred for 2.5 h at room temperature. The mixture was evaporated to dryness to give the pure product.

*4-((1E,6E)-7-(4-Hydroxy-3-methoxyphenyl)-3,5-dioxohepta-1,6-dienyl)-2-methoxyphenyl 2-amino-4-methylpentanoate* (**10**). Dark red solid; yield: 89%; m.p. 114–116 °C; ^1^H-NMR (400 MHz, MeOD-*d*_4_) δ: 7.53 (d, *J =* 10.7 Hz, 2H), 7.29 (d, *J =* 13.8 Hz, 1H), 7.16 (d, *J =* 16.3 Hz, 2H), 7.11–7.00 (m, 2H), 6.74 (d, *J =* 6.5 Hz, 2H), 6.59 (s, 1H), 4.25 (t, *J =* 7.0 Hz, 1H), 3.82 (d, *J =* 4.0 Hz, 6H), 2.03–1.85 (m, 2H), 1.74 (m,1H), 1.00 (t, *J =* 6.5 Hz, 6H); ^13^C-NMR (100 MHz, MeOD-*d*_4_) δ: 185.4, 180.9, 167.8, 151.1, 149.3, 148.1, 141.7, 140.1, 138.5, 135.3, 127.0, 124.7, 122.9, 122.5, 120.9, 120.7, 115.2, 111.4, 110.5, 55.2, 55.1, 51.1, 39.6, 24.2, 21.1, 21.0; ESI-MS [M − H]^−^ *m/z*: 480; HR-ESI-MS [M − H]^−^ *m/z*: 480.2020, Calcd. for C_27_H_31_NO_7_ (M − H) 480.2101.

*4-((1E,6E)-7-(4-Hydroxy-3-methoxyphenyl)-3,5-dioxohepta-1,6-dienyl)-2-methoxyphenyl 2-(2-amino-4-methylpentanamido)-4-methylpentanoate* (**12**). Dark red solid; yield: 89%; m.p. 127–129 °C; ^1^H-NMR (400 MHz, MeOD-*d*_4_) δ: 7.52 (d, *J =* 14.1 Hz, 2H), 7.26 (s, 1H), 7.13 (s,2H), 7.06–6.96 (m, 2H), 6.74 (d, *J =* 7.7 Hz, 2H), 6.56 (d, *J =* 13.9 Hz, 1H), 4.66–4.70 (m, 1H), 3.91–3.85 (m, 1H), 3.80 (d, *J =* 16.2 Hz, 6H), 1.77–1.83 (m, 2H), 1.76 -1.65 (m, 3H), 1.66 (s, 1H), 1.00–0.86 (m, 12H); ^13^C-NMR (100 MHz, MeOD-*d*_4_) δ: 187.3, 183.2, 172.3, 171.7, 153.5, 151.3, 150.1, 143.6, 142.9, 140.8, 136.7, 129.1, 126.3, 124.9, 124.6, 123.0, 122.8, 117.3, 113.4, 112.5, 57.2 (2C), 53.5, 53.1, 42.5, 41.9, 26.6, 25.9, 23.9, 23.7, 22.7, 22.5; ESI-MS [M + H]^+^ *m*/*z*: 595; HR-ESI-MS [M + H]^+^ *m*/*z*: 595.3022, Calcd. for C_33_H_42_N_2_O_8_ (M + H) 595.2975.

*(2S)-4-((1E,6E)-7-(4-Hydroxy-3-methoxyphenyl)-3,5-dioxohepta-1,6-dienyl)-2-methoxyphenyl 2-(2-aminopropanamido)-4-methylpentanoate* (**14**). Dark red solid; yield: 89%; m.p. 126–128 °C; ^1^H-NMR (400 MHz, MeOD-*d*_4_) δ: 7.51 (d, *J* = 9.4 Hz, 2H), 7.22 (d, *J* = 24.9 Hz, 1H), 7.13 (s, 2H), 7.00 (s, 2H), 6.74 (d, *J* = 6.8 Hz, 2H), 6.57 (s, 1H), 4.68–4.64 (m, 1H), 3.95–3.88 (m, 1H), 3.80 (d, *J* = 16.4 Hz, 6H), 1.85–1.69 (m, 3H), 1.46 (dd, *J* = 7.0, 1.6 Hz, 3H), 0.99–0.88 (m, 6H); ^13^C-NMR (100 MHz, MeOD-*d*_4_) δ: 187.2, 183.2, 172.5, 171.9, 153.5, 151.3, 150.1, 143.6, 142.9, 140.8, 136.7, 129.1, 126.3, 124.9, 124.7, 123.0, 122.8, 117.3, 113.4, 112.6, 57.2 (2C), 53.1, 50.8, 42.0, 26.7, 23.8, 22.4, 18.3; ESI-MS [M + H]^+^ *m*/*z*: 553; HR-ESI-MS [M + H]^+^ *m*/*z*: 553.2558, Calcd. for C_30_H_36_N_2_O_8_ (M + H) 553.2505.

*(2S)-4-((1E,6E)-7-(4-Hydroxy-3-methoxyphenyl)-3,5-dioxohepta-1,6-dienyl)-2-methoxyphenyl 2-(2-amino-4-(methylthio)butanamido)-4-methylpentanoate* (**16**). Dark red solid; yield: 89%; m.p. 112–115 °C; ^1^H-NMR (400 MHz, MeOD-*d*_4_) δ: 7.52 (d, *J* = 13.3 Hz, 2H), 7.26 (s, 1H), 7.13 (s, 2H), 7.02 (s, 2H), 6.74 (d, *J* = 7.6 Hz, 2H), 6.56 (d, *J* = 12.5 Hz, 1H), 4.65 (m, 1H), 3.99 (m, 1H), 3.80 (d, *J* = 14.9 Hz, 6H), 2.53 (m, 2H), 2.13 (m, 2H), 2.00 (m, 3H), 1.76 (m, 3H), 0.94 (m,6 H); ^13^C-NMR (100 MHz, MeOD-*d*_4_) δ: 187.3, 183.2, 172.4, 170.6, 153.5, 151.3, 150.1, 143.6, 142.9, 140.8, 136.7, 129.1, 126.4, 124.9, 124.7, 123.0, 122.8, 117.3, 113.4, 112.6, 57.2 (2C), 54.3, 53.1, 41.9, 32.9, 30.3, 26.7, 23.9, 22.4, 15.7; ESI-MS [M+H]^+^ *m*/*z*: 613; HR-ESI-MS [M + H]^+^ *m*/*z*: 613.2577, Calcd. for C_32_H_40_N_2_O_8_S (M + H) 613.2539.

*(2S)-4-((1E,6E)-7-(4-Hydroxy-3-methoxyphenyl)-3,5-dioxohepta-1,6-dienyl)-2-methoxyphenyl 2-(2-amino-3-methylbutanamido)-4-methylpentanoate* (**18**). Dark red solid; yield: 89%; m.p. 138–140 °C; ^1^H-NMR (400 MHz, MeOD-*d*_4_) δ: 7.60 (s, 2H), 7.34 (s, 1H), 7.22 (s, 2H), 7.11 (d, *J* = 7.6 Hz, 2H), 6.84 (d, *J* = 6.6 Hz, 2H), 6.67 (s, 1H), 4.77 (m, 1H), 3.90 (d, *J* = 17.0 Hz, 6H), 3.78 (dd, *J* = 8.3, 5.4 Hz, 1H), 2.26 (m, 1H), 1.87 (m, 3H), 1.05 (m, 12H); ^13^C-NMR (100 MHz, MeOD-*d*_4_) δ: 185.2, 181.2, 170.3, 168.4, 151.4, 149.3, 148.1, 141.6, 140.8, 138.8, 134.6, 127.1, 124.3, 122.9, 122.6, 120.9, 120.7, 115.2, 111.4, 110.5, 99.9, 58.4, 58.2 , 55.1, 51.0, 39.9, 30.3, 24.6, 21.9, 20.3, 17.5, 16.3; ESI-MS [M+H]^+^ *m*/*z*: 581; HR-ESI-MS [M + H]^+^ *m*/*z*: 581.2881, Calcd. for C_32_H_40_N_2_O_8_ (M + H) 581.2818.

### 3.2. Stability of Derivatives in Plasma in Vitro

Stock solutions of curcumin and dexamethasone acetate (IS) were prepared in methanol of 500 μg/mL and 255 μg/mL, respectively. Working solutions of curcumin between 2.9 and 500 μg/mL were prepared by diluting the stock solution with methanol. A 25.5 μg/mL working solution of IS was similarly prepared. All solutions were stored at −20 °C and brought to room temperature before use. Calibration plasma samples covered the concentration range at 2.9, 5, 29, 72.5, 125, 250, and 500 μg/mL. Calibration plasma sample preparation was same as the plasma samples.

Next, 30 µL of 25 μg/mL dexamethasone acetate (IS), 10 µL of test solution (1.1 mmol/L) of curcumin, **3**, **8**, **10**, and **12** in methanol solution or **4** in 90% methanol–water solution, or blank solvent (methanol or 90% methanol–water) was added to 90 µL of rabbit plasma. The plasma sample was vortexed for 1 min and then incubated for different times at 37 °C *in vitro*. The produced curcumin was extracted with 600 µL of ethyl acetate, followed by vortexing for 2 min and centrifuging at 6000 rpm for 10 min. The supernatant was volatilized to dryness in a 1.5mL EP tube. The obtained residue was redissolved with fresh solvent (methanol or 90% methanol–water) and vortexed for 60 s. The supernatant (20 µL) was directly injected into an HPLC system after high-speed centrifugation at 10,000 rpm for 5 min.

Curcumin content in the plasma was determined using a slight modification of a reported HPLC method [21]. A C18 column (250 mm × 4.6 mm; 5 µm) was used and the mobile phase, 50% acetonitrile and 50% water, was run at a flow rate of 1.0 mL/min. The column effluent was monitored with a UV detector (Shimadzu, Kyoto, Japan) at 260 nm.

### 3.3. Biological Activity Evaluation

#### 3.3.1. Antitumor Cell Line Growth Activity of Curcumin Derivatives

HeLa, MCF-7, and Hep-G2 cells were obtained from the American Type Culture Collection (ATCC; Rockville, MD, USA). RPMI-1640 and Dulbecco’s modified Eagle’s medium (DMEM) tissue culture medium, penicillin-streptomycin, and L-glutamine were from HyClone (Beijing, China). Fetal bovine serum was from Gibco (Grand Island, NY, USA). MCF-7 and Hep-G2 cells were maintained in RPMI-1640 culture medium, and HeLa cells were cultured in DMEM medium; all media were supplemented with 5% heat inactivated serum, 100 U/mL penicillin, and 100 mg/mL streptomycin. Curcumin derivatives were dissolved in dimethyl sulfoxide (DMSO) and diluted with respective medium to final concentrations of 10 mM. The concentration of DMSO used in each case never exceeded 0.1%.

Each cell line was seeded into 96-well plates at a density of 4000 cells per well in the respective medium and incubated at 37 °C under 5% CO_2_ for 12 h. Cells were treated with test compounds at different concentrations from 2.5 µM to 80 µM for 72 h, and then 20 µL MTT (5 mg/mL in phosphate-buffered saline (PBS)) was added in each well, followed by incubation in a CO_2_ incubator for 4 h. Cells were dissolved with 100 µL DMSO and analyzed in a multi-well plate reader at 570 nm. The IC_50_ values were calculated according to the inhibition ratio of the cells.

#### 3.3.2. Morphological Analysis with DAPI

HeLa cells were incubated with different concentrations (0, 2.5, 5, and 10 µM) of compound **3** at 37 °C under 5% CO_2_ for 48 h. The cells were washed twice with cold PBS, fixed with 200 µL acetone–methanol (1:1) for 5 min, and then incubated with 50 μg/mL of DAPI for 10 min. Cells were then washed five times with PBS-TX for 3 min each, and then 200 µL of PBS was added. The nuclear morphology and organization of cytoskeleton were imaged by an inversion fluorescence microscope (Olympus IX71, Olympus Corporation, Tokyo, Japan).

#### 3.3.3. Cell Apoptosis via Annexin V-FITC/PI Double Staining

HeLa cells were seeded into six-well plates (1 × 10^5^ cells per well) and cultured in RPMI 1640 supplemented with 10% fetal bovine serum at 37 °C in a humidified atmosphere in a 5% CO_2_ incubator. After 24 h, cells were treated with different concentrations of compound **3** (0, 10, 20, and 30 μM) for 24 h. Cells were collected by trypsinization, washed twice with cool PBS, and centrifuged (2000 r/min, 5 min). A binding buffer suspension (200 µL) was added to the cells, followed by 2 µL of the FITC-Annexin V mix, and the resulting mixture was held at 4 °C for 15 min. Next, PI mix (4 µL) was added into the mixture, and the resulting cell suspension was held at 4 °C in the absence of light for 10 min. Cell apoptosis was evaluated by flow cytometry by a BD FACS Caliber instrument (BD Biosciences, San Jose, CA, USA).

## 4. Conclusions

Three series of curcumin derivatives were synthesized by introduction of hydrophilic groups, and their antitumor cell line growth activities were evaluated against three tumor cell lines by MTT assay. Several of these synthesized compounds exhibited potent antitumor cell line growth activities, but most displayed activities similar to or lower than that of curcumin. Compounds **3**, **8**, and **9** exhibited stronger antitumor cell line growth activities against HeLa cells, and compound **12** showed higher antitumor cell line growth activity on MCF-7 cells than curcumin. In the stability assays in plasma *in vitro*, compounds **3** and **4** slowly release curcumin in plasma. Overall, our results showed that compound **3** is more effective than **4** as a potential antitumor agent. Furthermore, Annexin V-FITC/PI double staining and DAPI staining showed that compound **3** could induce cellular apoptosis in a dose-dependent manner. Taken together, these results suggest that compound **3** has potential as a new anticancer drug candidate against HeLa cell like tumors and compound **12** against MCF-7 like breast cancers and both the compounds are worthy of further study.

## Supplementary Materials

Supplementary materials can be accessed at: http://www.mdpi.com/1420-3049/20/12/19772/s1.
