# Supplementary material for: Synthesis and Biological Evaluation of Curcumin Derivatives with Water-Soluble Groups as Potential Antitumor Agents: An in Vitro Investigation Using Tumor Cell Lines
Source: Molecules. 2015 Dec 2;20(12):21501–14. doi: 10.3390/molecules201219772 (PMC6332428; doi:10.3390/molecules201219772)
Supplement: Supplementary file 1 [file molecules-20-19772-s001.pdf]

# Supplementary Materials: Synthesis and Biological Evaluation of Curcumin Derivatives with Water-Soluble Groups as Potential Antitumor Agents: An *in Vitro* Investigation Using Tumor Cell Lines

**Luyang Ding, Shuli Ma, Hongxiang Lou, Longru Sun and Mei Ji**

## 1. Cell Apoptosis Effects of Compounds 8 and 9 on HeLa or 12 on MCF-7

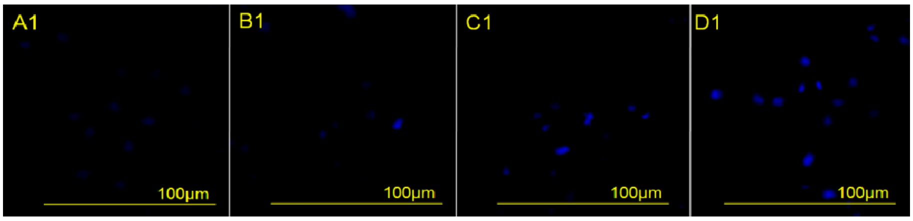


**Figure S1.** Effects of compound **8** on HeLa cell apoptosis was observed by DAPI staining under an inversion fluorescent microscope. HeLa cells were incubated with 0 μM (**A1**); 2.5 μM (**B1**); 5 μM (**C1**); or 10 μM (**D1**) of compound **8** for 48 h.

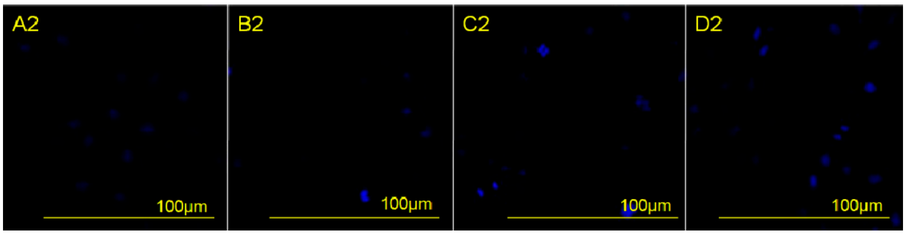


**Figure S2.** Effects of compound **9** on HeLa cell apoptosis was observed by DAPI staining under an inversion fluorescent microscope. HeLa cells were incubated with 0 μM (**A2**); 2.5 μM (**B2**); 5 μM (**C2**); or 10 μM (**D2**) of compound **9** for 48 h.

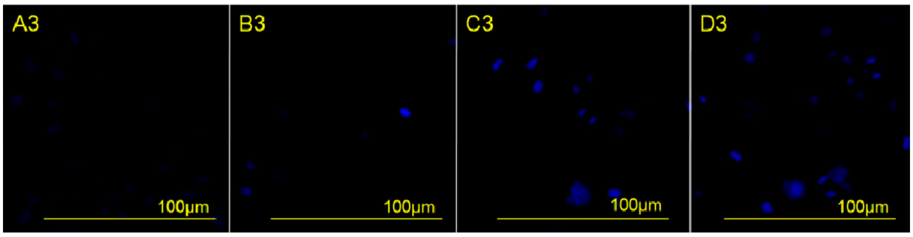


**Figure S3.** Effects of compound **12** on HeLa cell apoptosis was observed by DAPI staining under an inversion fluorescent microscope. HeLa cells were incubated with 0 μM (**A3**); 2.5 μM (**B3**); 5 μM (**C3**); or 10 μM (**D3**) of compound **12** for 48 h.

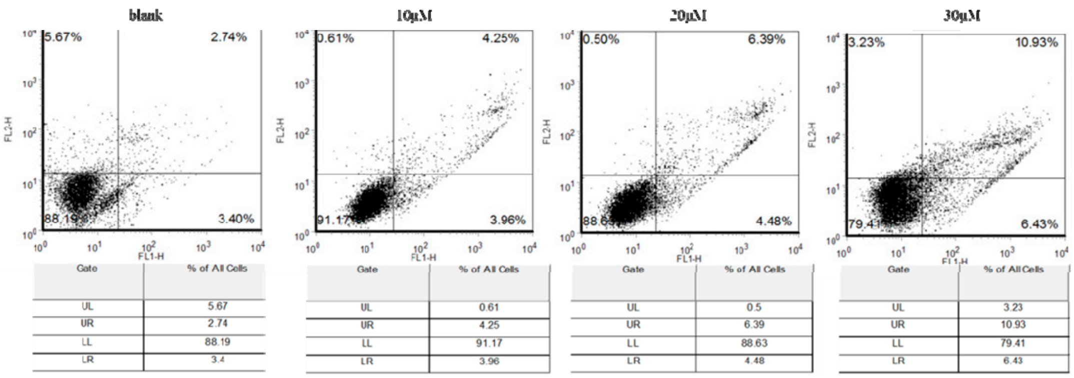


| Gate | % of All Cells |
|---|---|
| UL | 5.67 |
| UR | 2.74 |
| LL | 88.19 |
| LR | 3.4 |

| Gate | % of All Cells |
|---|---|
| UL | 0.61 |
| UR | 4.25 |
| LL | 91.17 |
| LR | 3.96 |

| Gate | % of All Cells |
|---|---|
| UL | 0.5 |
| UR | 6.39 |
| LL | 88.63 |
| LR | 4.48 |

| Gate | % of All Cells |
|---|---|
| UL | 3.23 |
| UR | 10.93 |
| LL | 79.41 |
| LR | 6.43 |

**A**

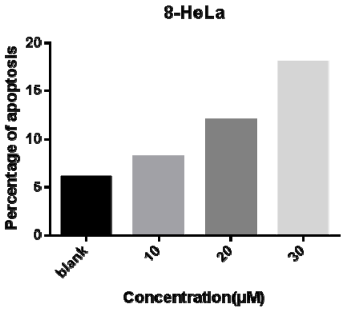


**B**

**Figure S4.** (**A**) HeLa cells were treated with compound **8** (0, 10, 20, or 30 μM) for 24 h and then analyzed by Annexin V-FITC/PI staining and flow cytometry. The apoptotic cell scatter plot is divided into four quadrants: upper left (UL), upper right (UR), lower left (LL), and lower right (LR) quadrants; (**B**) Apoptosis rate of HeLa cells treated with various concentrations of compound **8**.

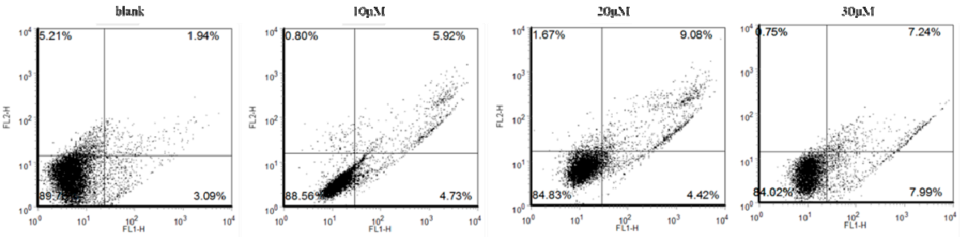


| Gate | % of All Cells |
|---|---|
| UL | 5.21 |
| UR | 1.94 |
| LL | 89.76 |
| LR | 3.09 |

| Gate | % of All Cells |
|---|---|
| UL | 0.8 |
| UR | 5.92 |
| LL | 88.56 |
| LR | 4.73 |

| Gate | % of All Cells |
|---|---|
| UL | 1.67 |
| UR | 9.08 |
| LL | 84.83 |
| LR | 4.42 |

| Gate | % of All Cells |
|---|---|
| UL | 0.75 |
| UR | 7.24 |
| LL | 84.02 |
| LR | 7.99 |

**A**

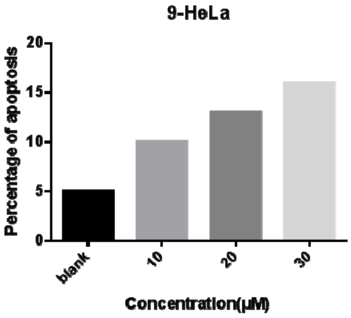


**B**

**Figure S5.** (**A**) HeLa cells were treated with compound **9** (0, 10, 20, or 30 μM) for 24 h and then analyzed by Annexin V-FITC/PI staining and flow cytometry. The apoptotic cell scatter plot is divided into four quadrants: upper left (UL), upper right (UR), lower left (LL), and lower right (LR) quadrants; (**B**) Apoptosis rate of HeLa cells treated with various concentrations of compound **9**.

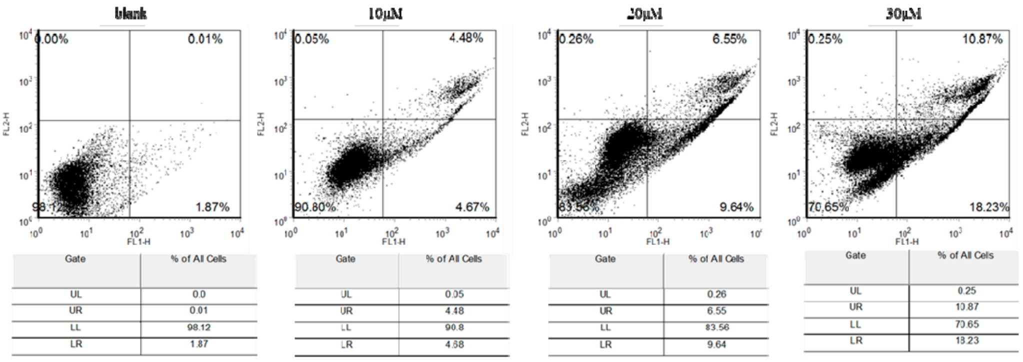


**A**

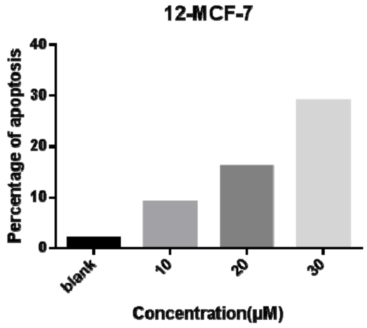


**B**

**Figure S6.** (**A**) MCF-7 cells were treated with compound **12** (0, 10, 20, or 30 μM) for 24 h and then analyzed by Annexin V-FITC/PI staining and flow cytometry. The apoptotic cell scatter plot is divided into four quadrants: upper left (UL), upper right (UR), lower left (LL), and lower right (LR) quadrants. (**B**) Apoptosis rate of MCF-7 cells treated with various concentrations of compound **12**.

## 2. $^1$H-NMR, $^{13}$C-NMR, $^{31}$P-NMR, MS-ESI, and HRMS-ESI of the Synthetic Compounds

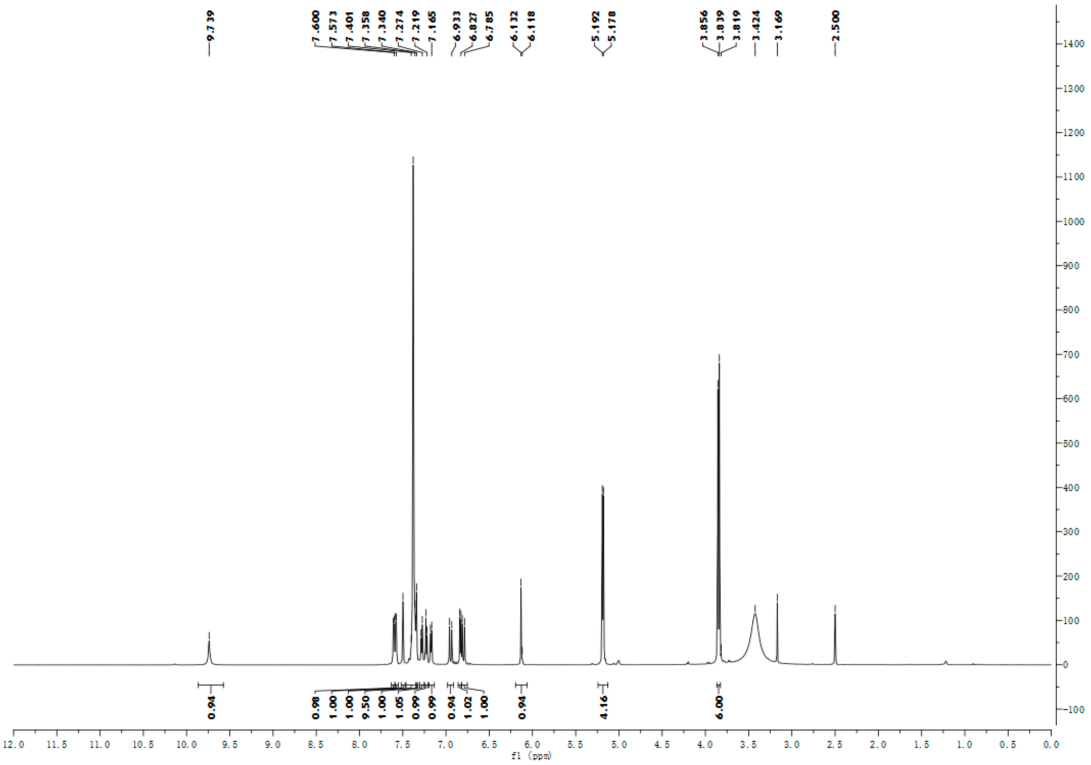


**Figure S7.** $^1$H-NMR for compound **1**.

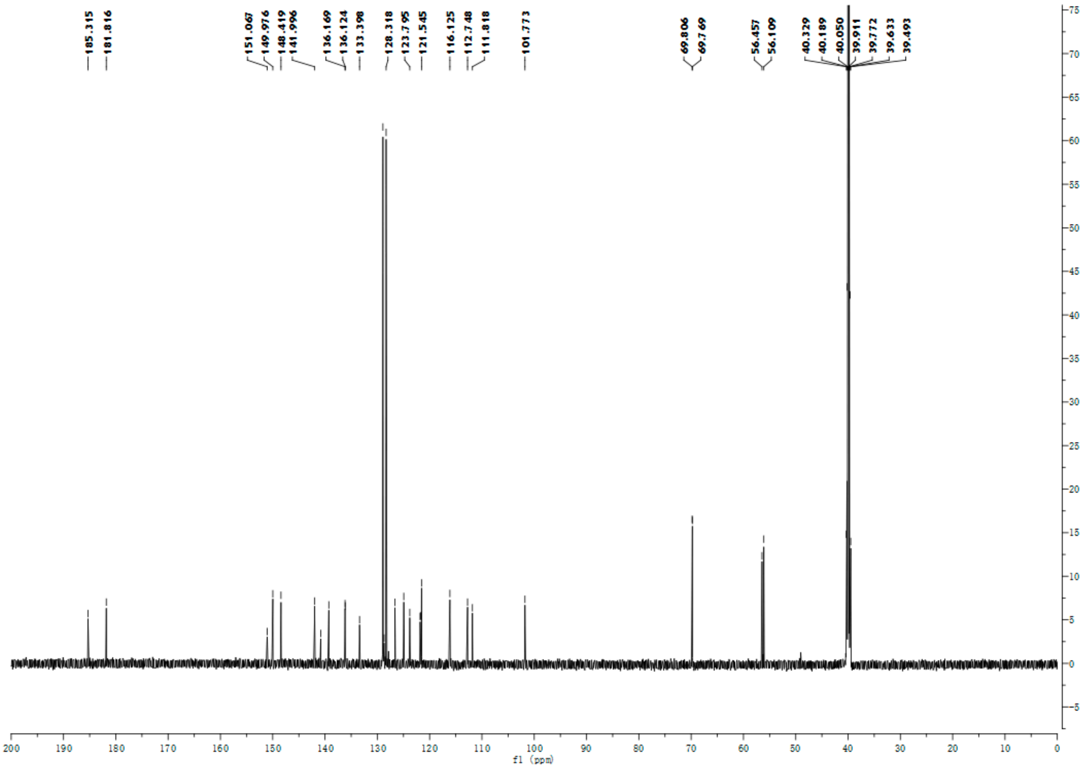


**Figure S8.** $^{13}C$-NMR for compound **1**.

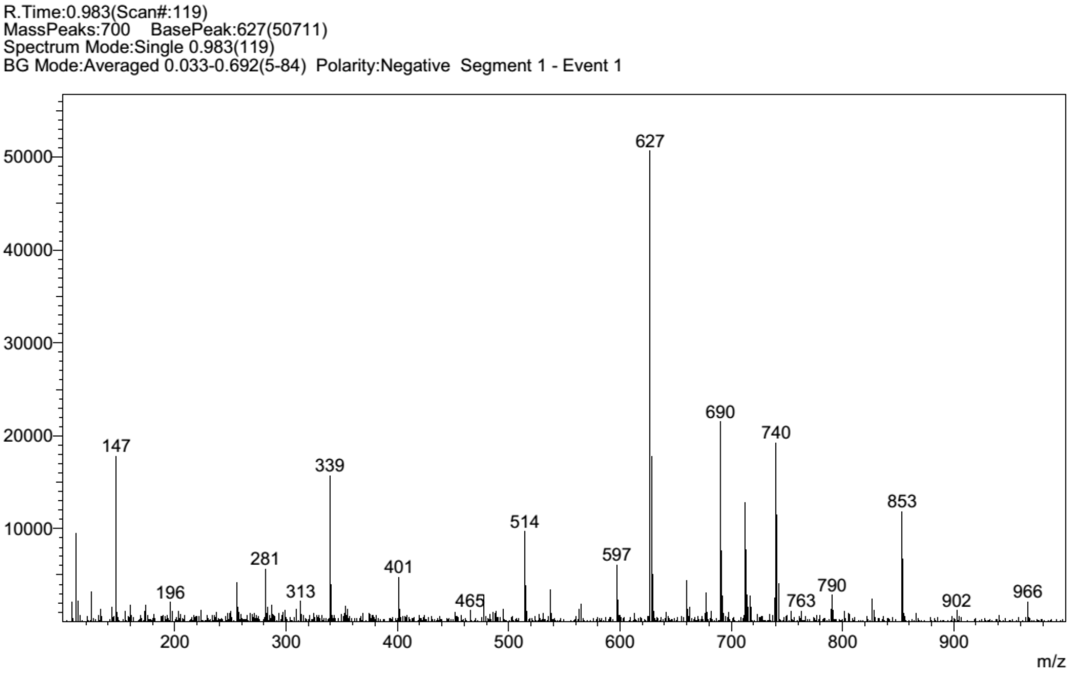


**Figure S9.** MS-ESI for compound **1**.

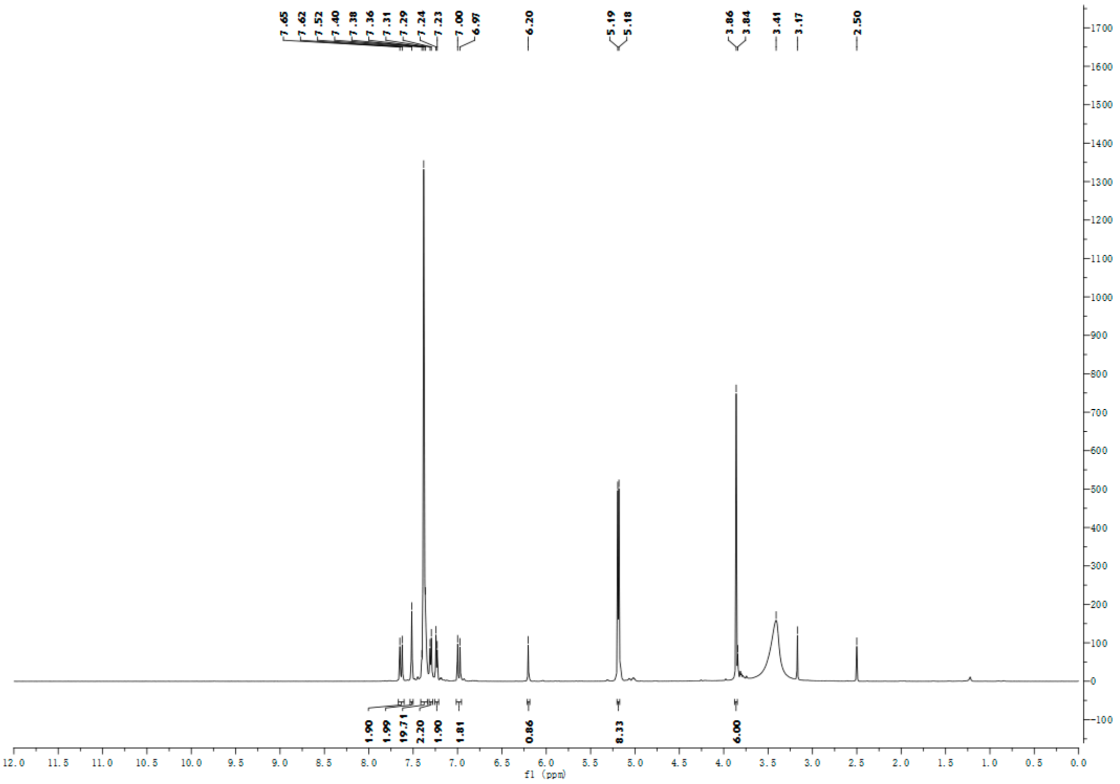


**Figure S10.** $^{1}$H-NMR for compound **2.**

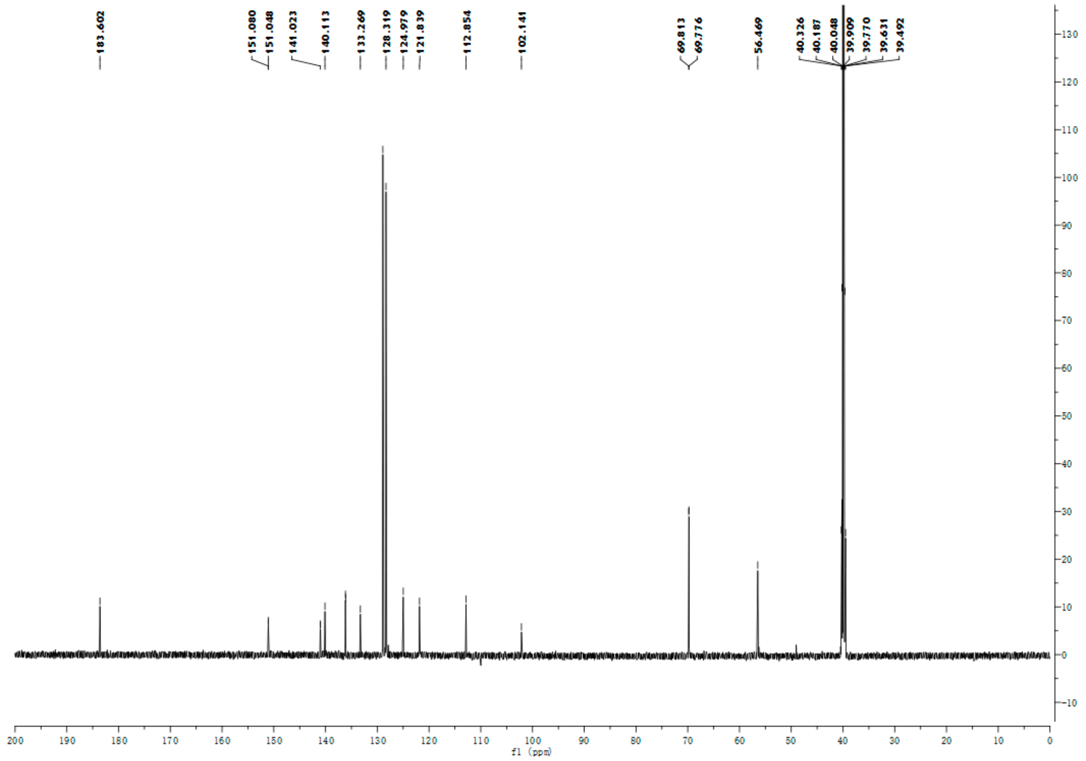


**Figure S11.** $^{13}$C-NMR for compound **2**.

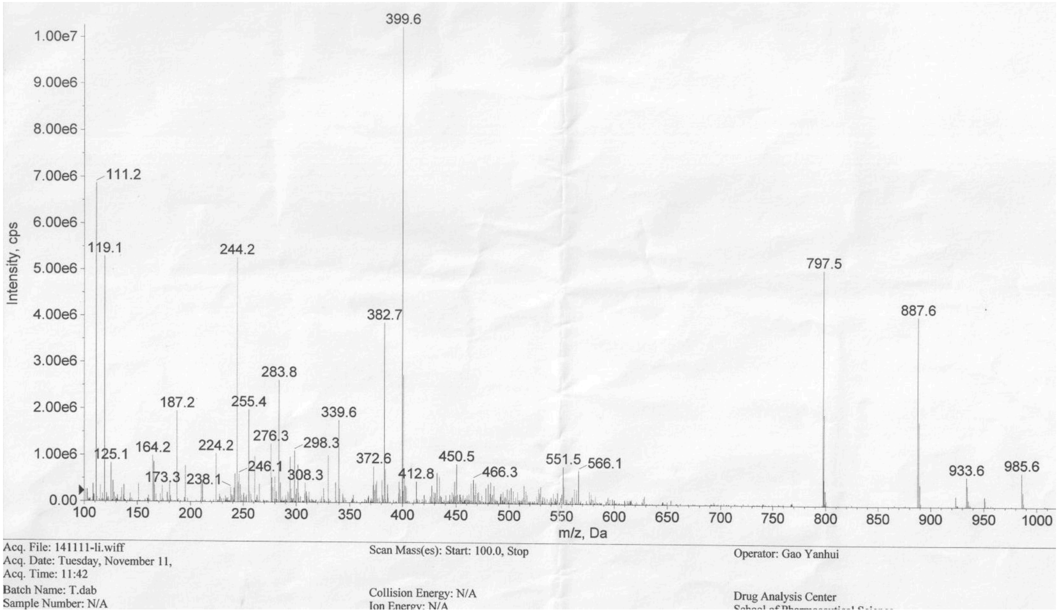


**Figure S12.** MS-ESI for compound **2**.

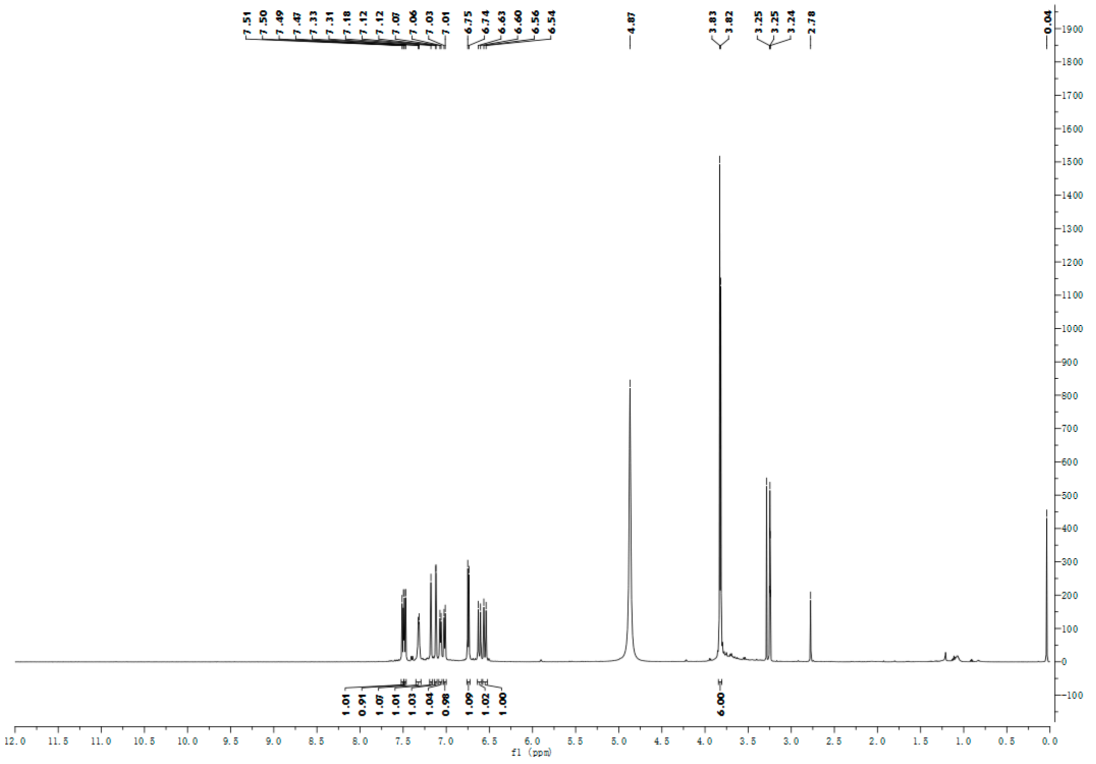


**Figure S13.** $^1$H-NMR for compound **3**.

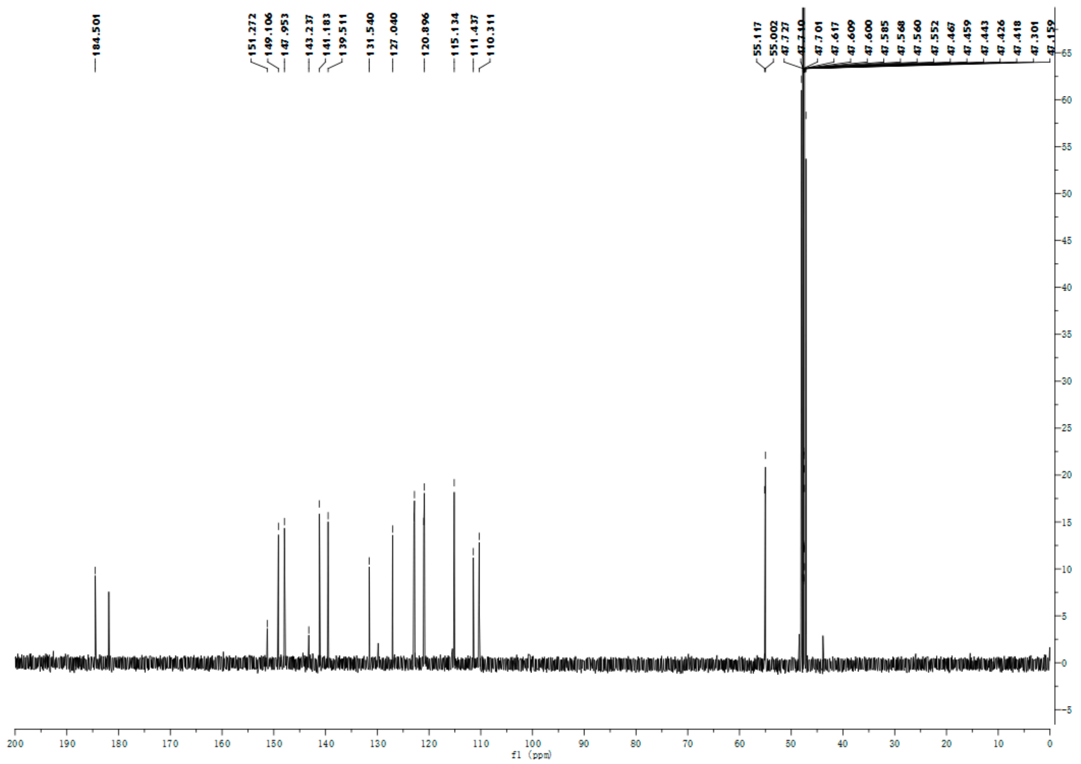

**Figure S14.** $^{13}$C-NMR for compound **3**.

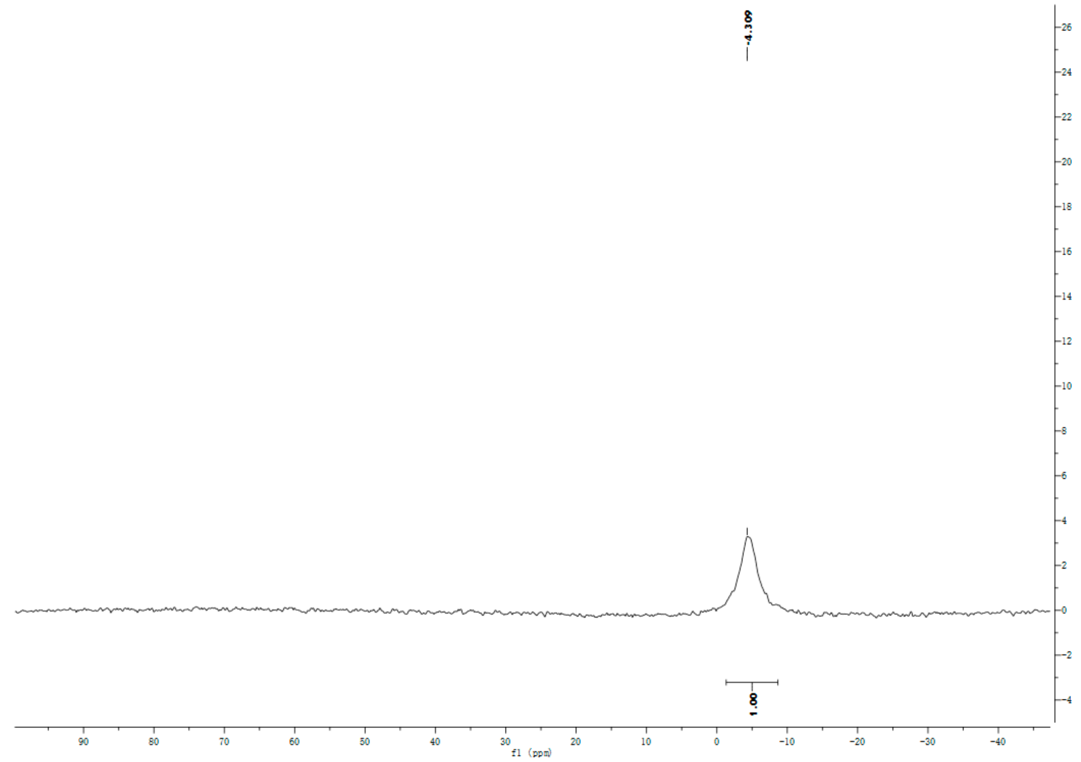

**Figure S15.** $^{31}$P-NMR for compound **3**.

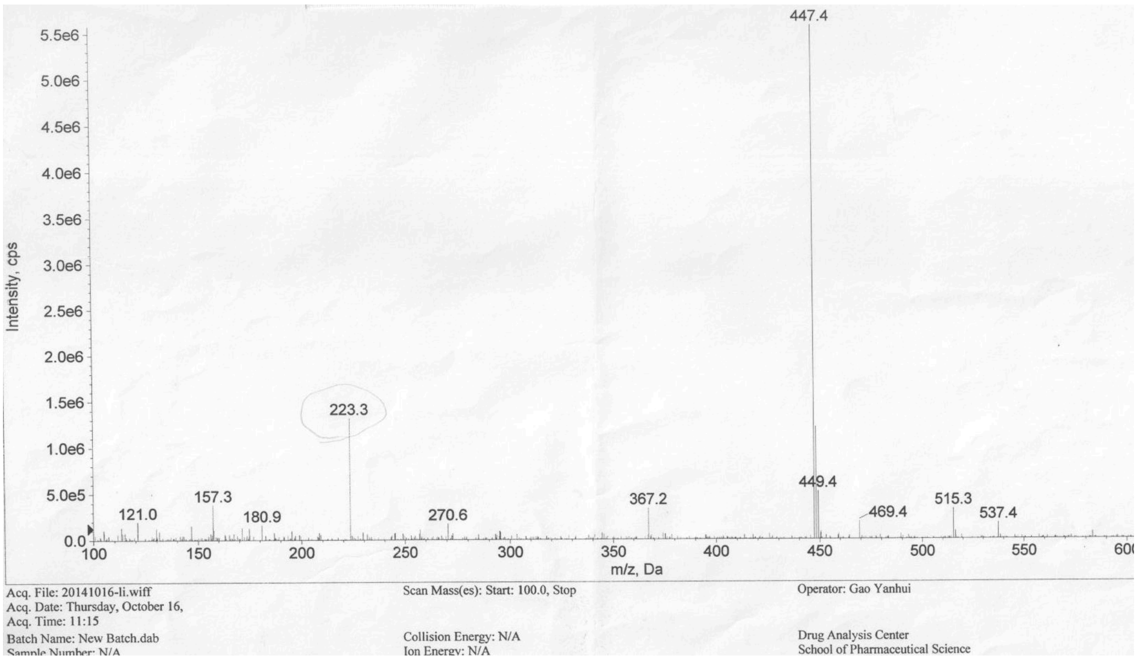


**Figure S16.** MS-ESI for compound **3**.

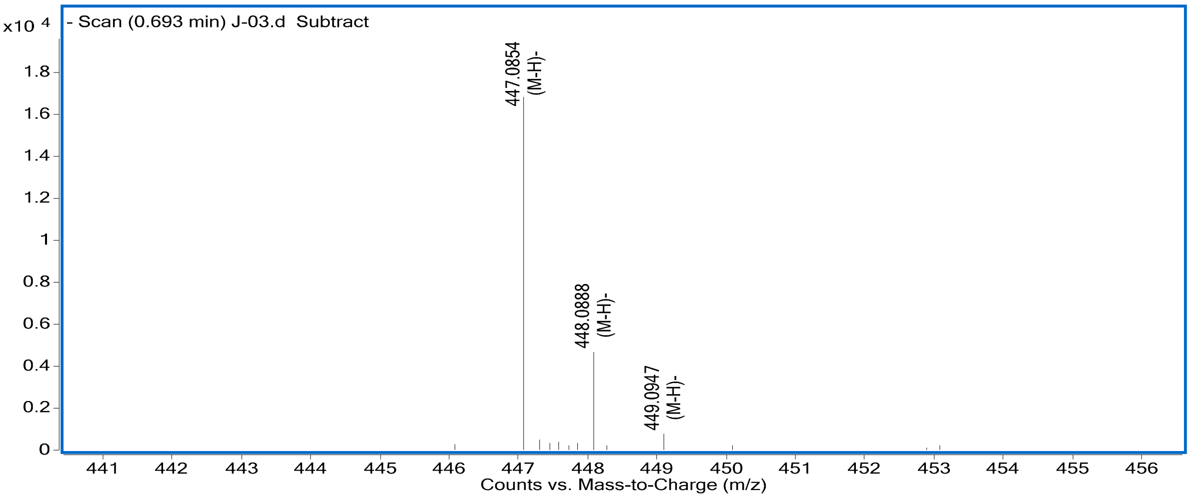


**Figure S17.** HRMS-ESI for compound **3**.

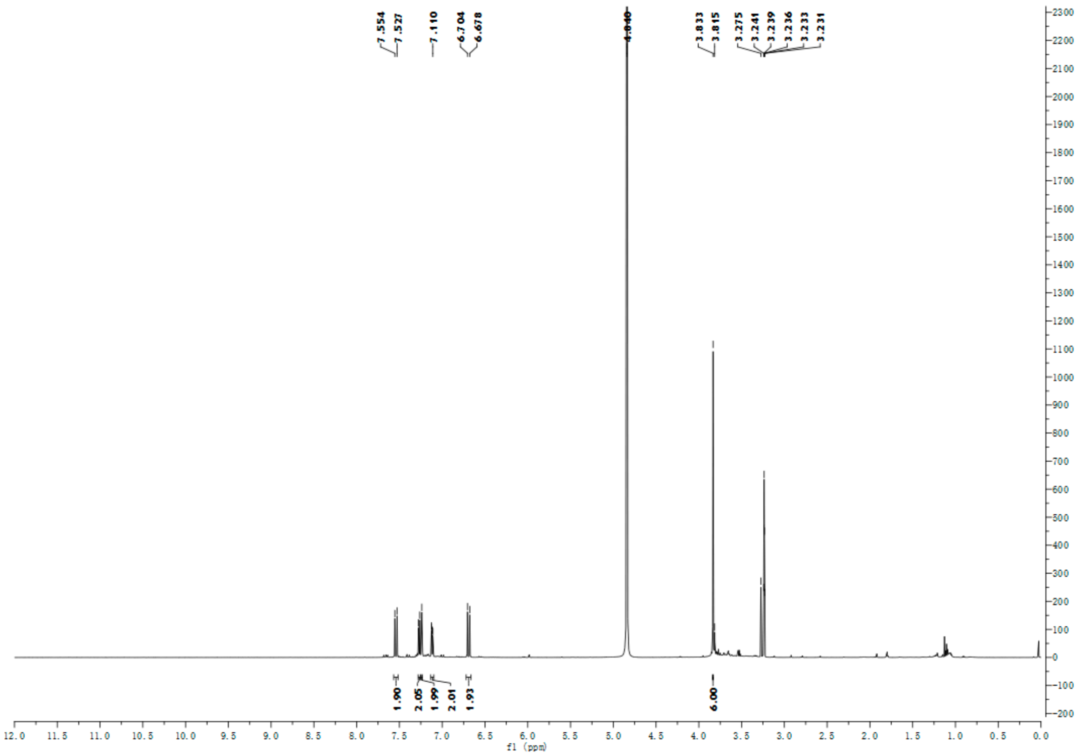


**Figure S18.** $^{1}$H-NMR for compound **4**.

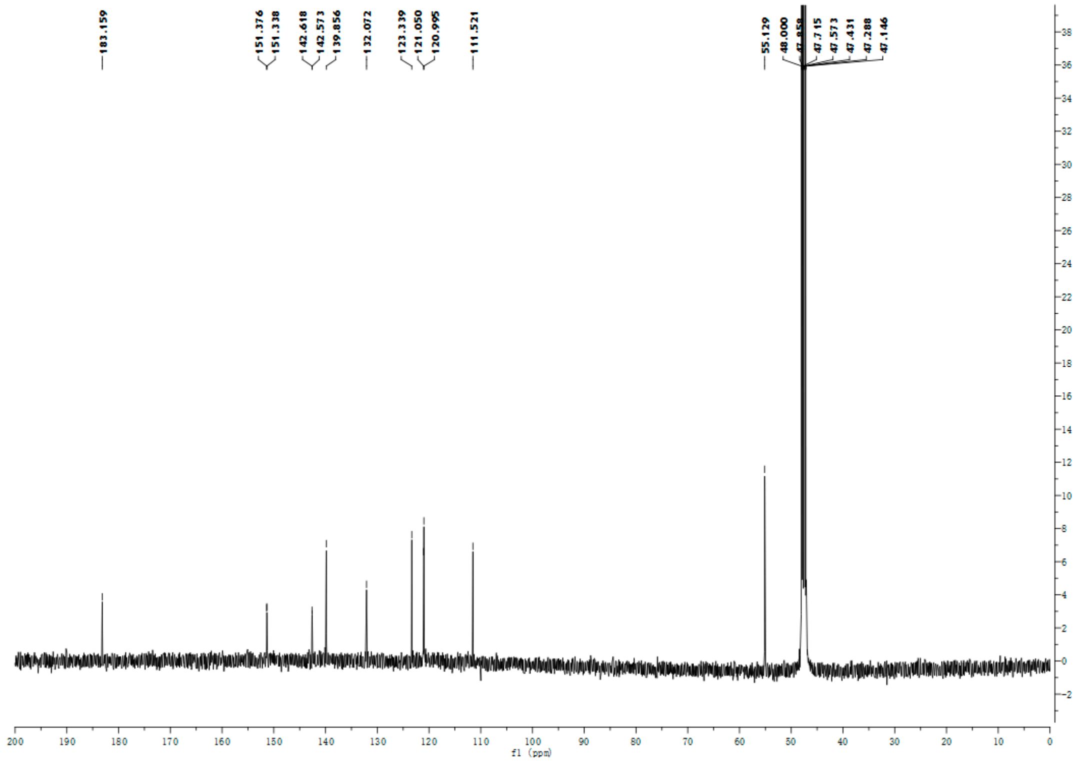


**Figure S19.** $^{13}$C-NMR for compound **4**.

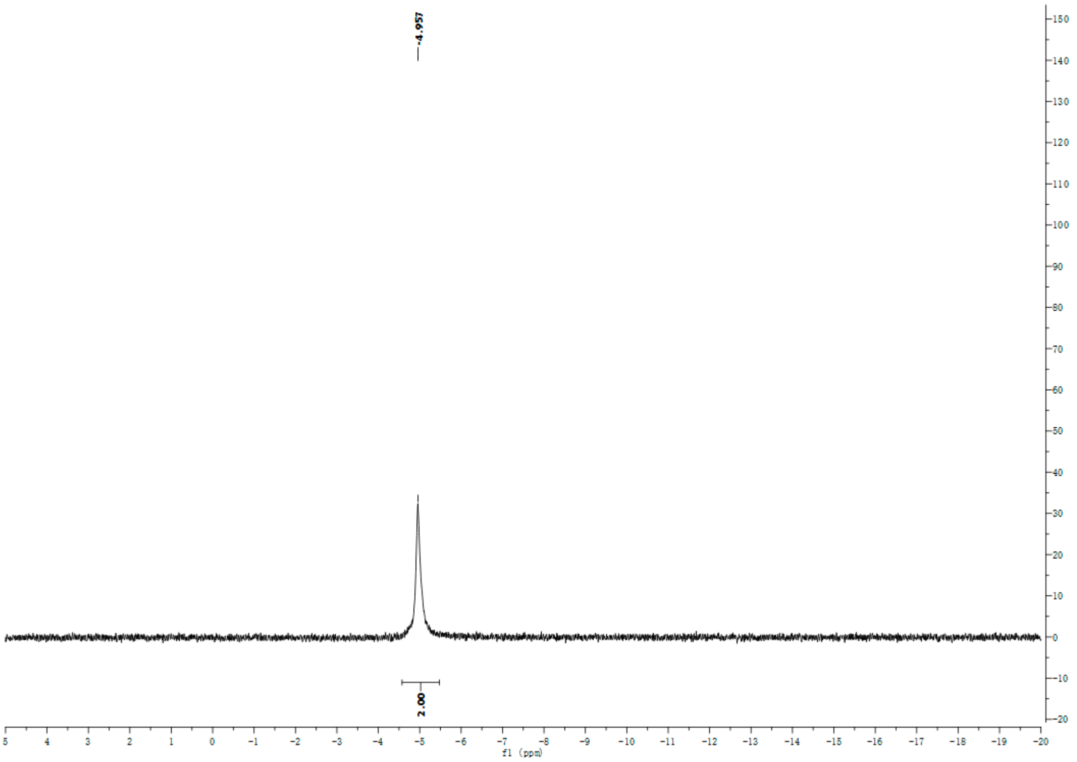


**Figure S20.** $^{31}$P-NMR for compound **4**.

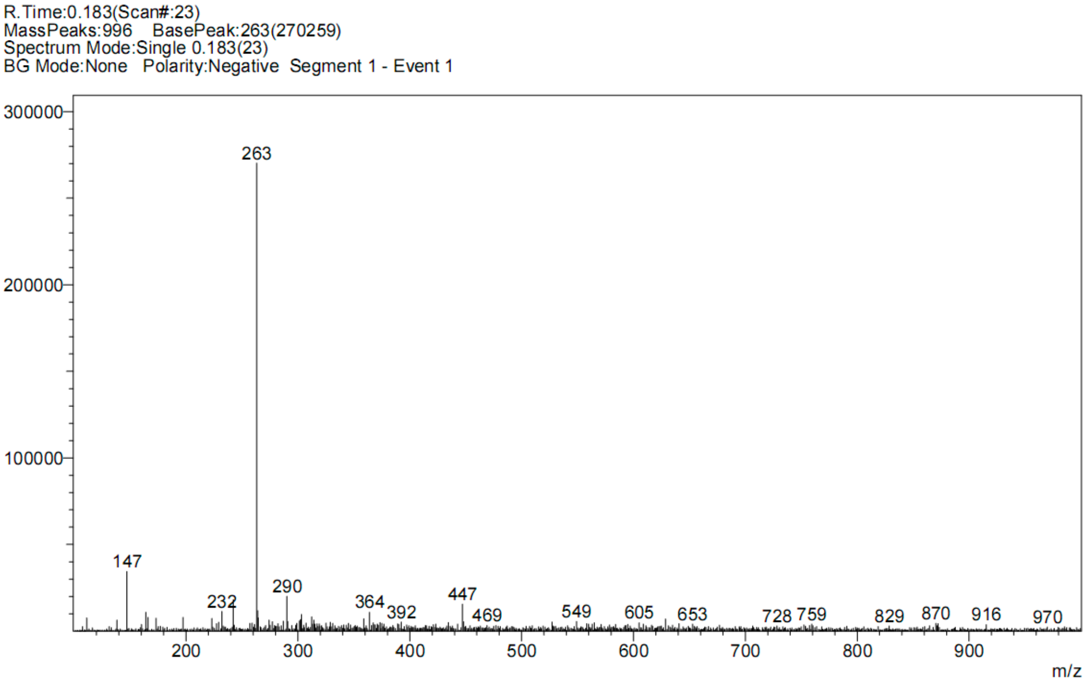


**Figure S21.** MS-ESI for compound **4**.

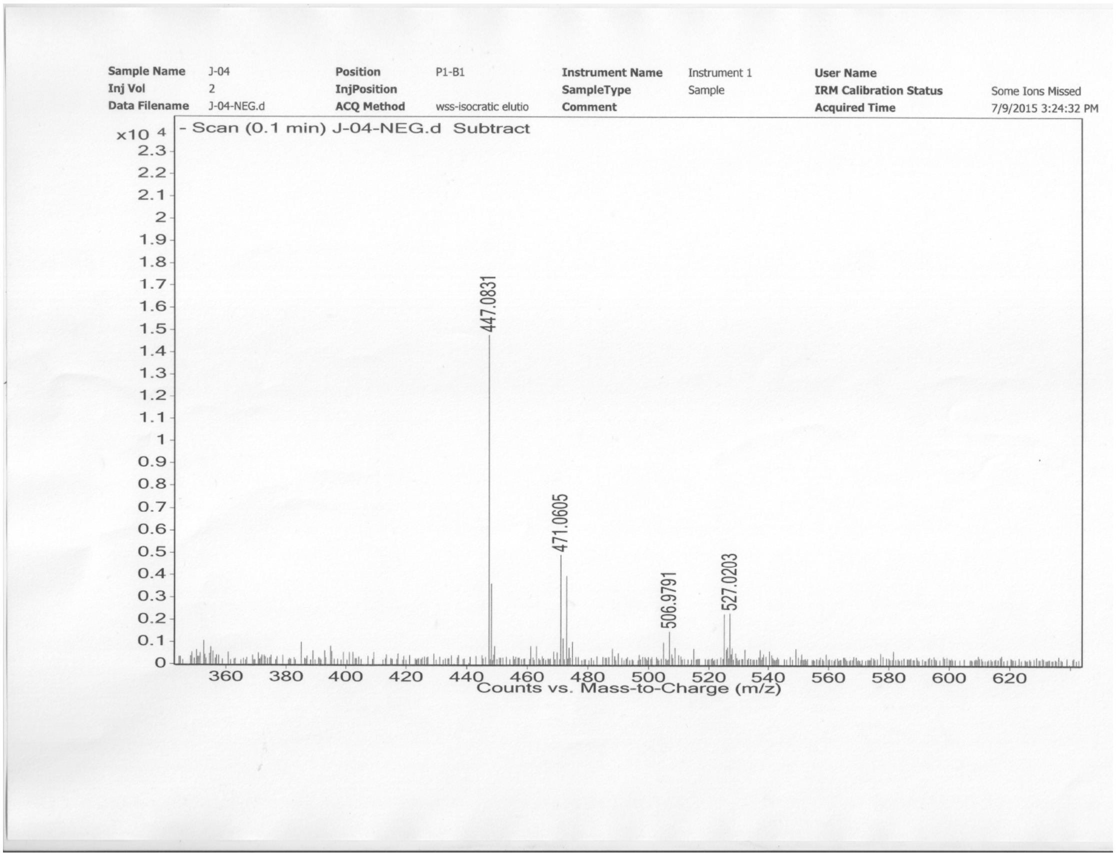


**Figure S22.** HRMS-ESI for compound **4**.

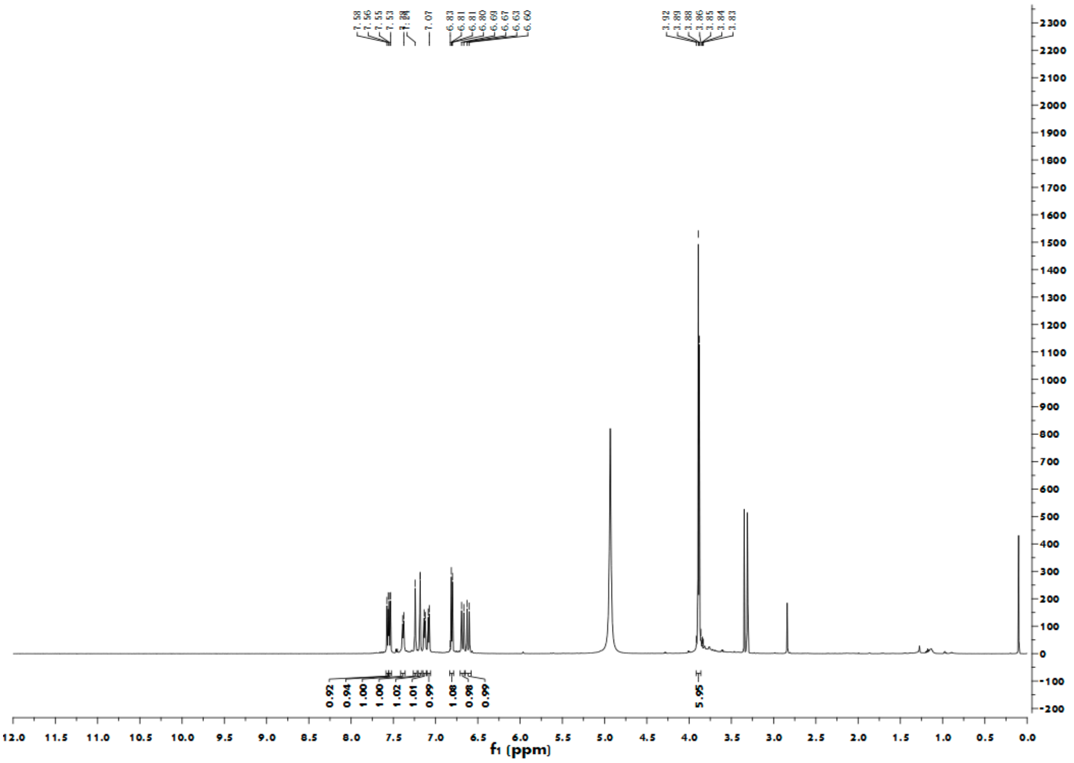


**Figure S23.** $^{1}$H-NMR for compound **5**.

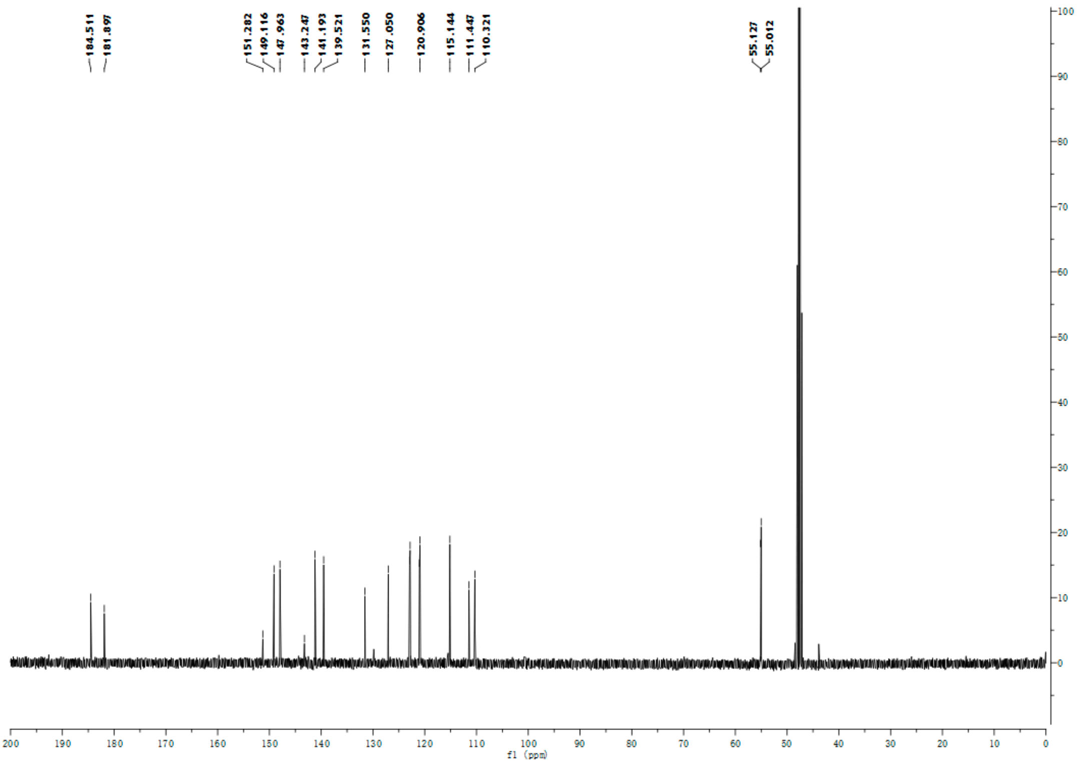


**Figure S24.** $^{13}C$-NMR for compound **5**.

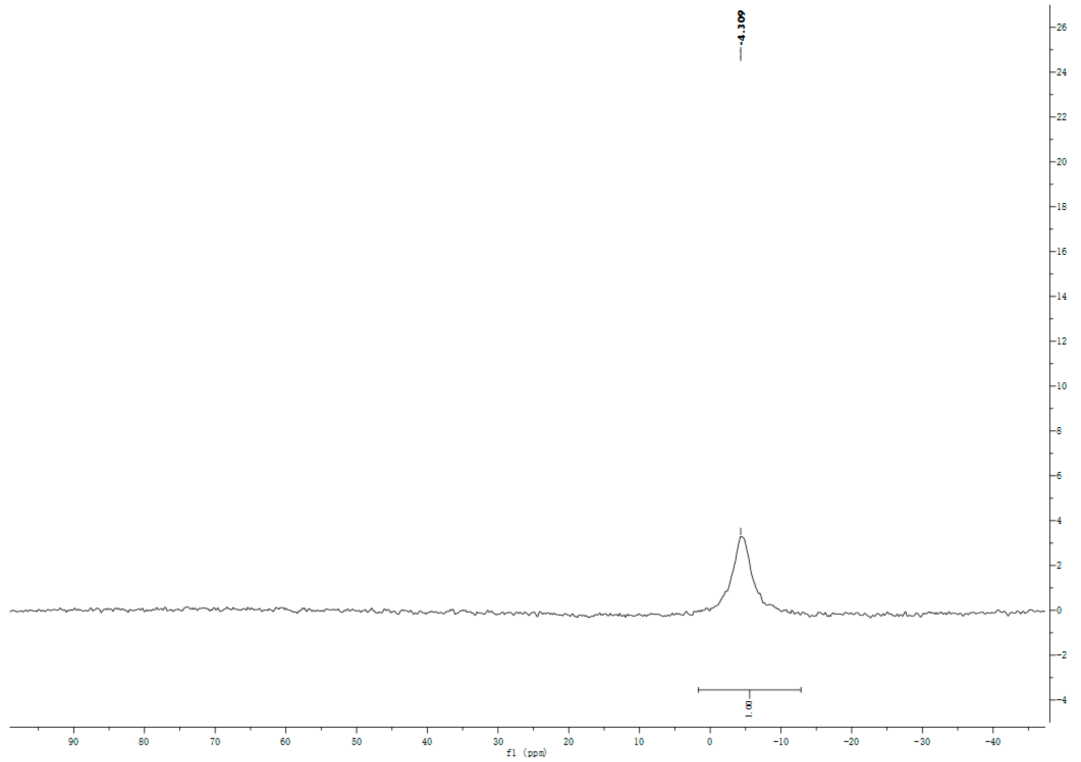


**Figure S25.** $^{31}P$-NMR for compound **5**.

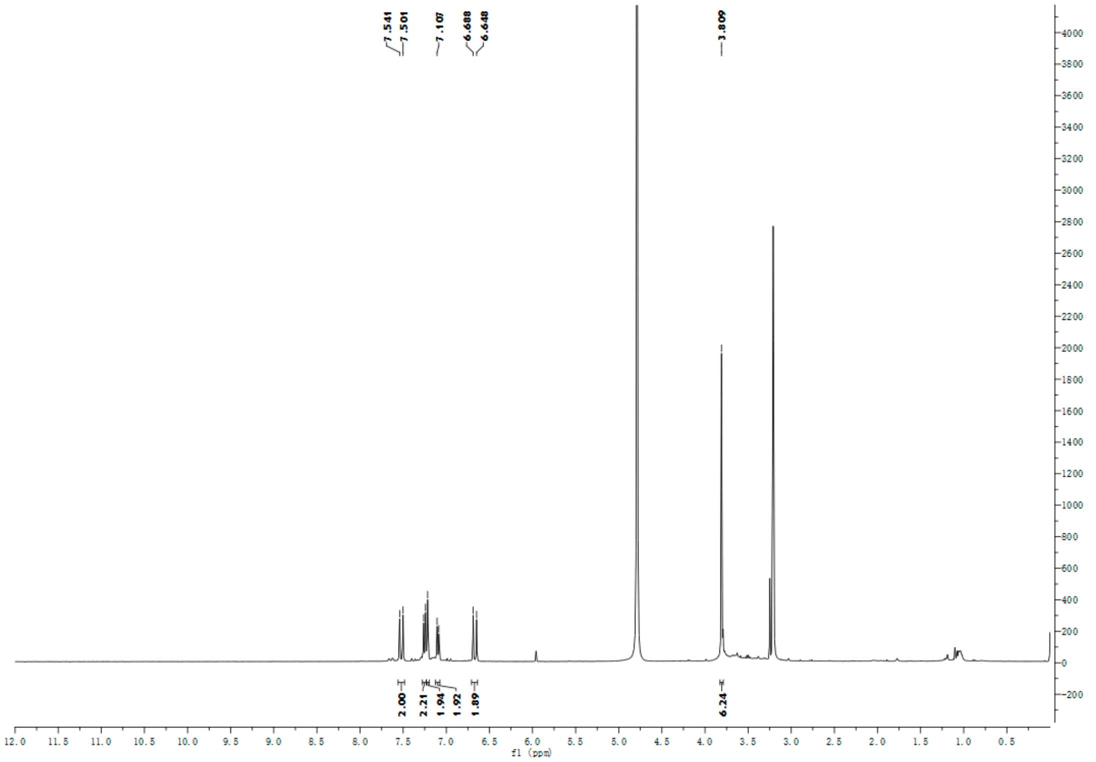


**Figure S26.** $^{1}$H-NMR for compound **6**.

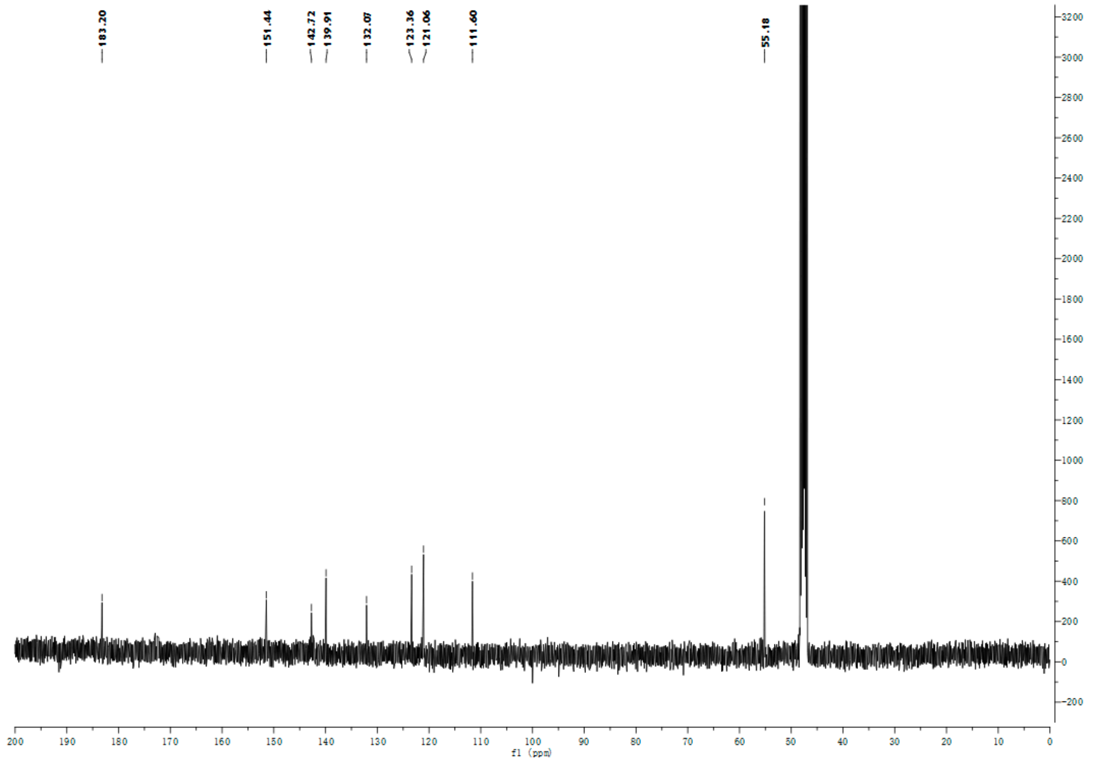


**Figure S27.** $^{13}$C-NMR for compound **6**.

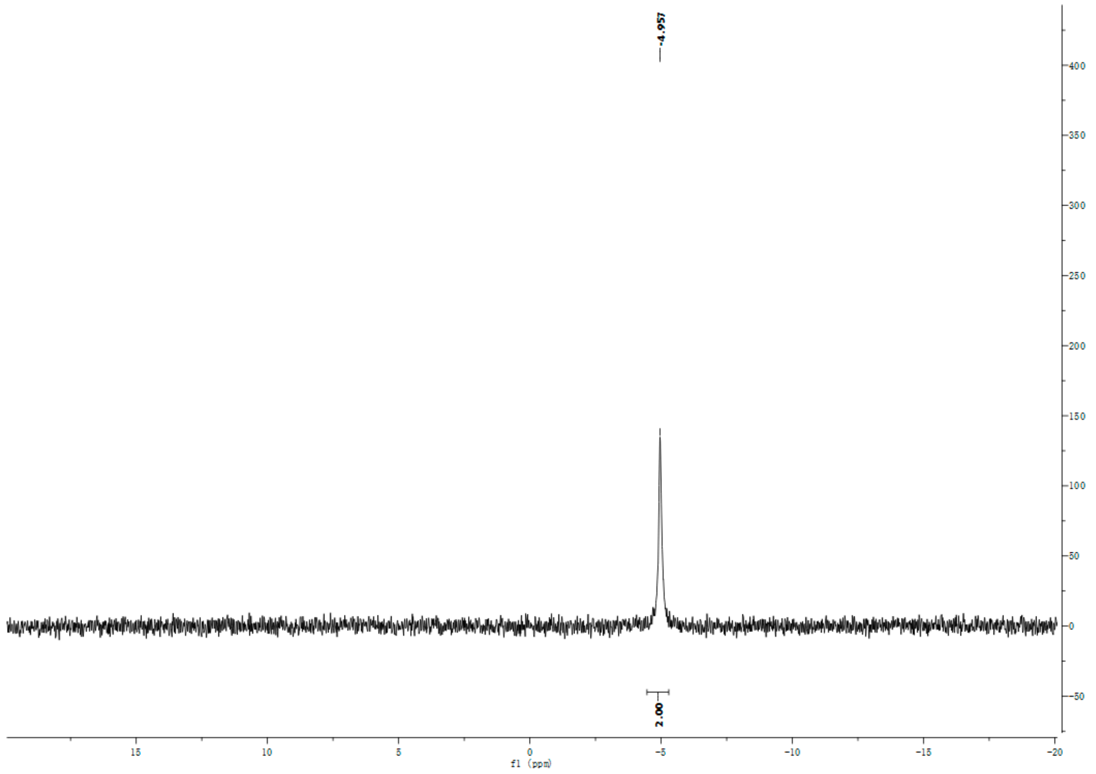

**Figure S28.** $^{31}$P-NMR for compound **6**.

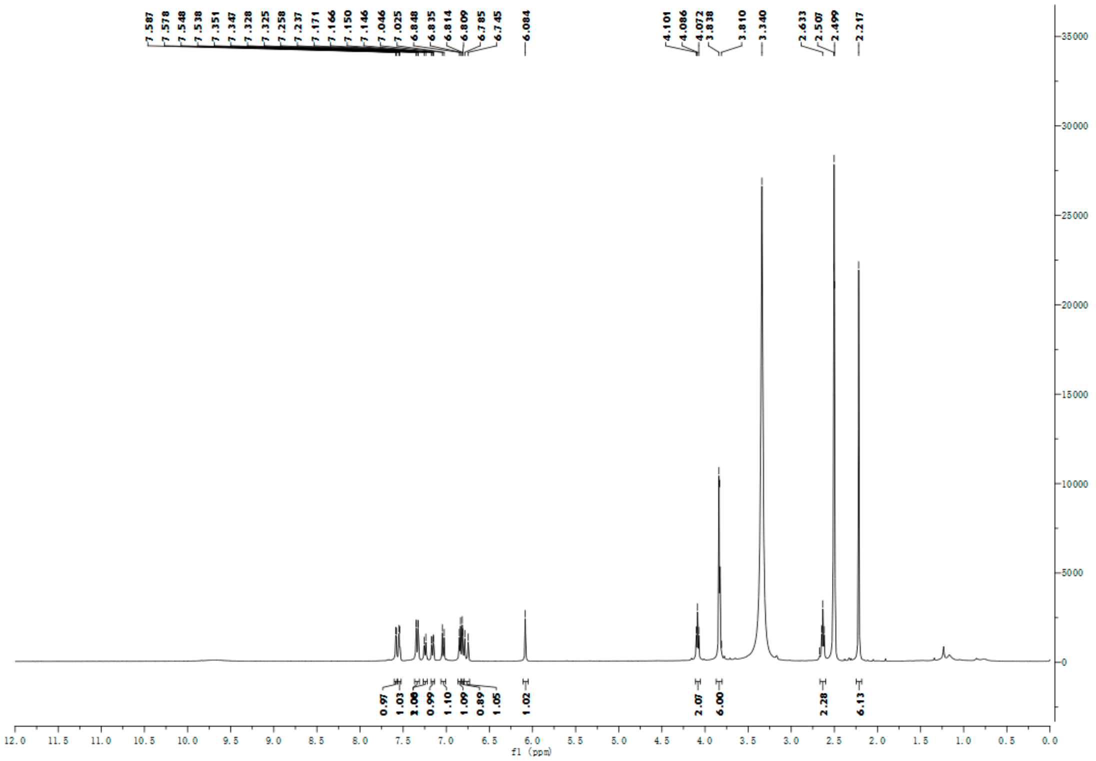

**Figure S29.** $^{1}$H-NMR for compound **7**.

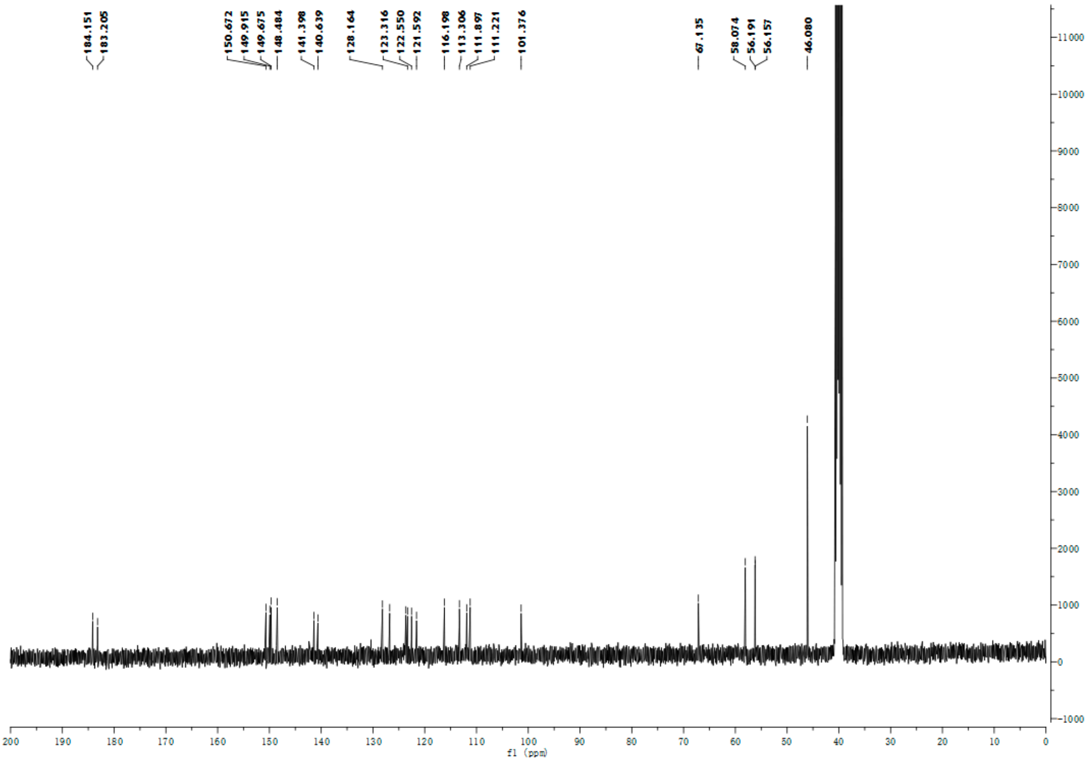


**Figure S30.** $^{13}$C-NMR for compound **7**.

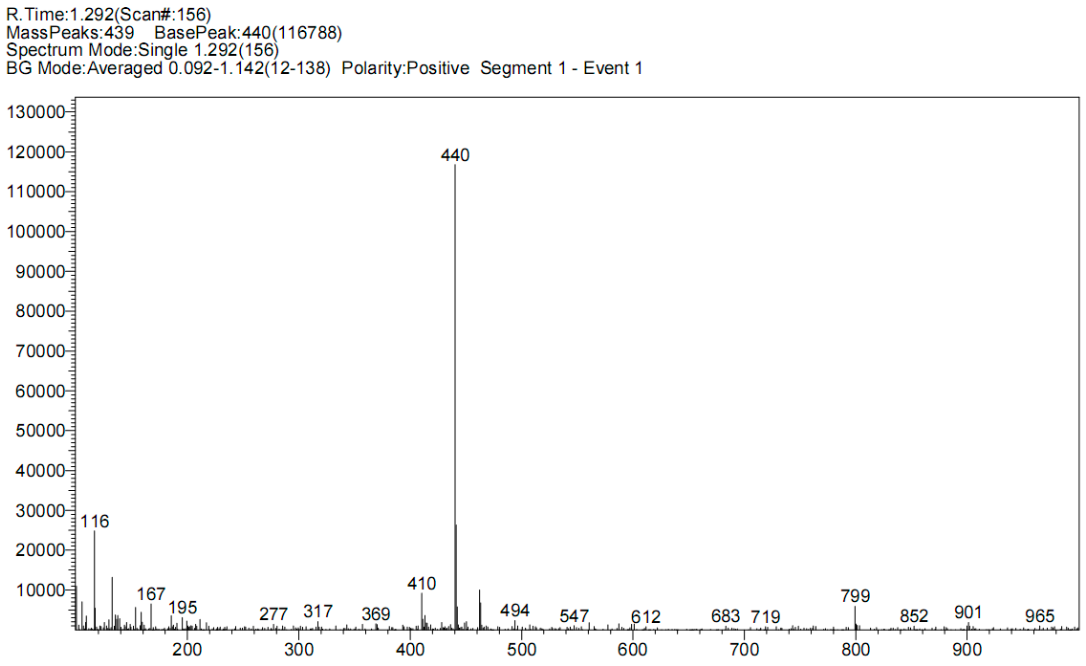


**Figure S31.** MS-ESI for compound **7**.

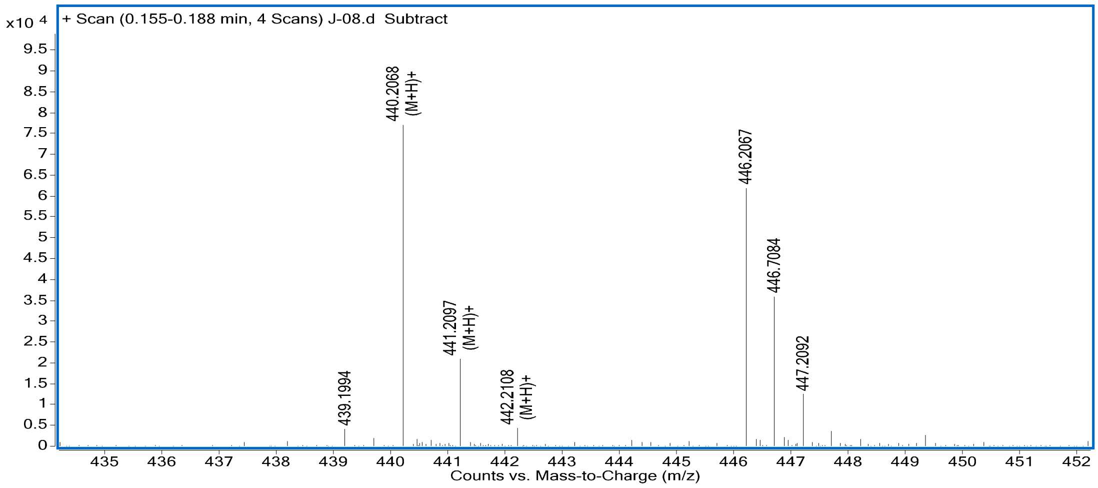


**Figure S32.** HRMS-ESI for compound **7**.

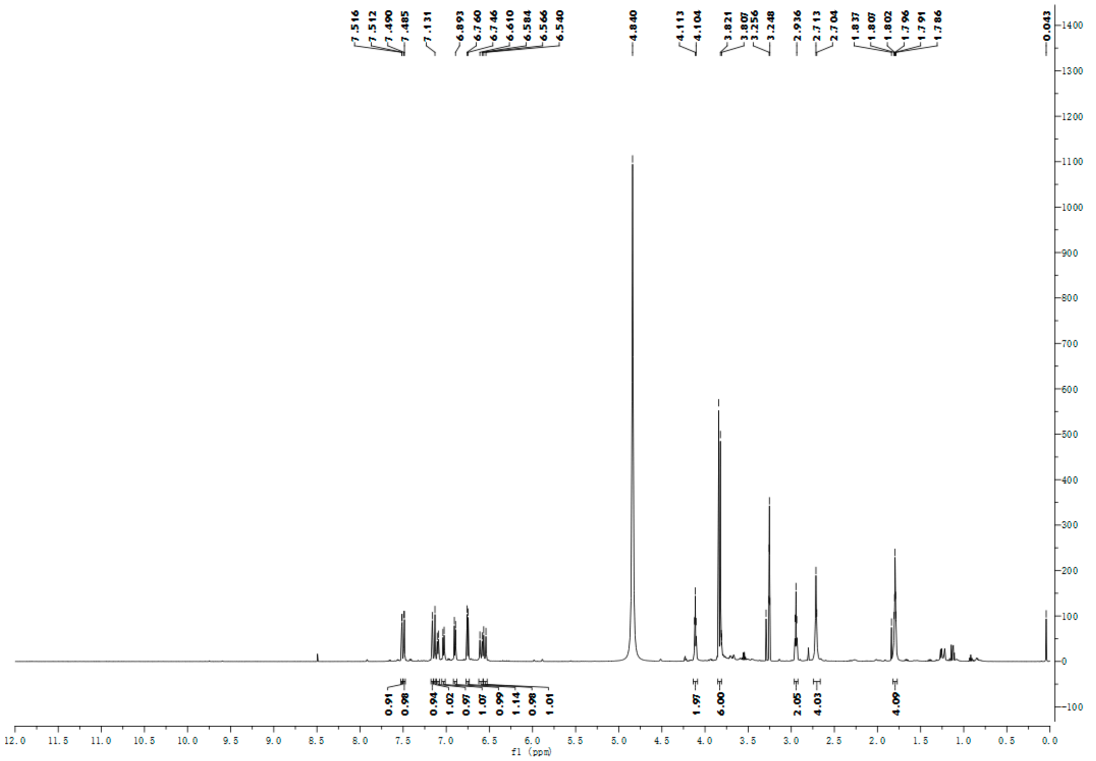


**Figure S33.** $^{1}$H-NMR for compound **8**.

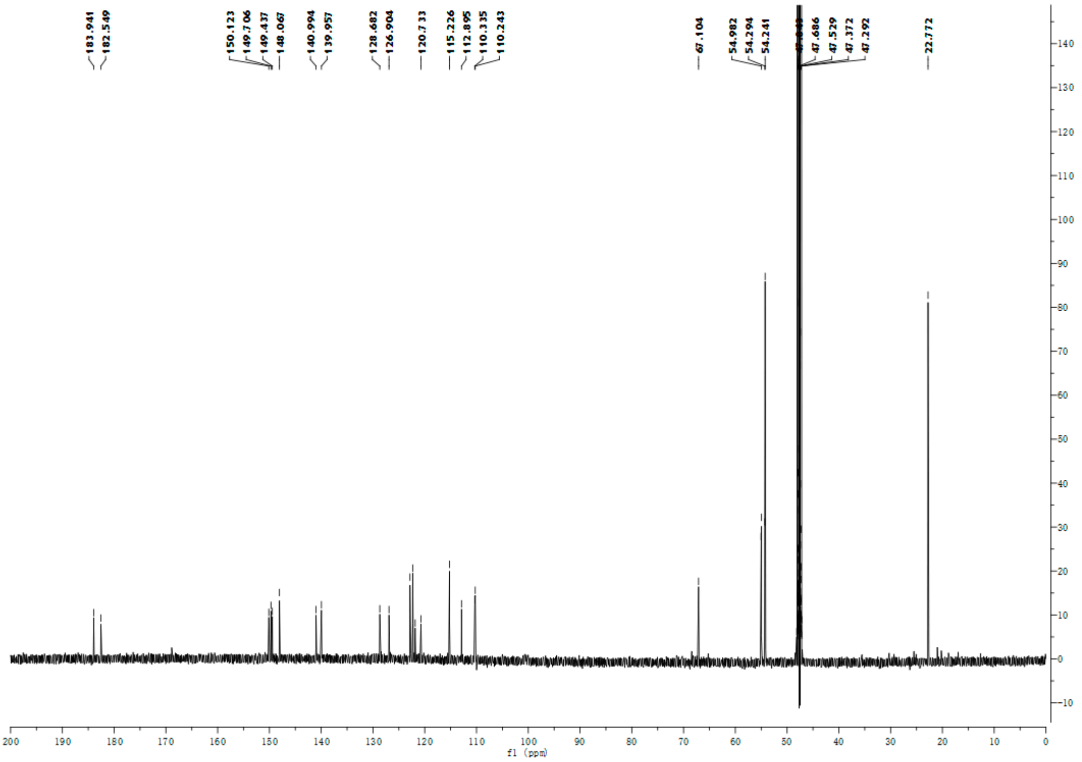


**Figure S34.** $^{13}$C-NMR for compound **8**.

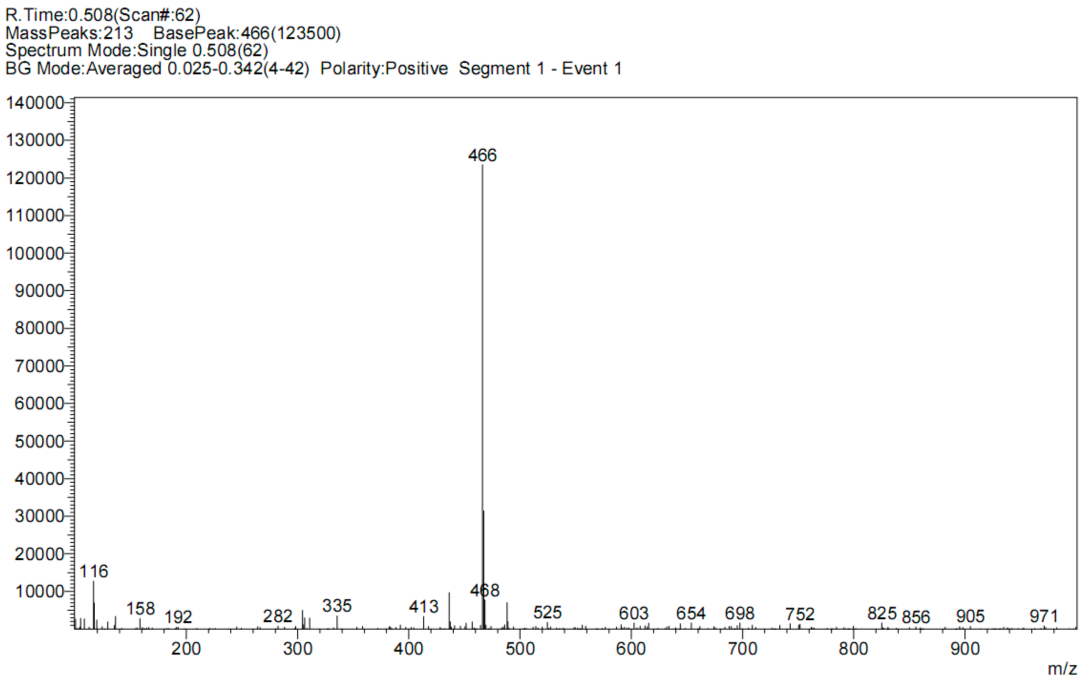


**Figure S35.** MS-ESI for compound **8**.

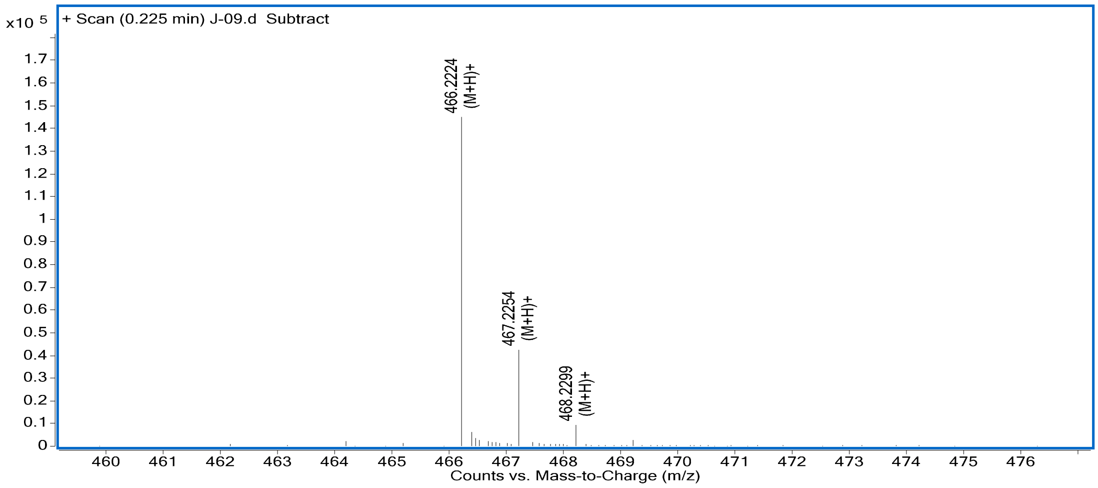


**Figure S36.** HRMS-ESI for compound **8**.

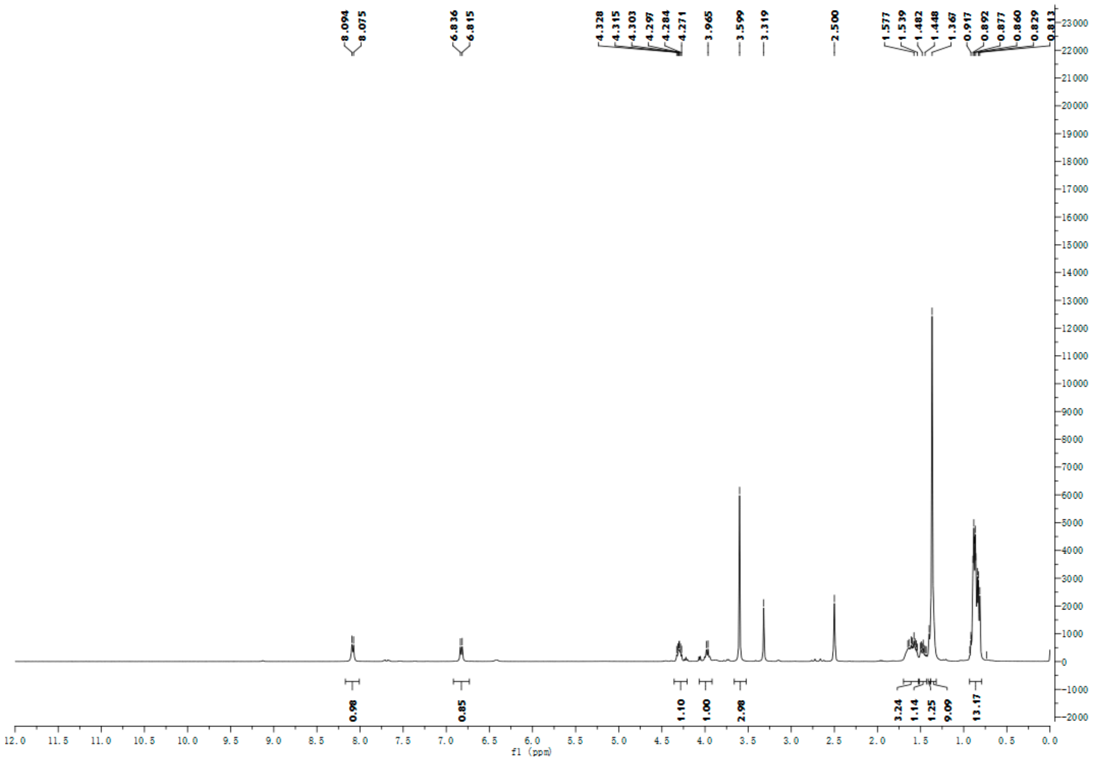


**Figure S37.** $^{1}$H-NMR for compound **Leu-01**.

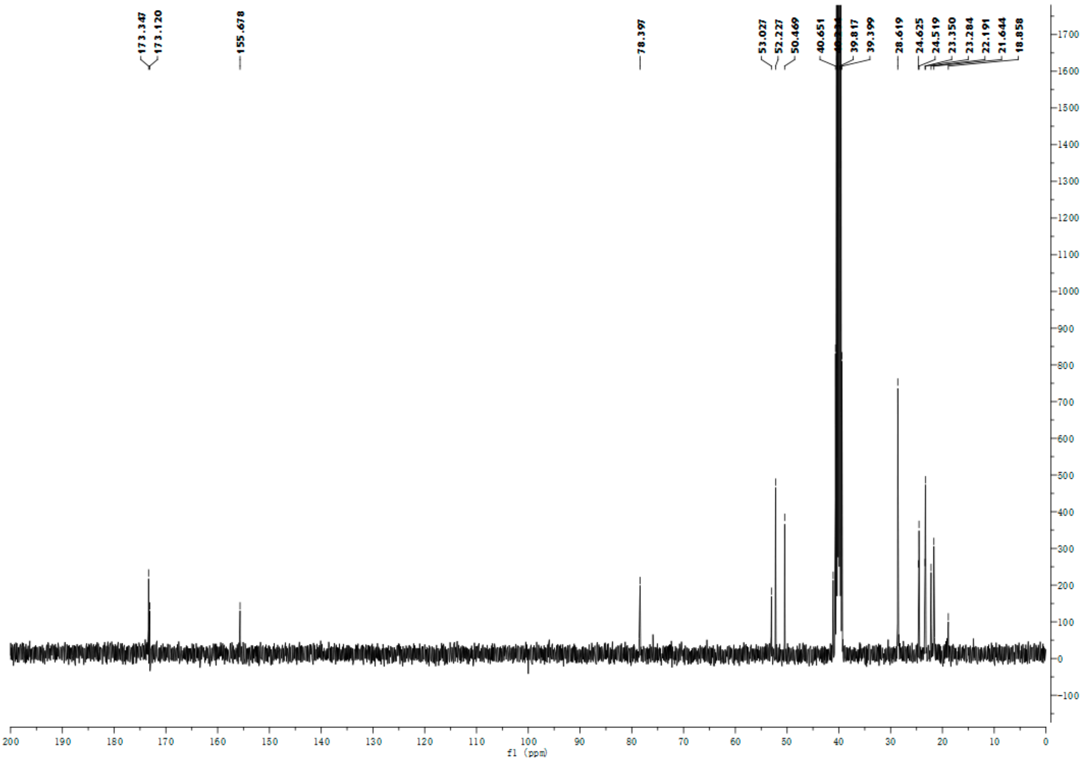


**Figure S38.** $^{13}C$-NMR for compound **Leu-01**.

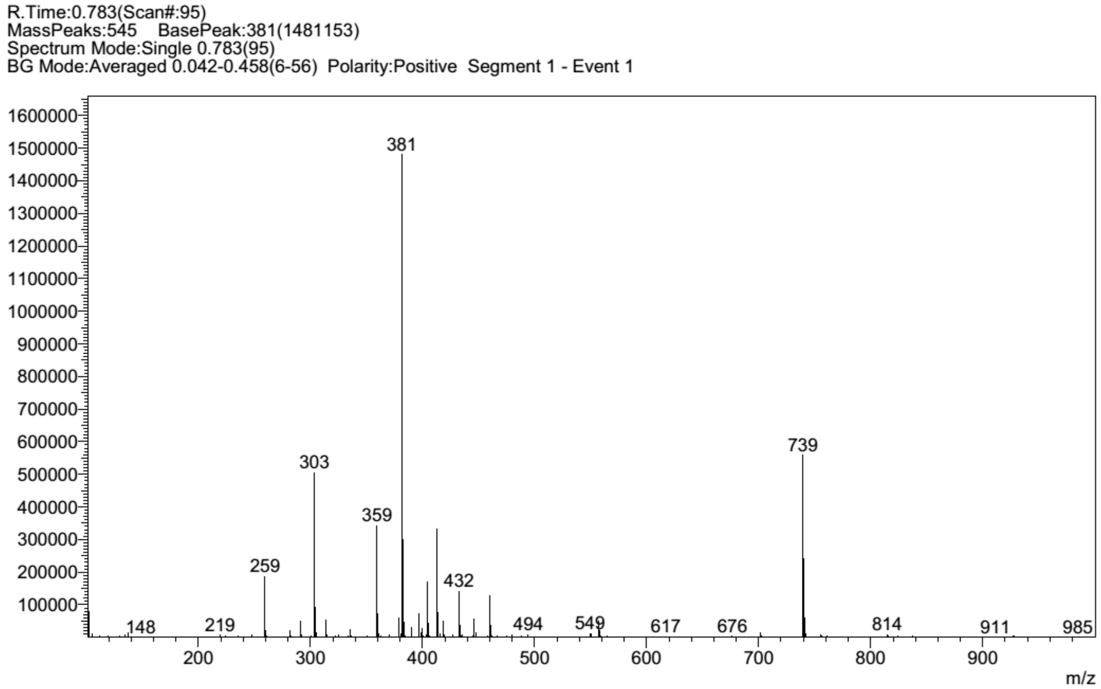


**Figure S39.** MS-ESI for compound **Leu-01**.

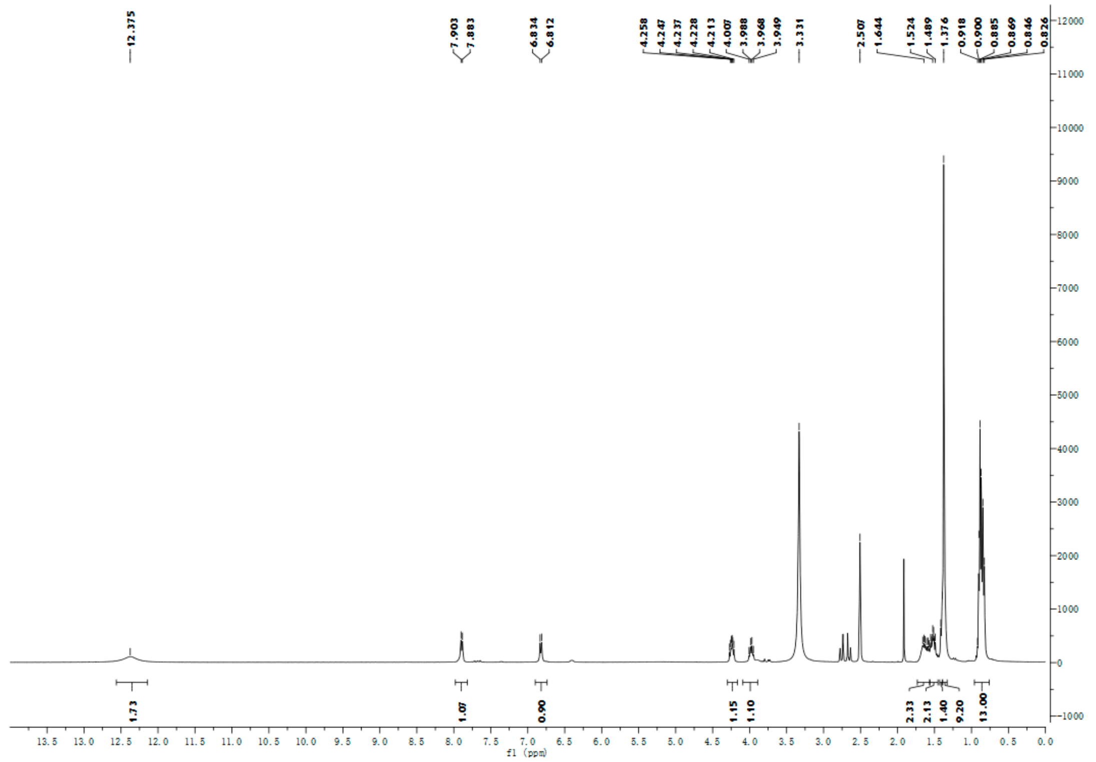

**Figure S40.** $^{1}$H-NMR for compound **Leu-02**.

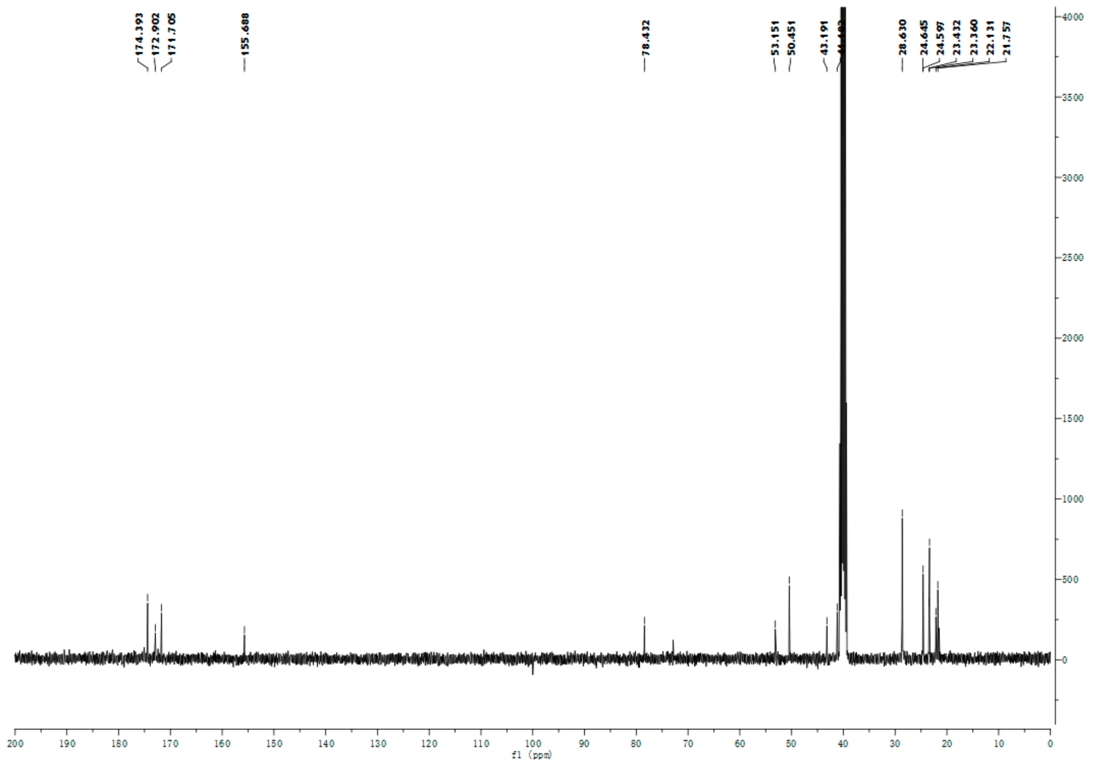

**Figure S41.** $^{13}$C-NMR for compound **Leu-02**.

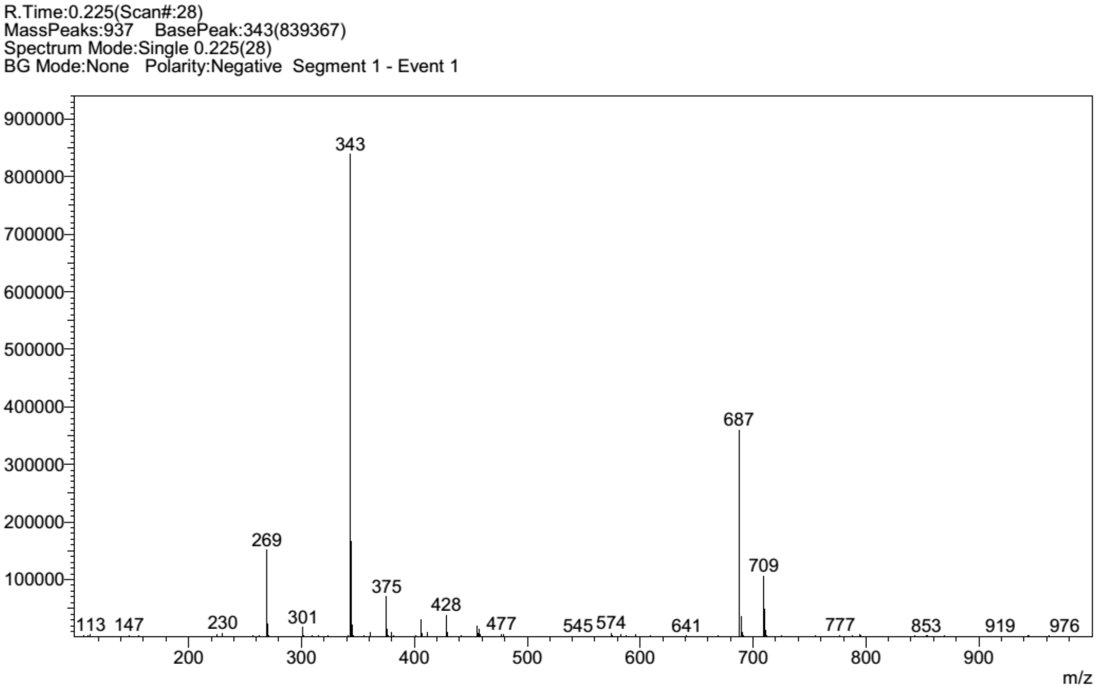


**Figure S42.** MS-ESI for compound **Leu-02**.

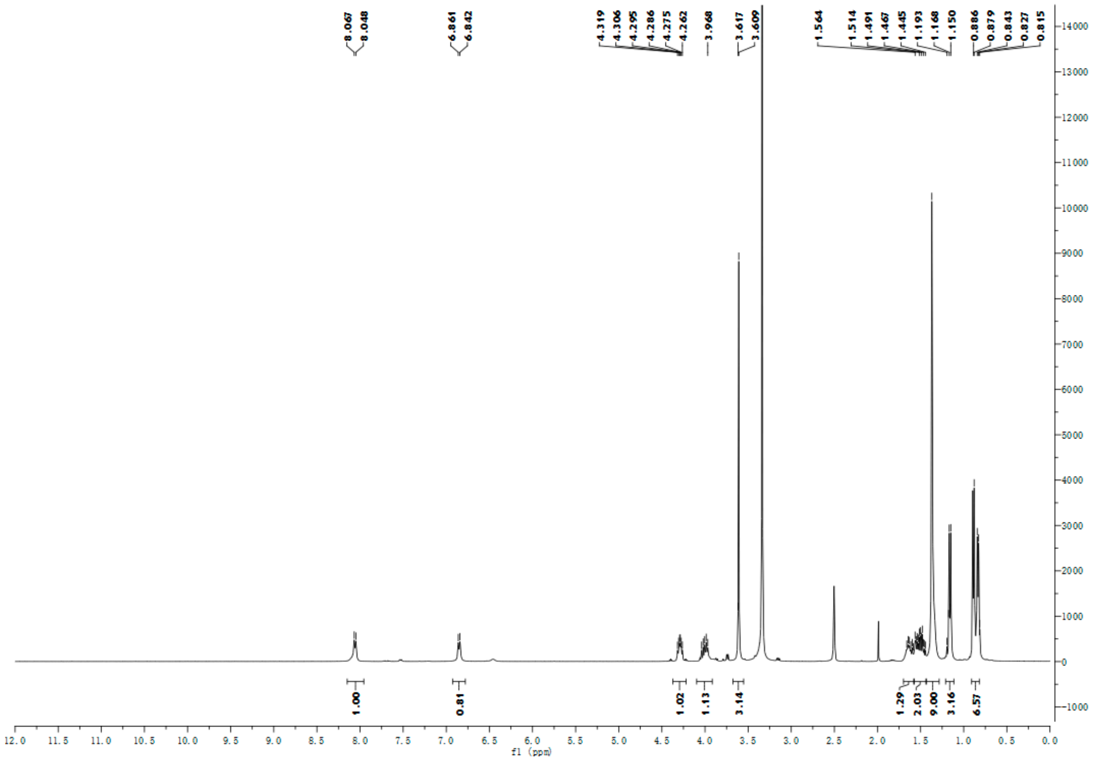


**Figure S43.** $^{1}$H-NMR for compound **Leu-03**.

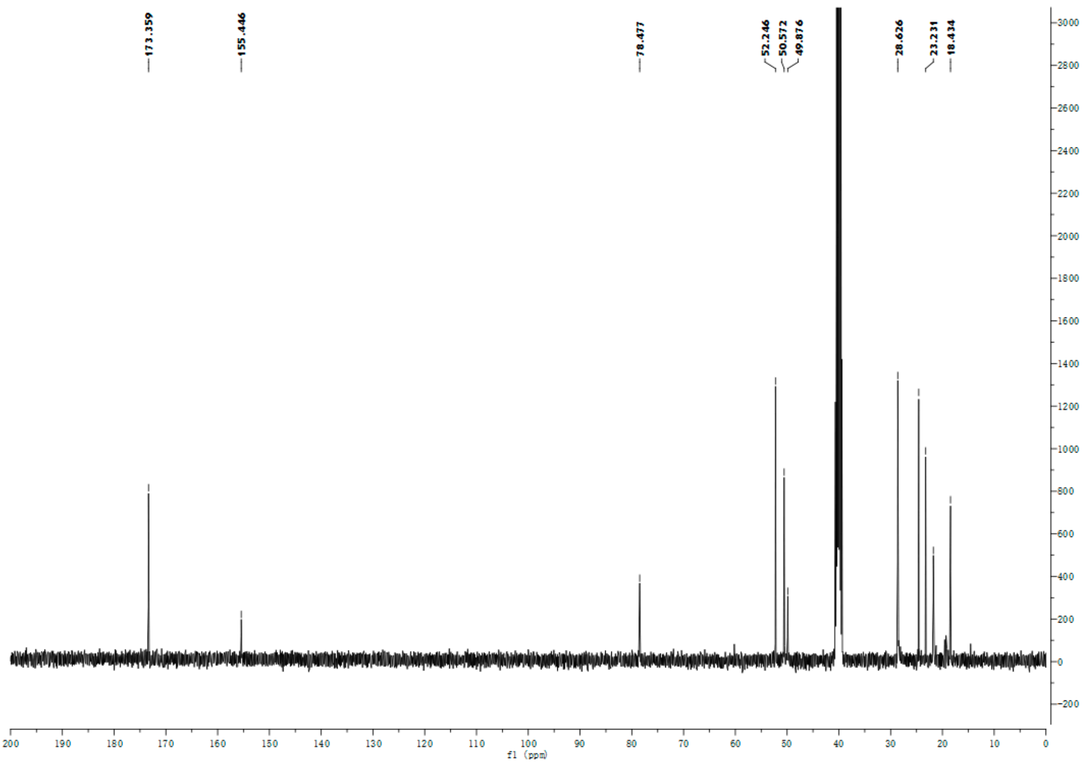


**Figure S44.** $^{13}C$-NMR for compound **Leu-03**.

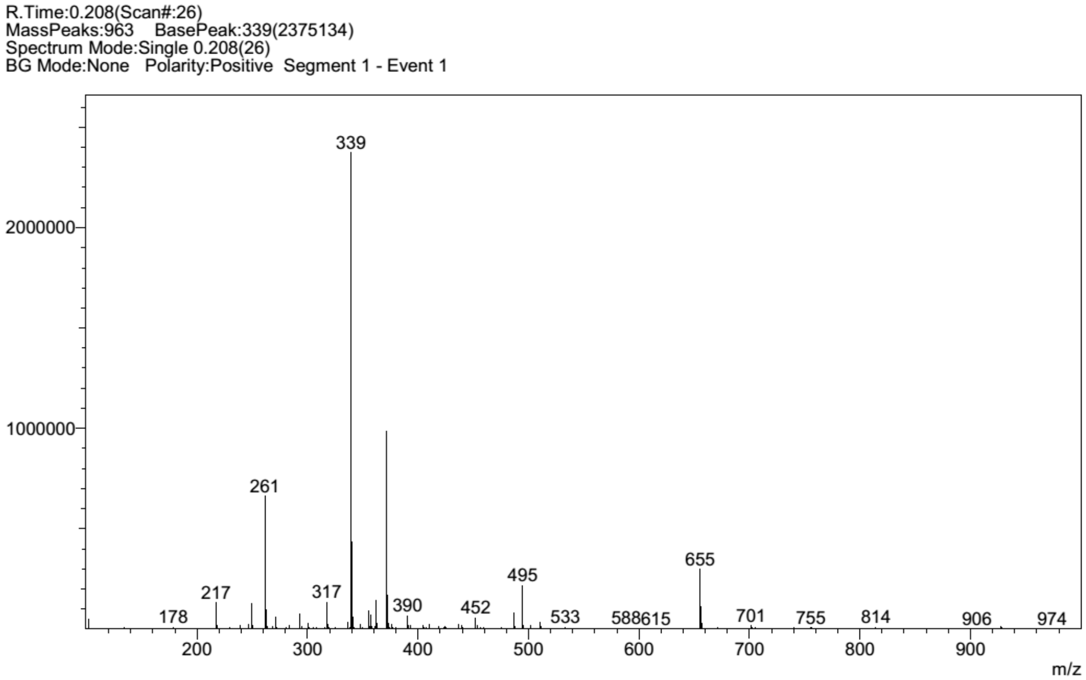


**Figure S45.** MS-ESI for compound **Leu-03**.

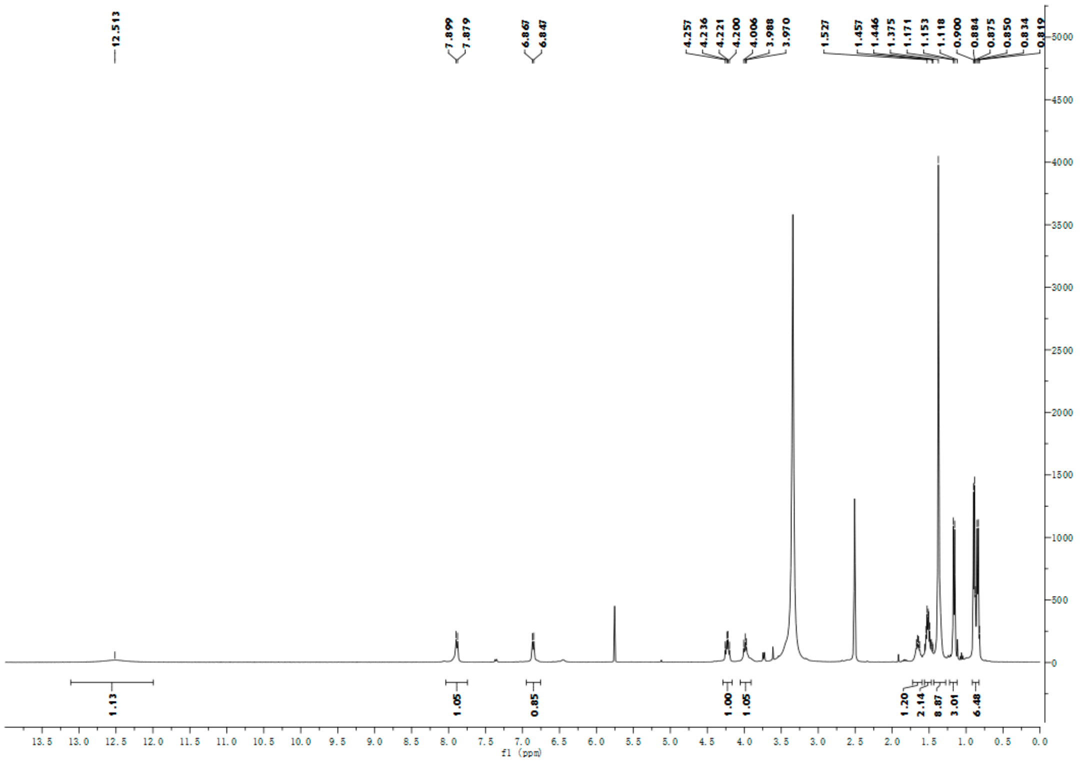

**Figure S46.** $^{1}$H-NMR for compound **Leu-04**.

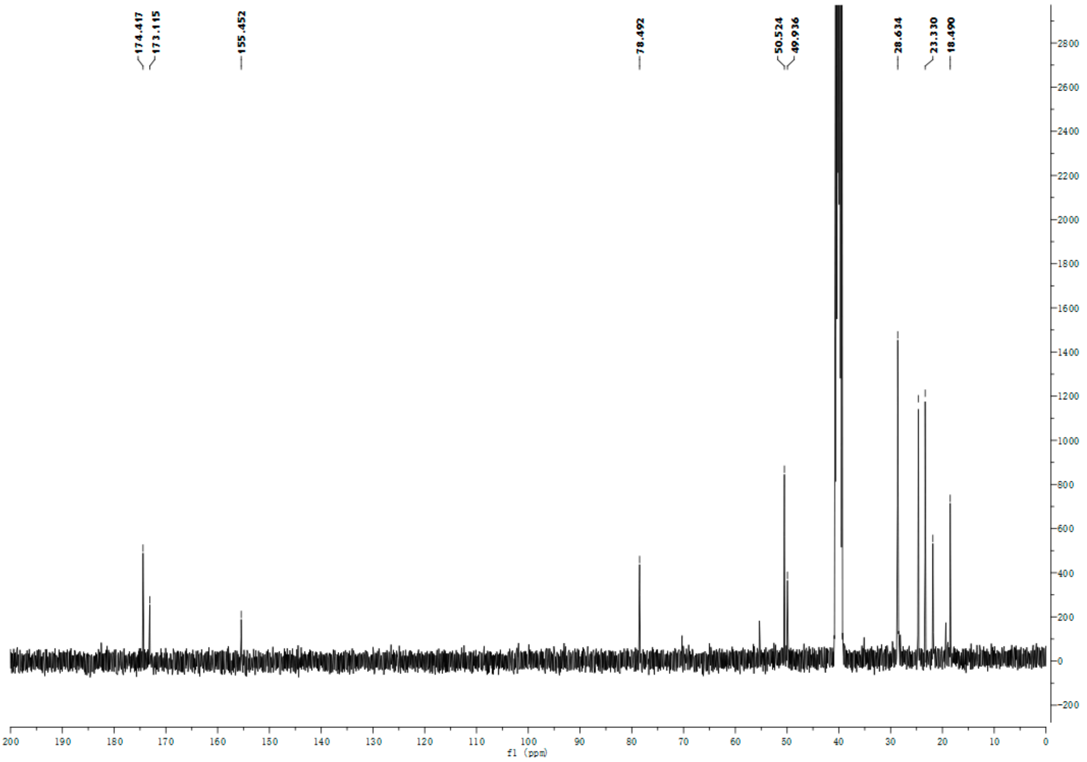

**Figure S47.** $^{13}$C-NMR for compound **Leu-04**.

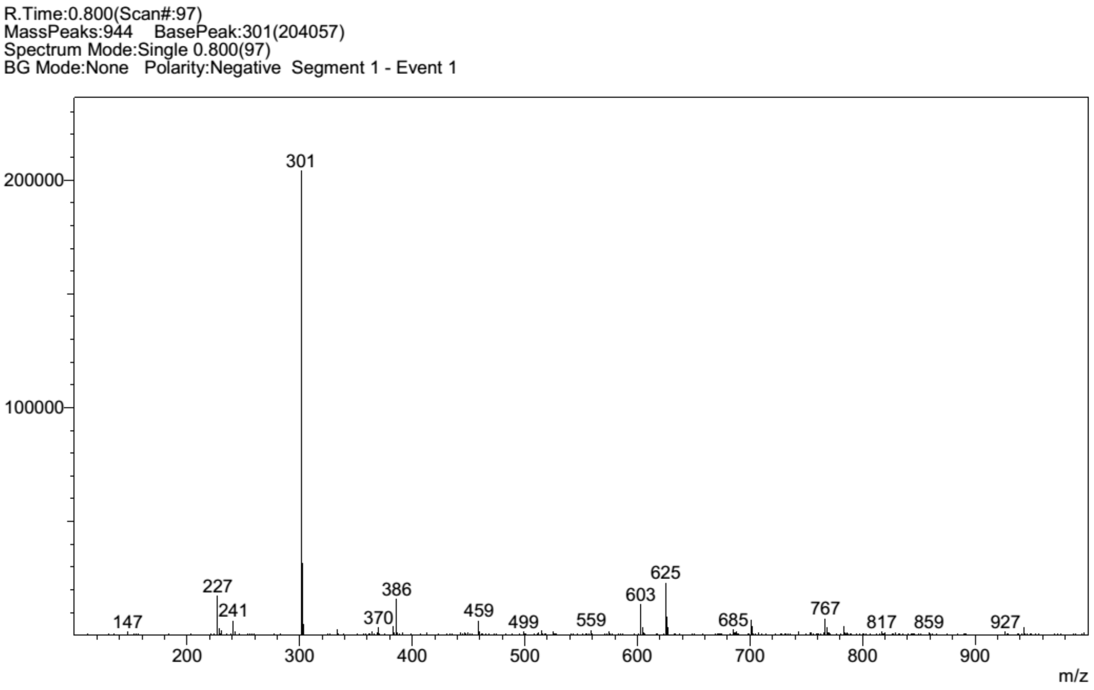


**Figure S48.** MS-ESI for compound **Leu-04**.

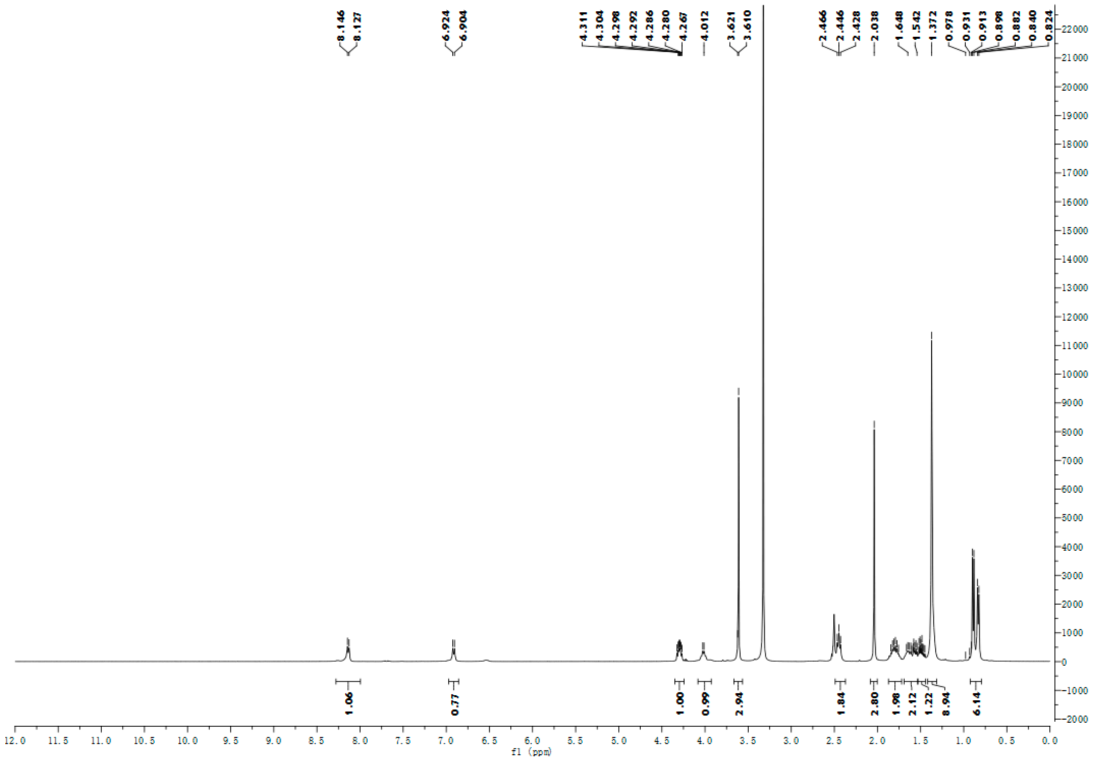


**Figure S49.** $^1$H-NMR for compound **Leu-05**.

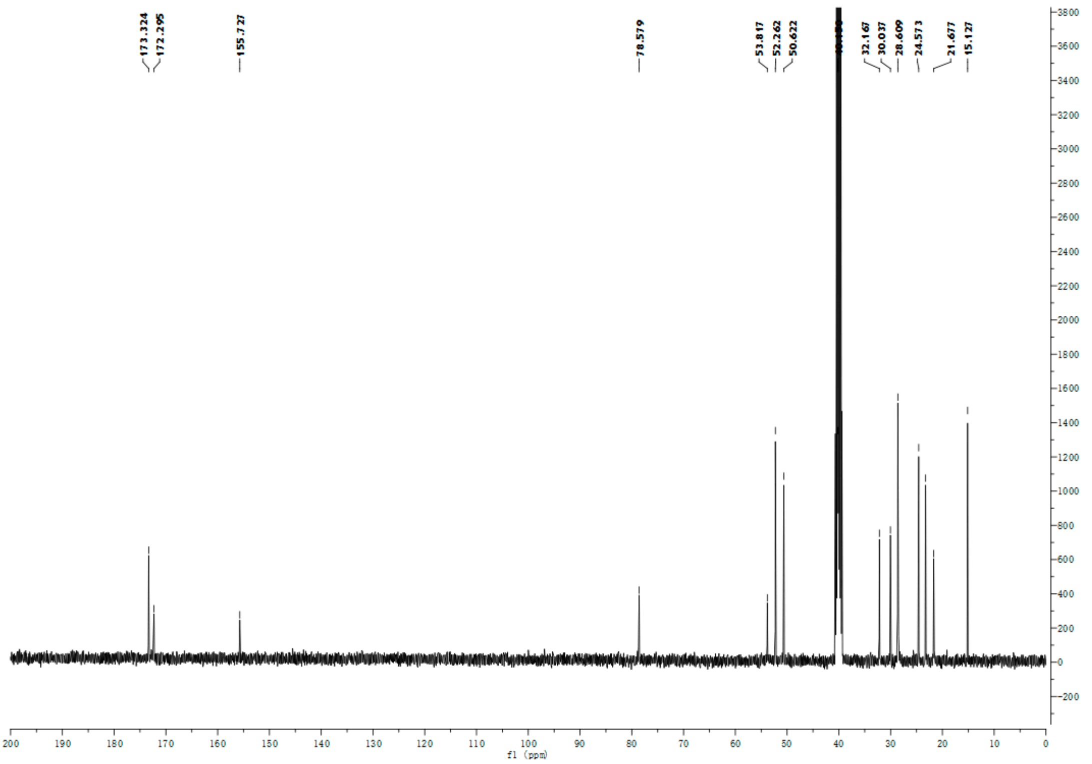


**Figure S50.** $^{13}$C-NMR for compound **Leu-05**.

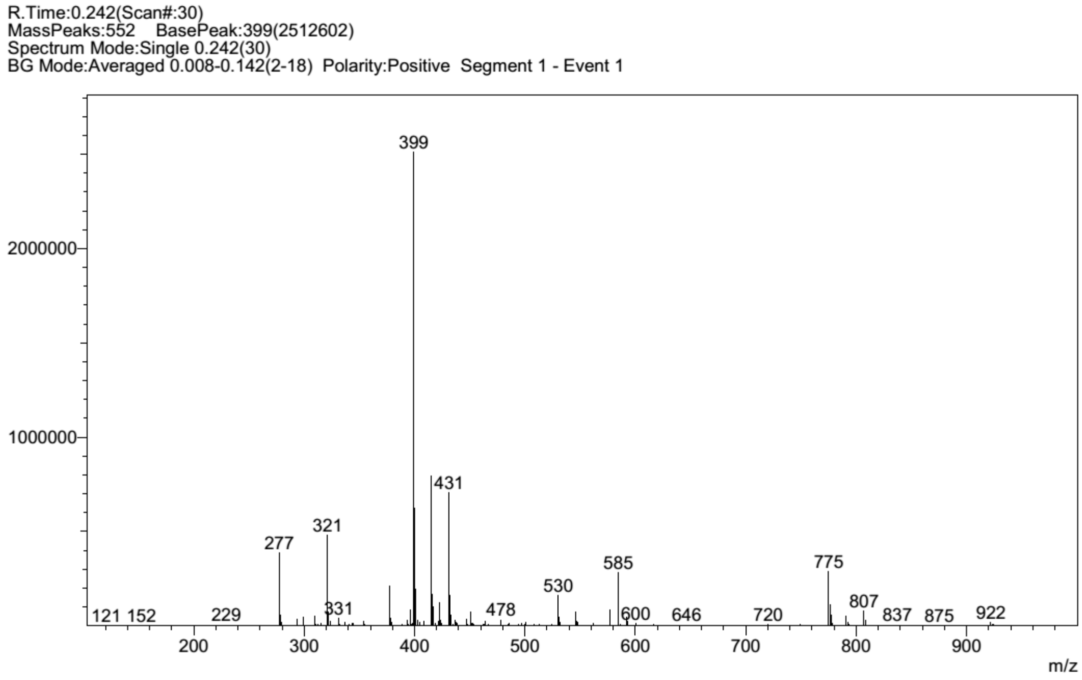


**Figure S51.** MS-ESI for compound **Leu-05**.

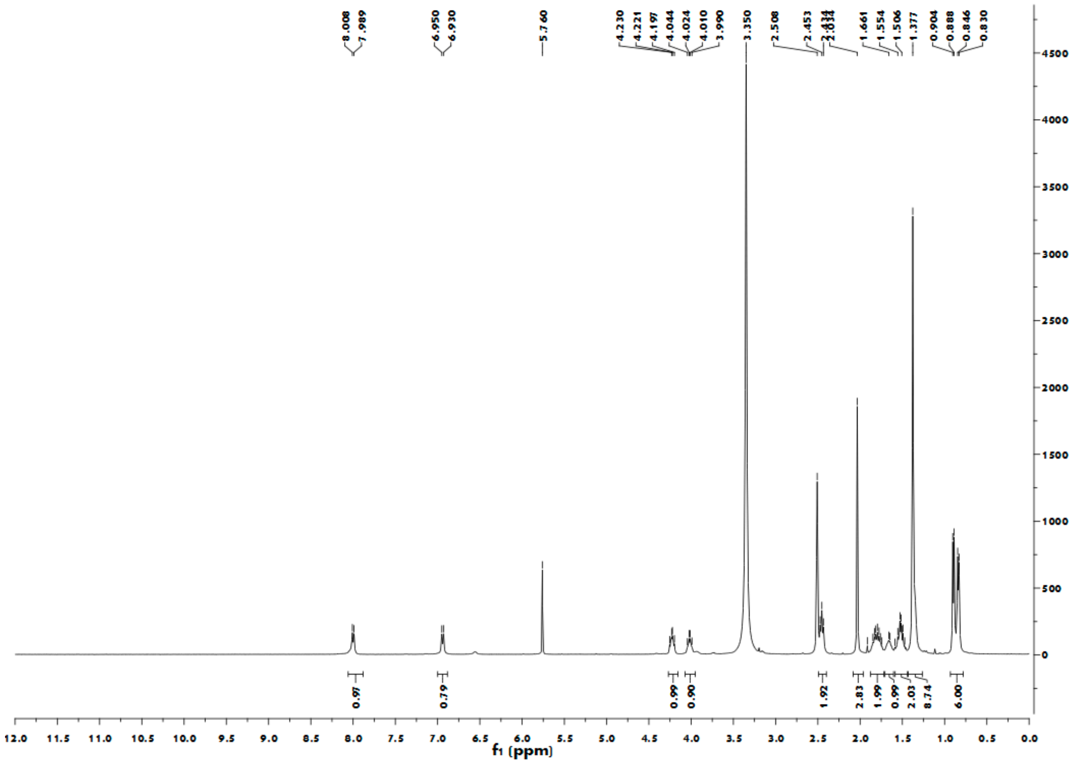


**Figure S52.** $^{1}$H-NMR for compound **Leu-06**.

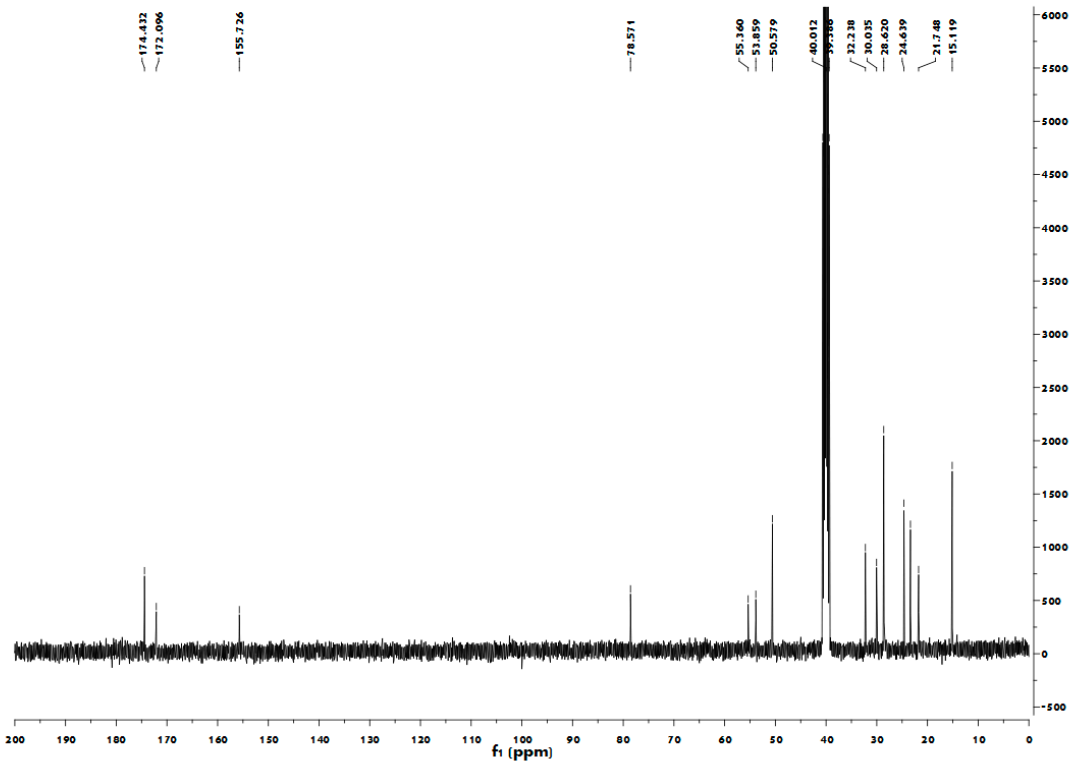


**Figure S53.** $^{13}$C-NMR for compound **Leu-06**.

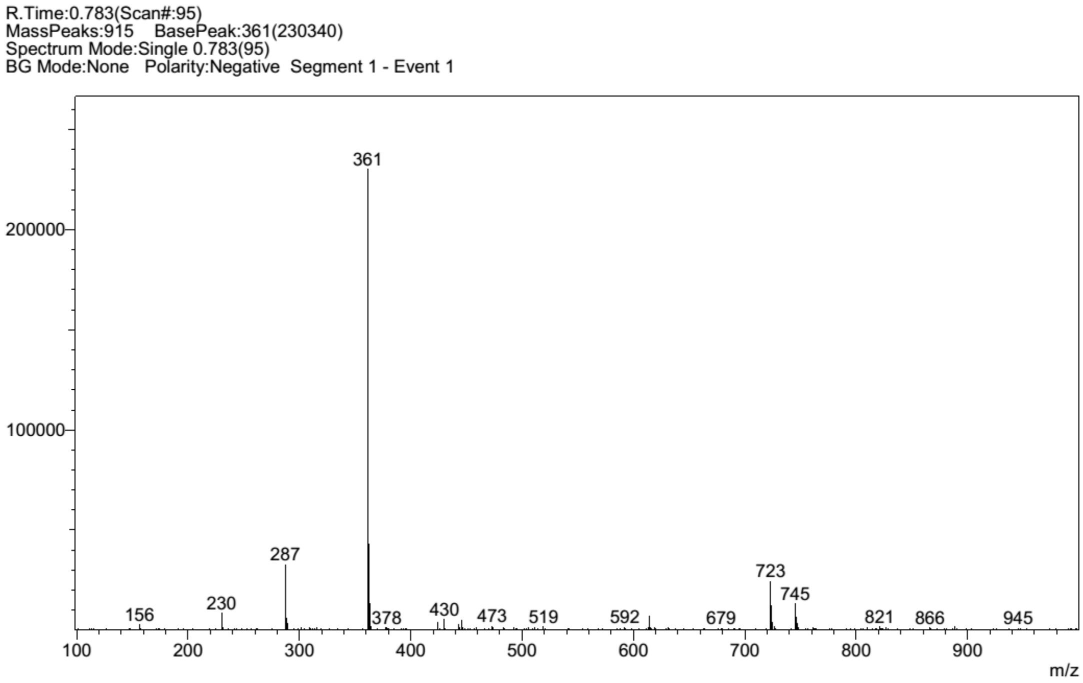


**Figure S54.** MS-ESI for compound **Leu-06**.

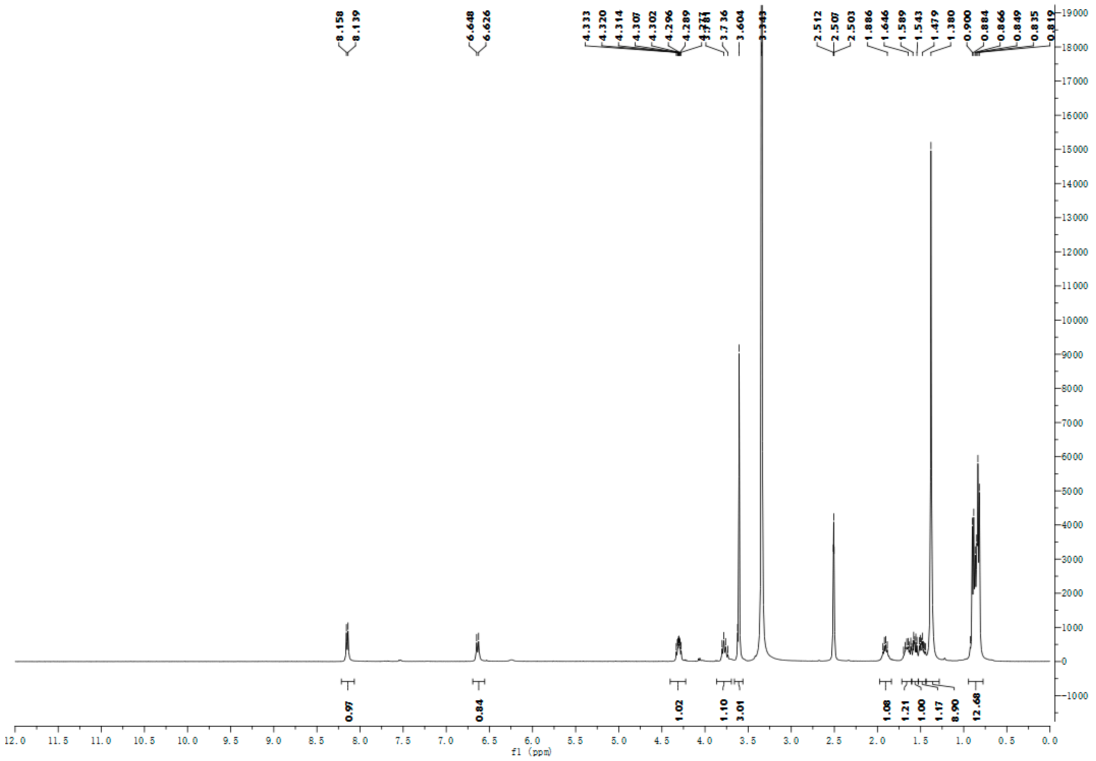


**Figure S55.** $^{1}$H-NMR for compound **Leu-07**.

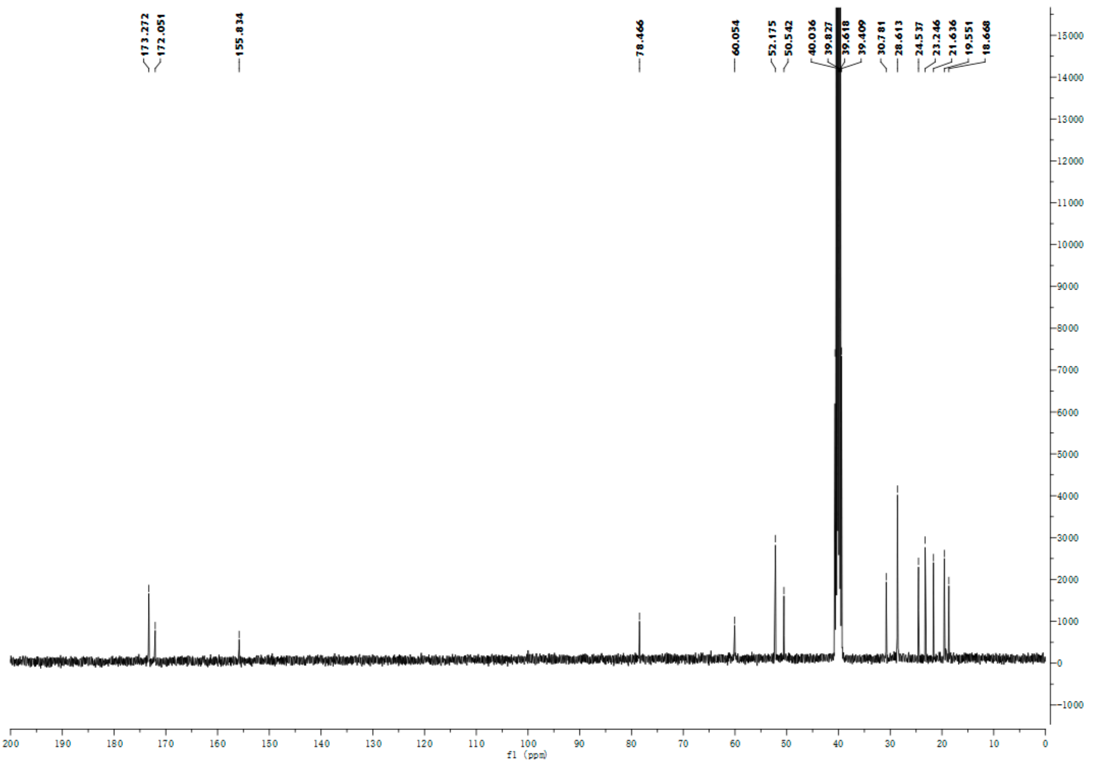


**Figure S56.** $^{13}$C-NMR for compound **Leu-07**.

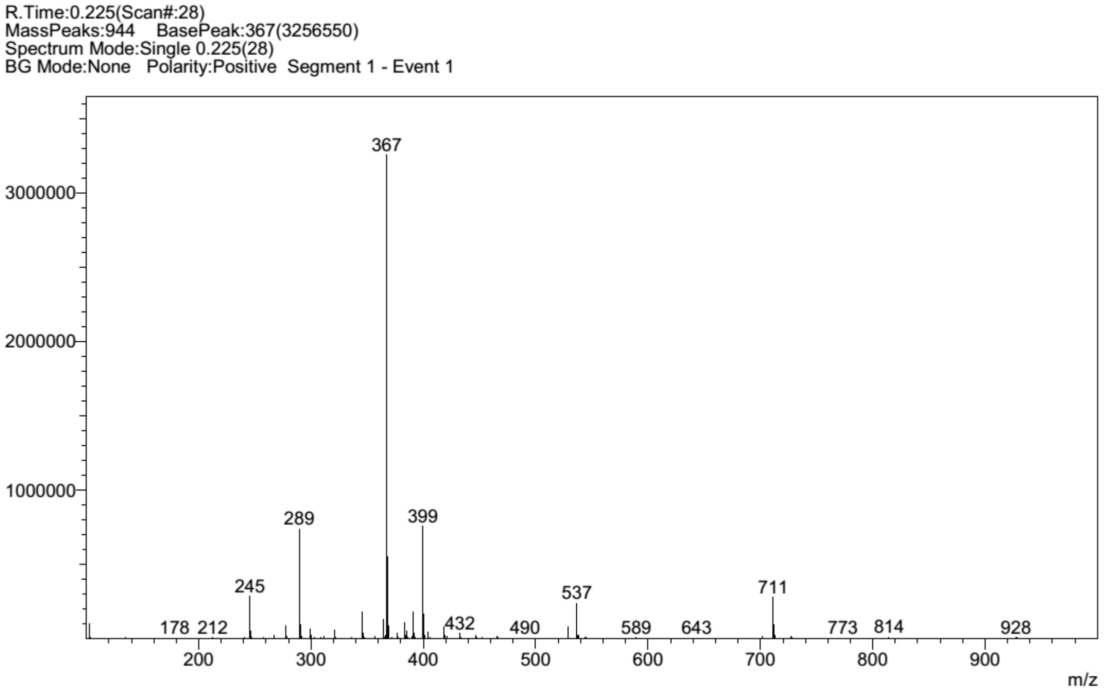


**Figure S57.** MS-ESI for compound **Leu-07**.

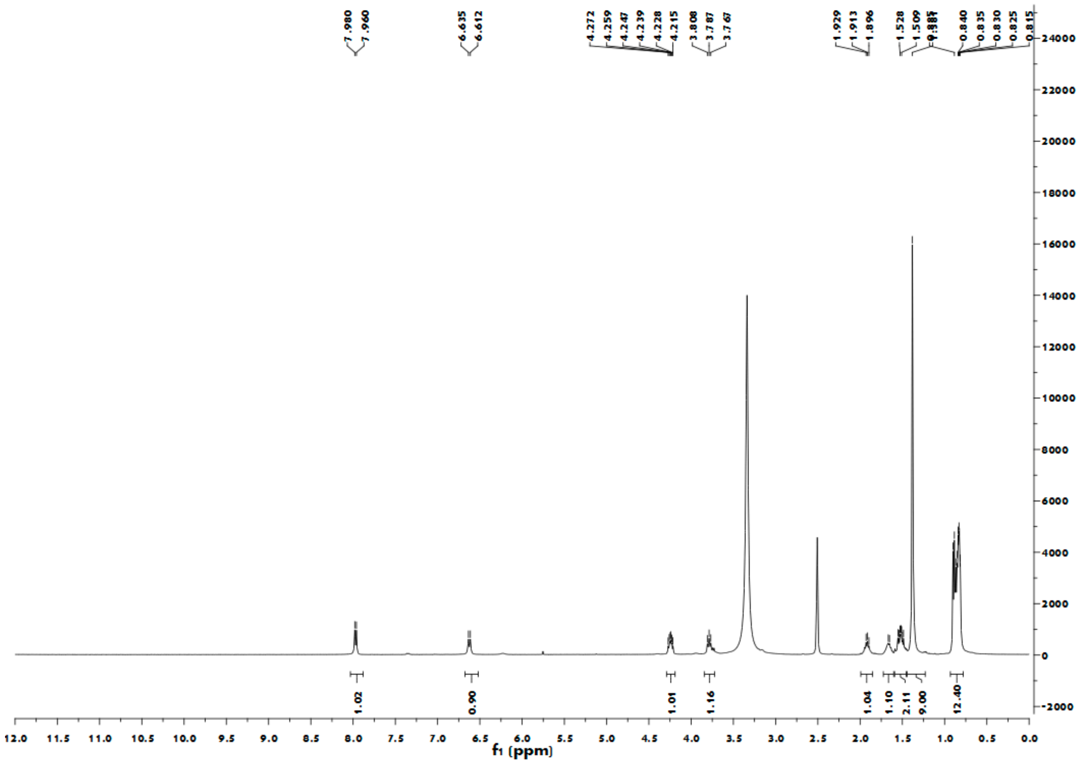


**Figure S58.** $^{1}$H-NMR for compound **Leu-08**.

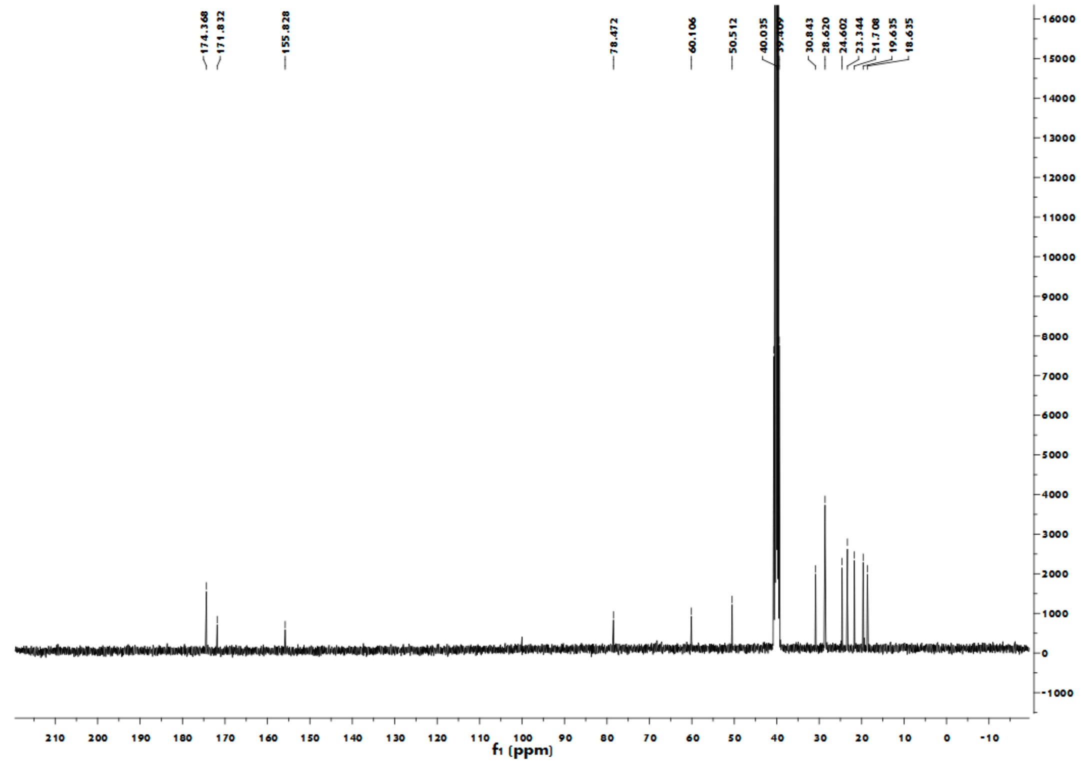


**Figure S59.** $^{13}$C-NMR for compound **Leu-08**.

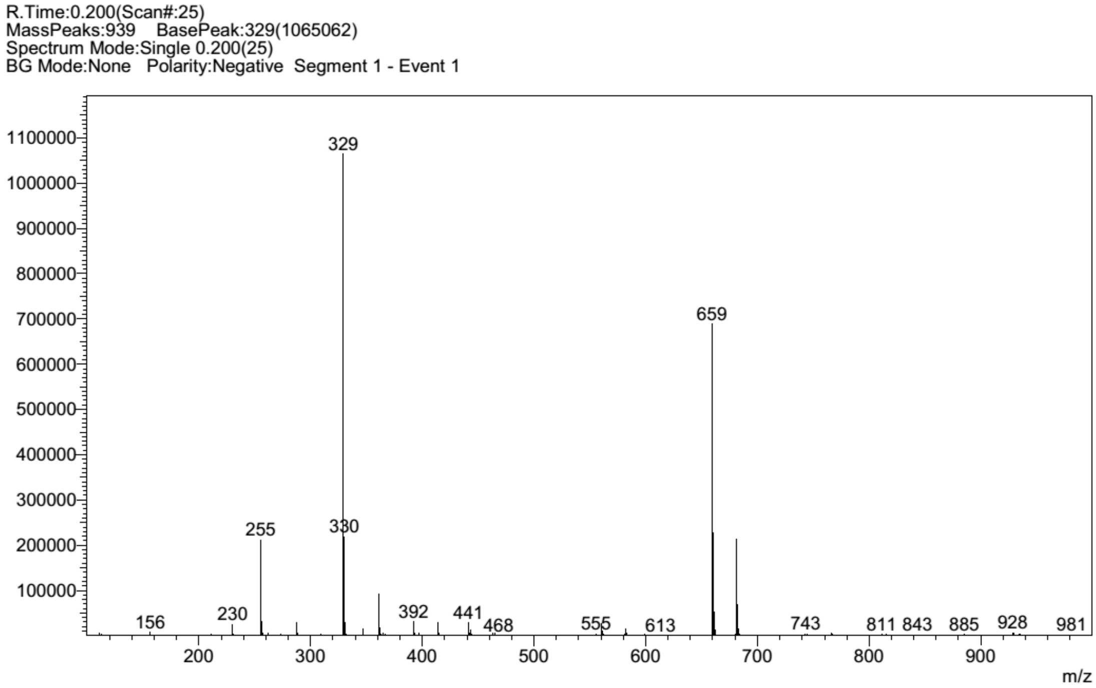


**Figure S60.** MS-ESI for compound **Leu-08**.

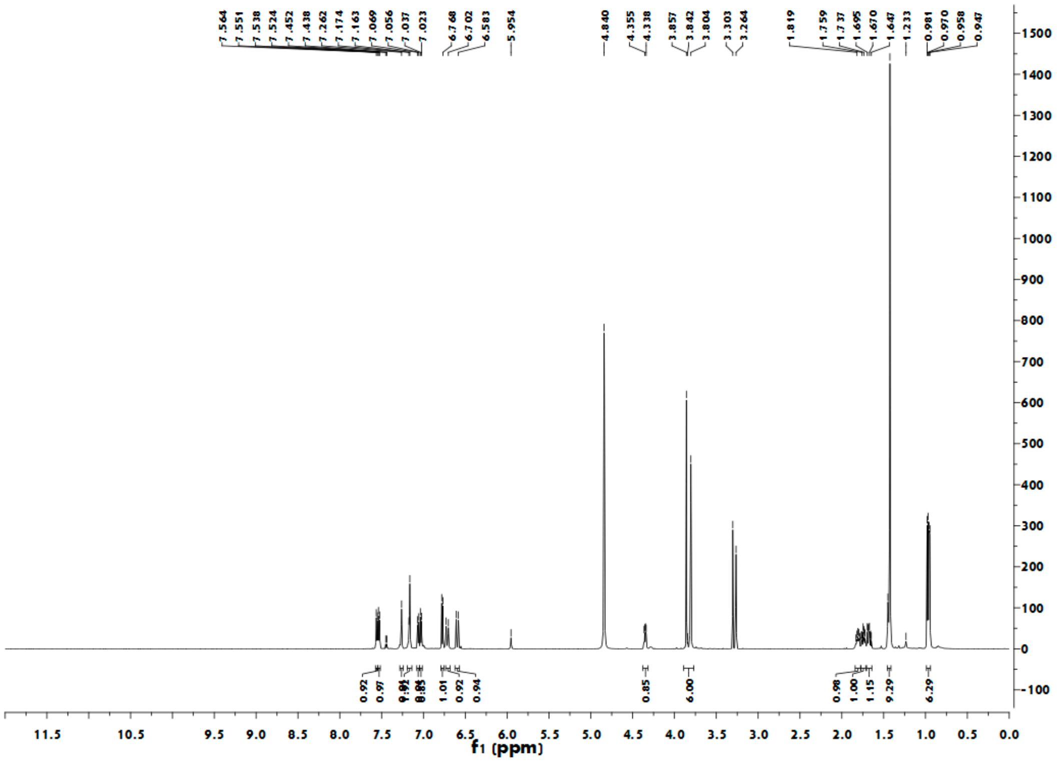


**Figure S61.** $^{1}$H-NMR for compound **9**.

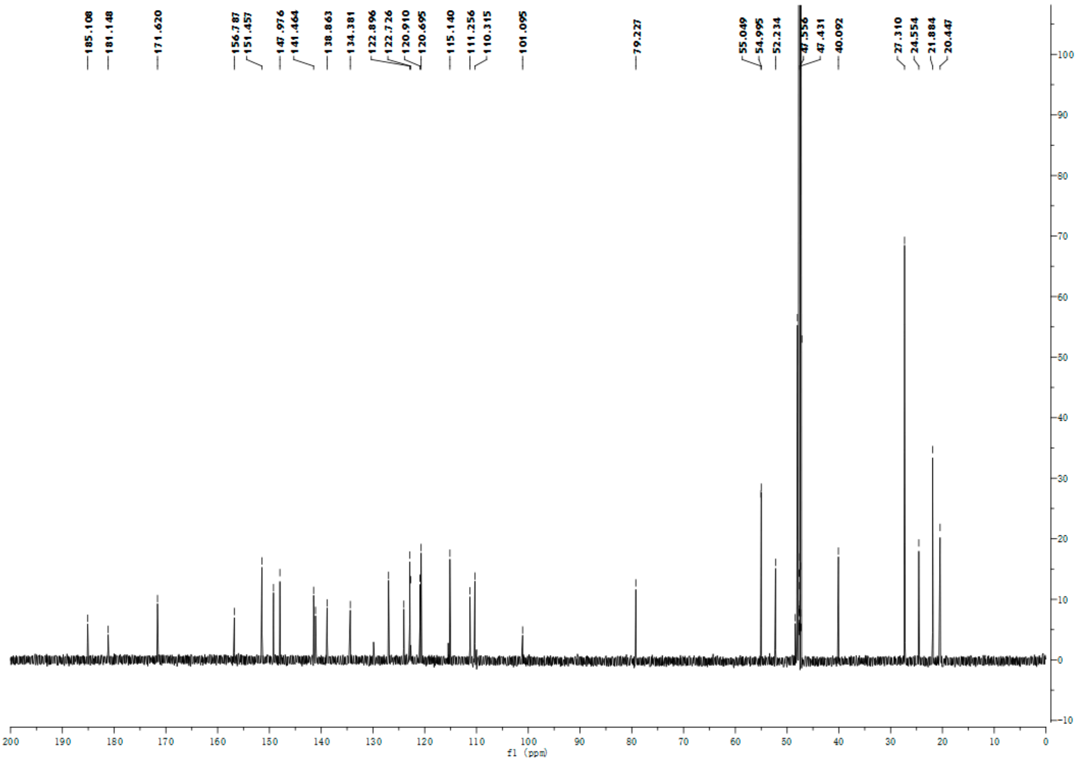


**Figure S62.** $^{13}$C-NMR for compound **9**.

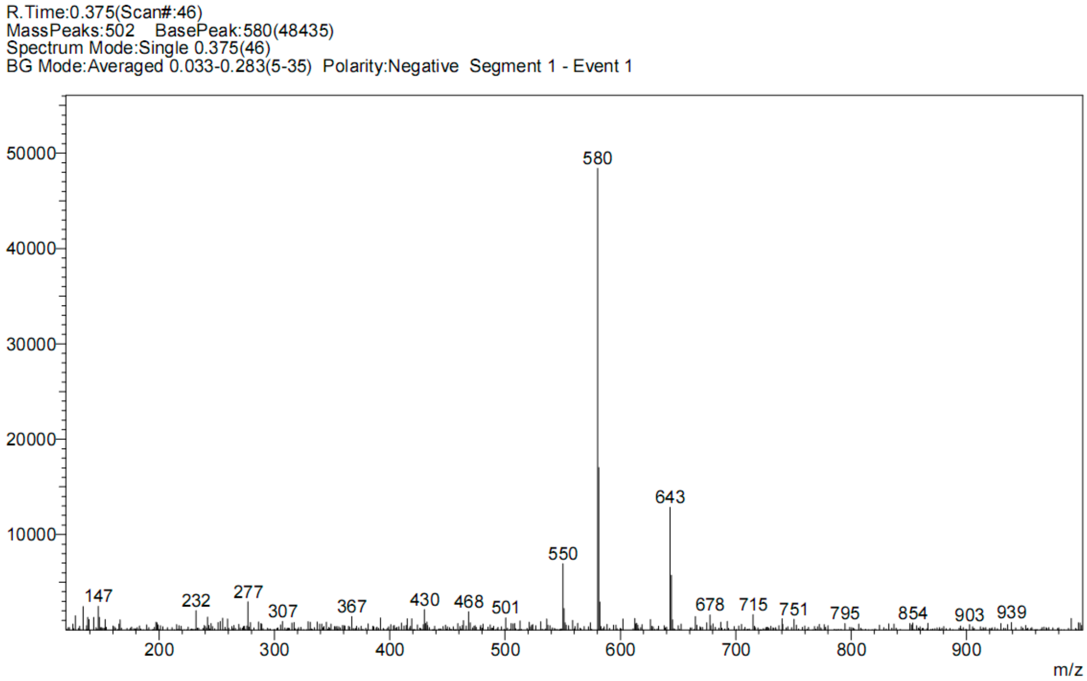


**Figure S63.** MS-ESI for compound **9**.

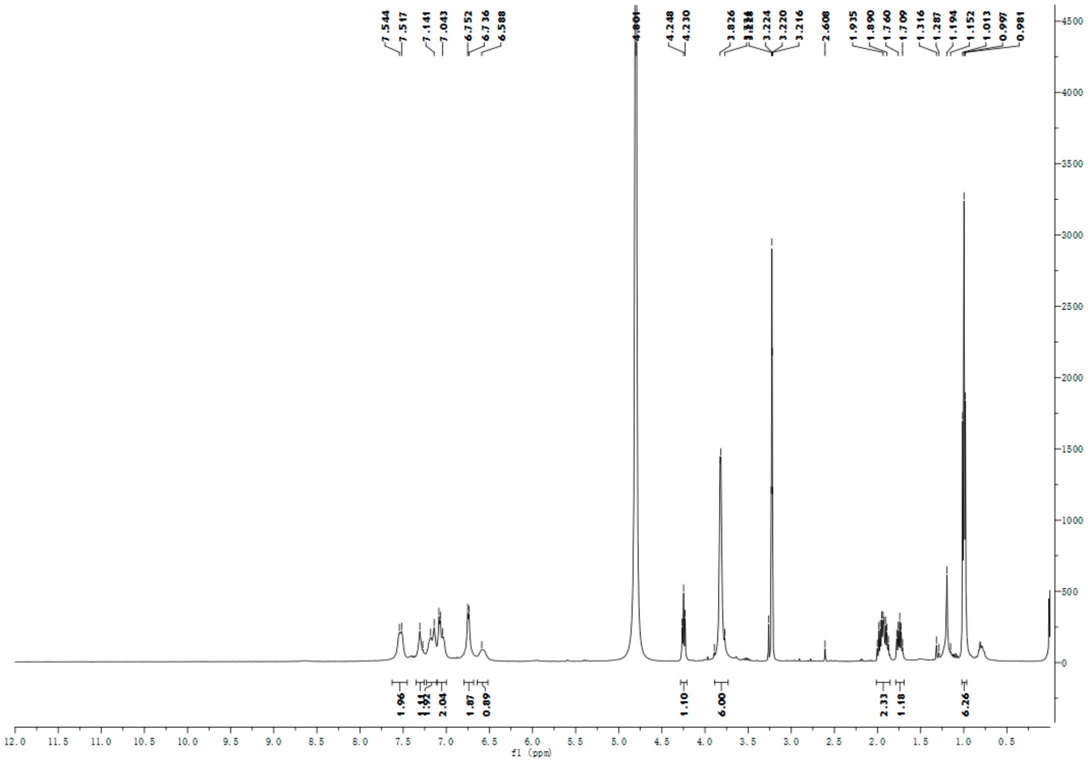


**Figure S64.** $^{1}$H-NMR for compound **10**.

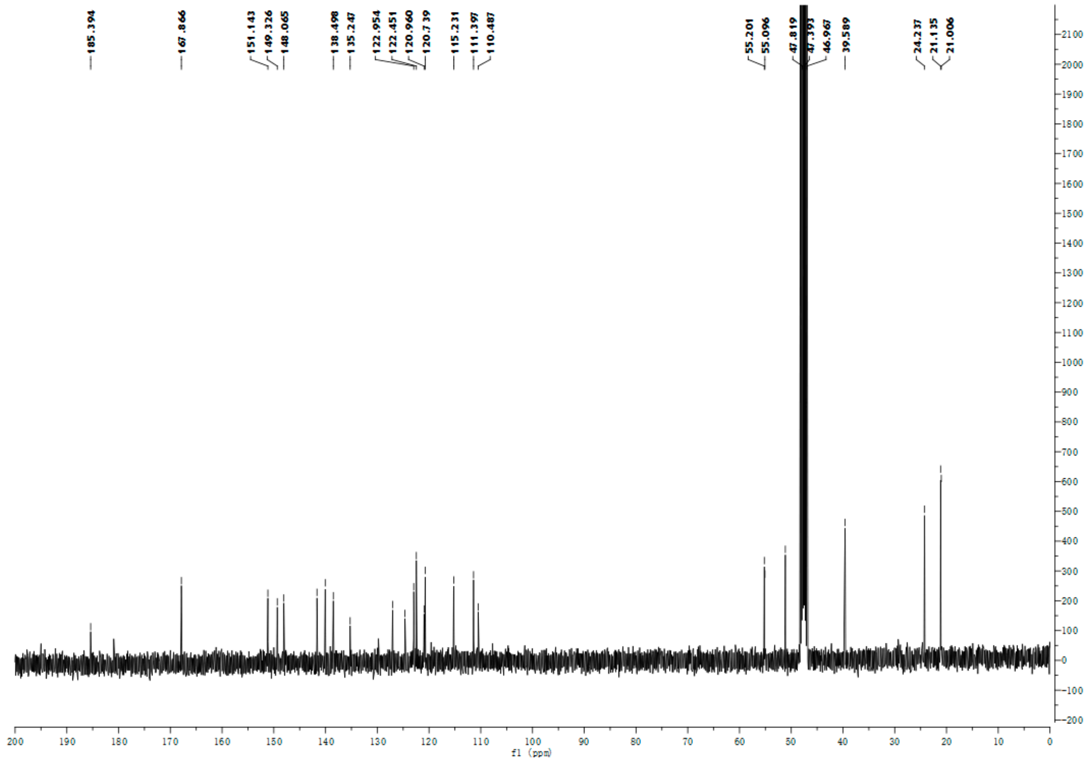


**Figure S65.** $^{13}$C-NMR for compound **10**.

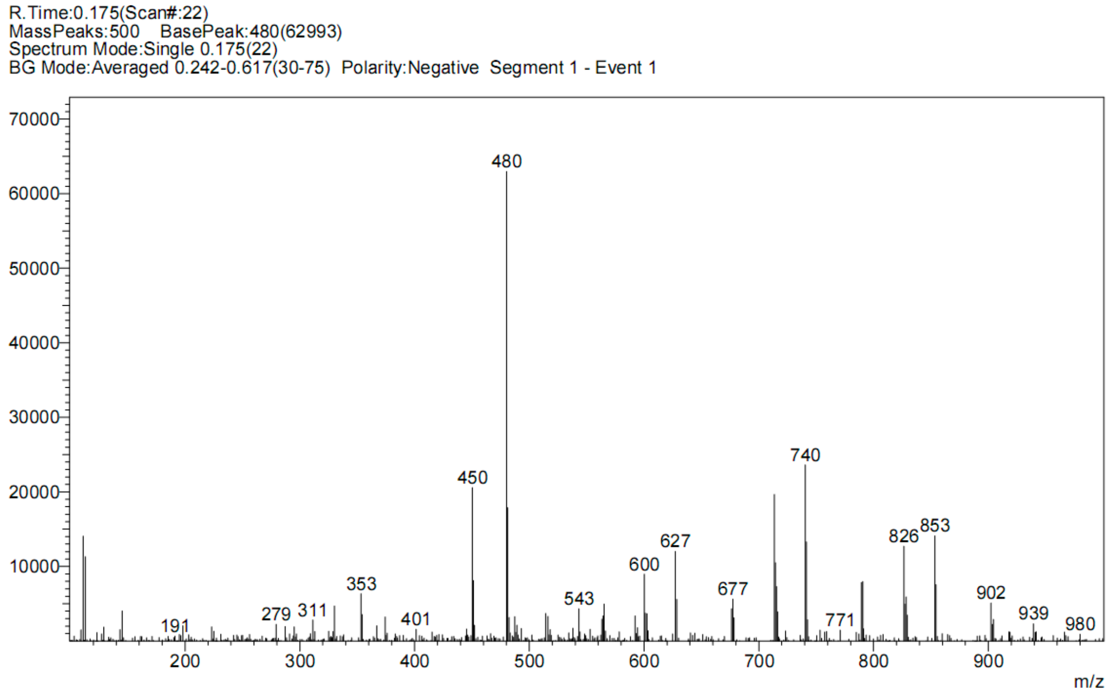


**Figure S66.** MS-ESI for compound **10**.

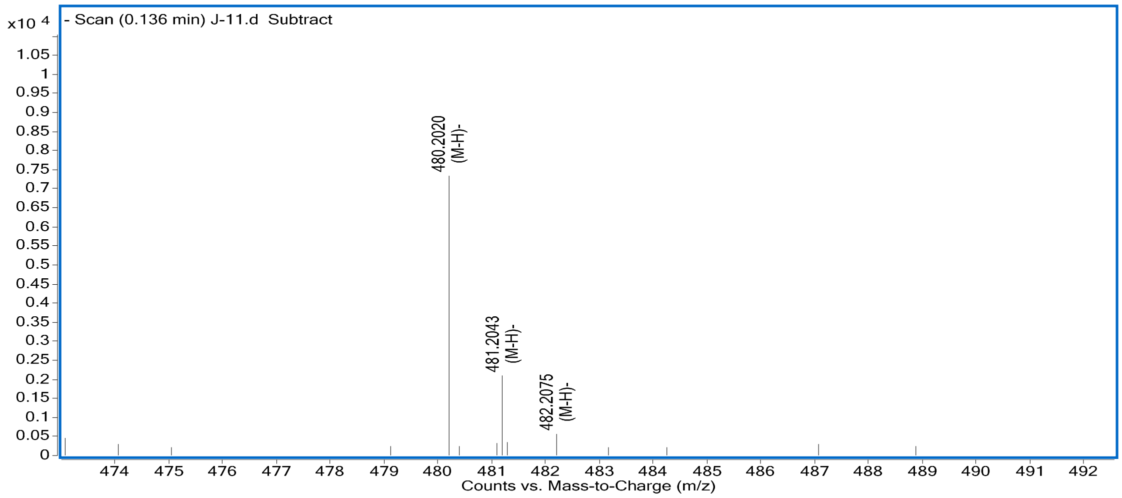


**Figure S67.** HRMS-ESI for compound **10**.

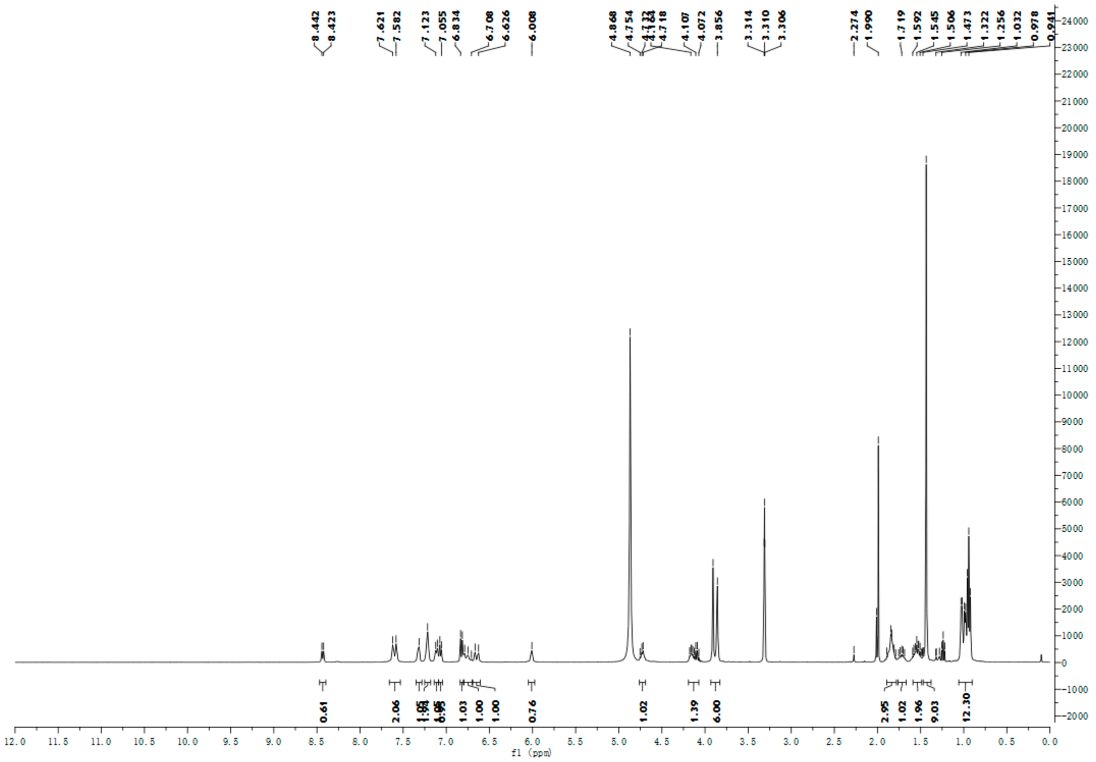


**Figure S68.** $^{1}$H-NMR for compound **11**.

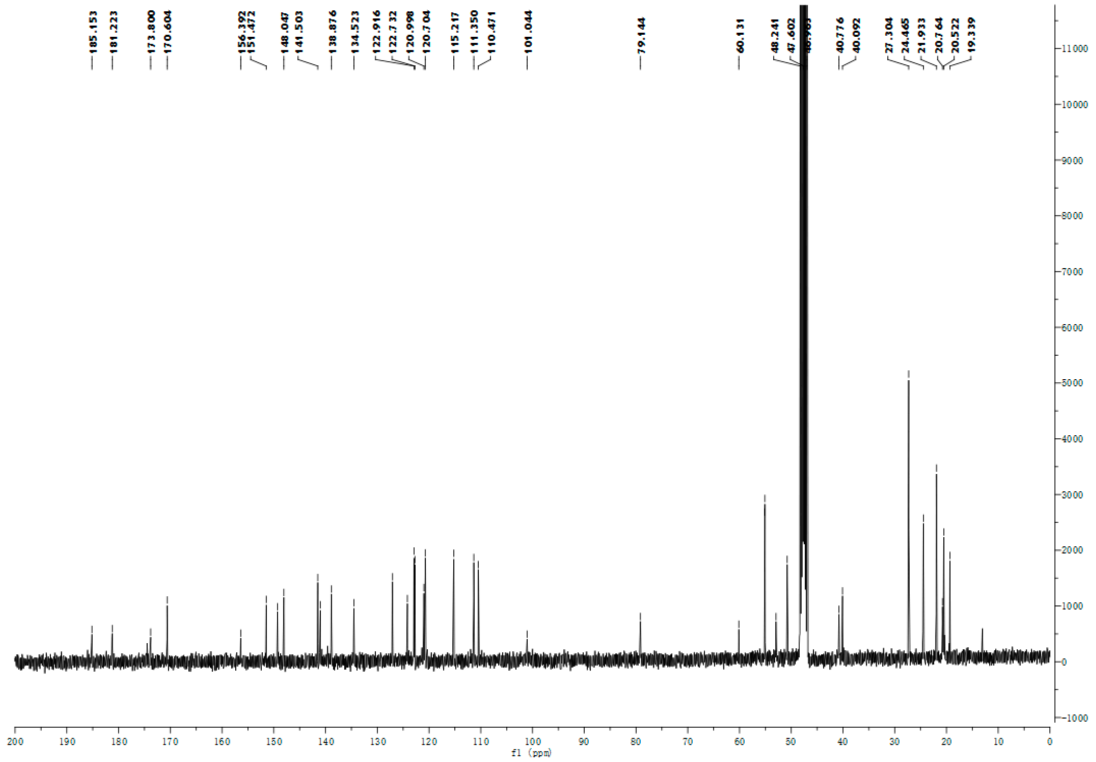


**Figure S69.** $^{13}$C-NMR for compound **11**.

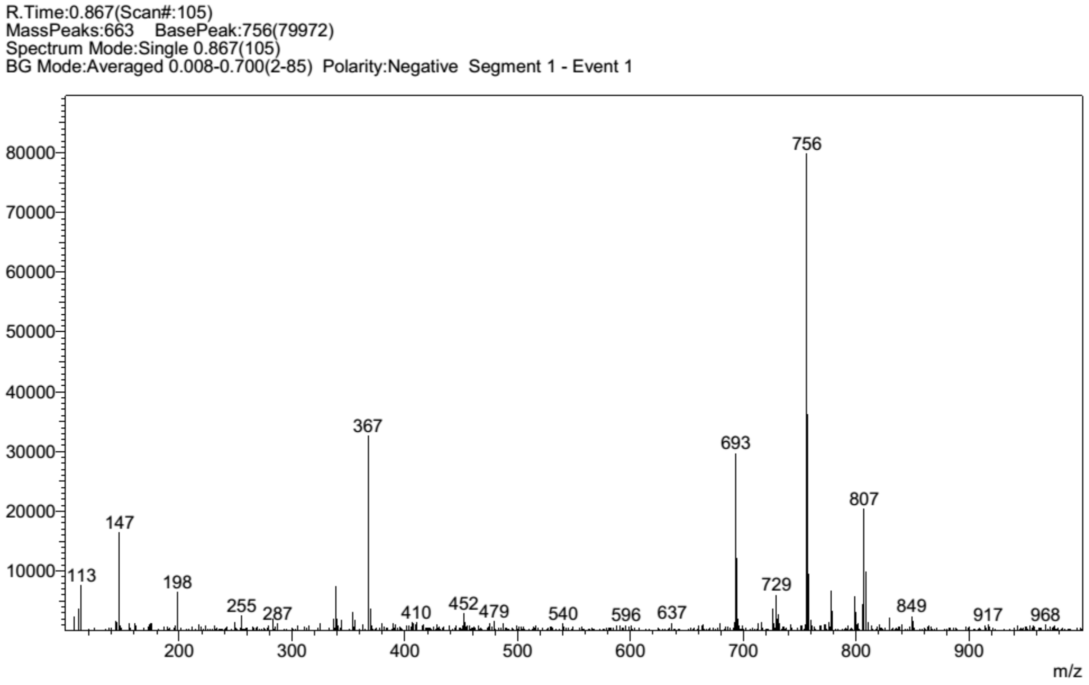


**Figure S70.** MS-ESI for compound **11**.

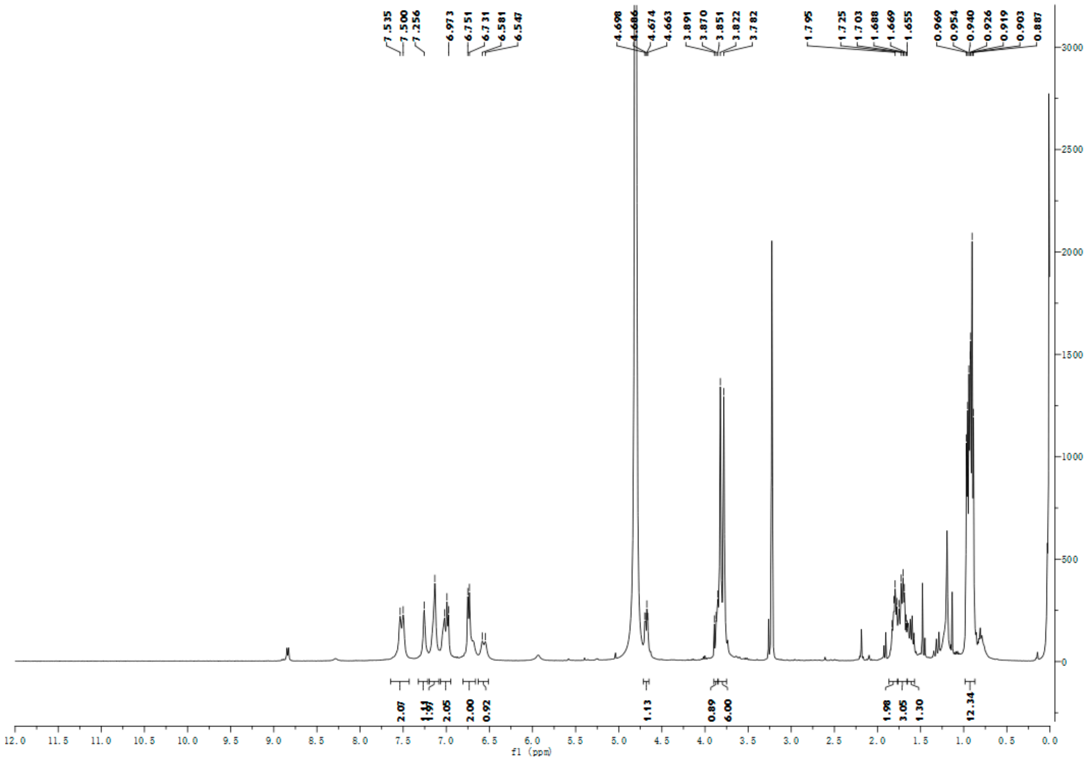


**Figure S71.** $^{1}$H-NMR for compound **12**.

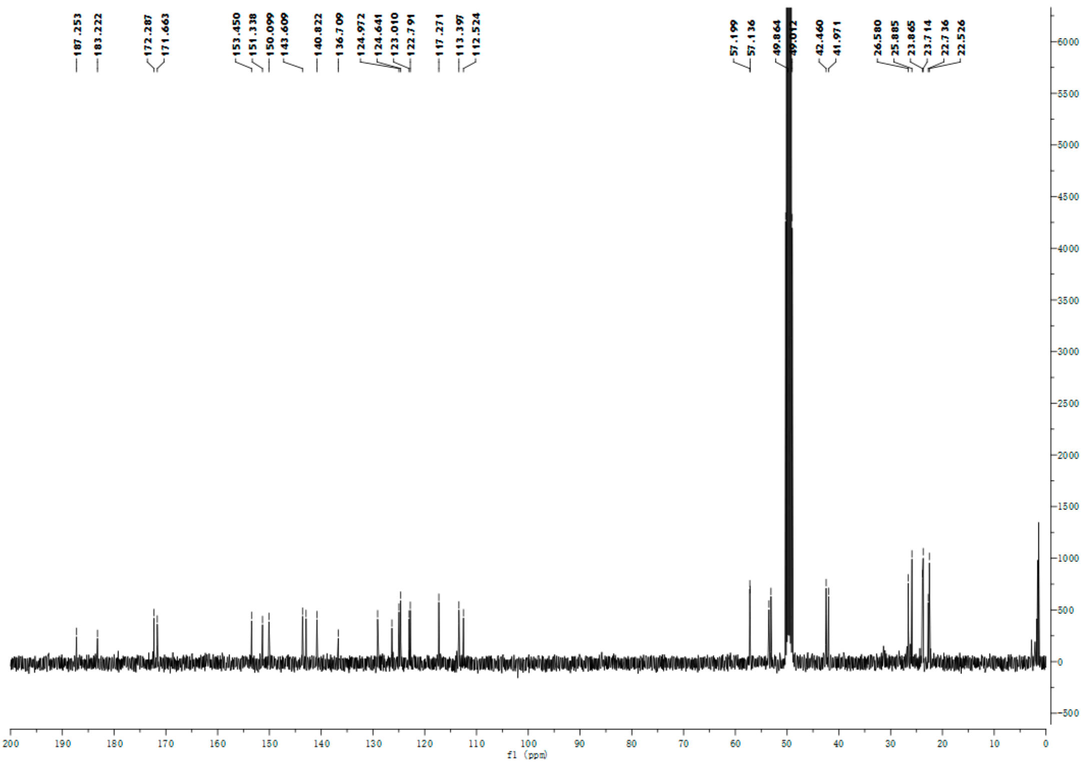


**Figure S72.** $^{13}$C-NMR for compound **12**.

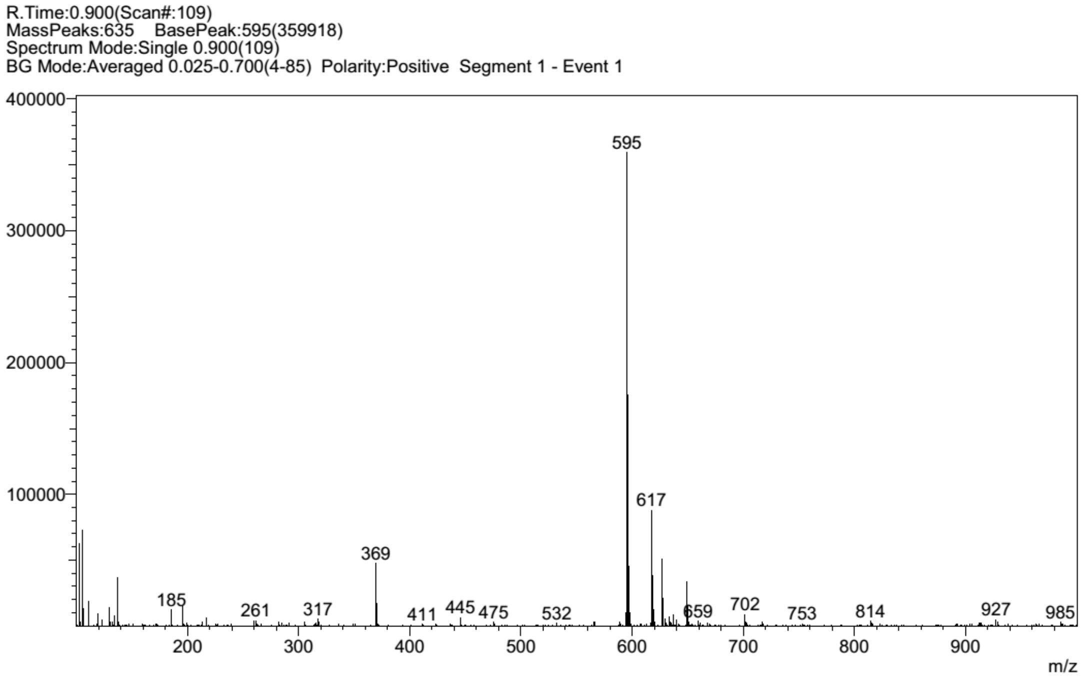


**Figure S73.** MS-ESI for compound **12**.

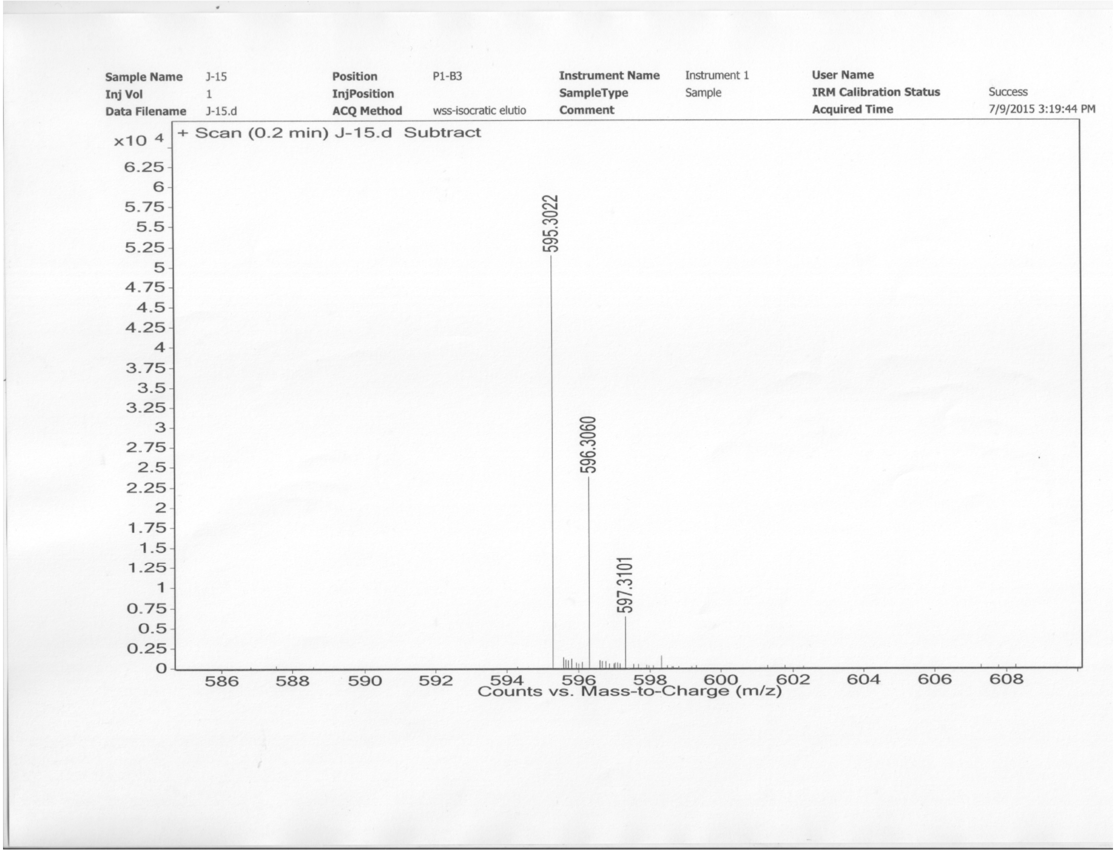


**Figure S74.** HRMS-ESI for compound **12**.

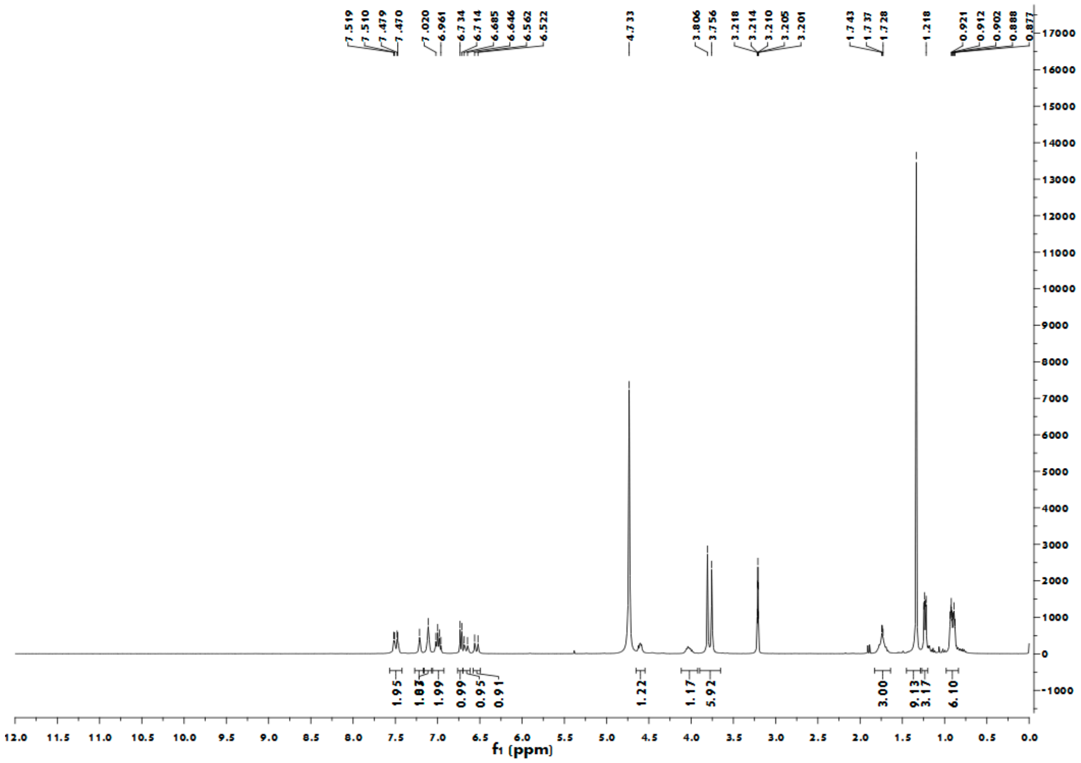


**Figure S75.** $^{1}$H-NMR for compound **13**.

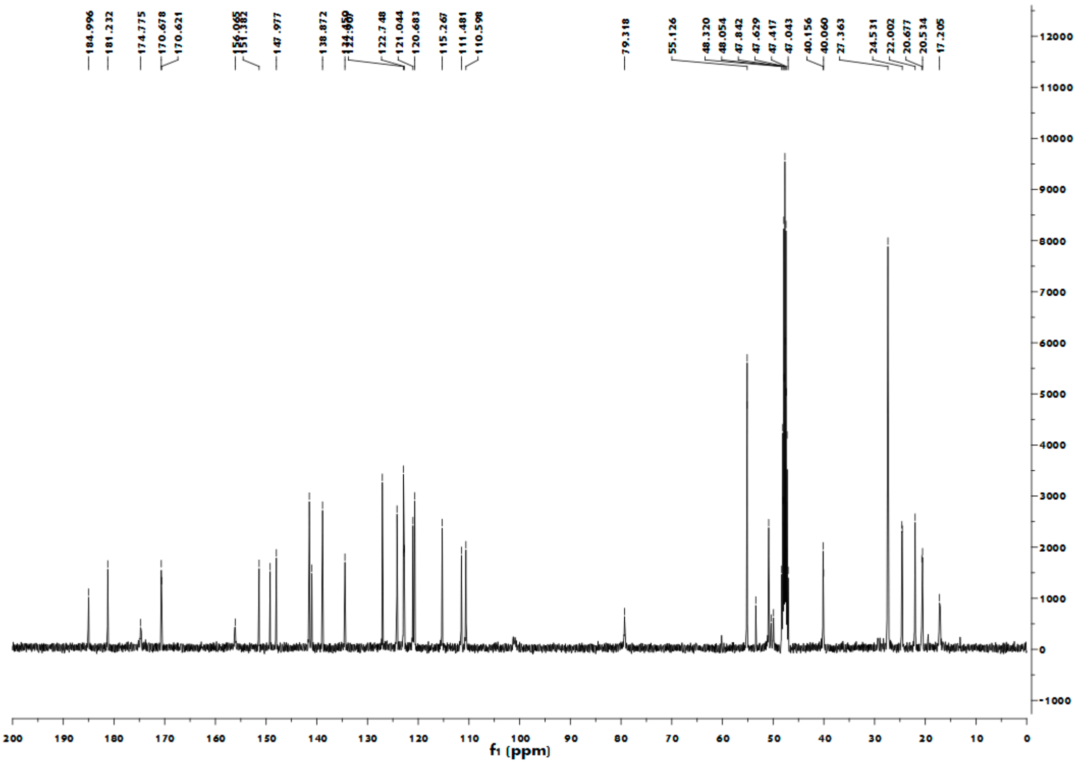

**Figure S76.** $^{13}$C-NMR for compound **13**.

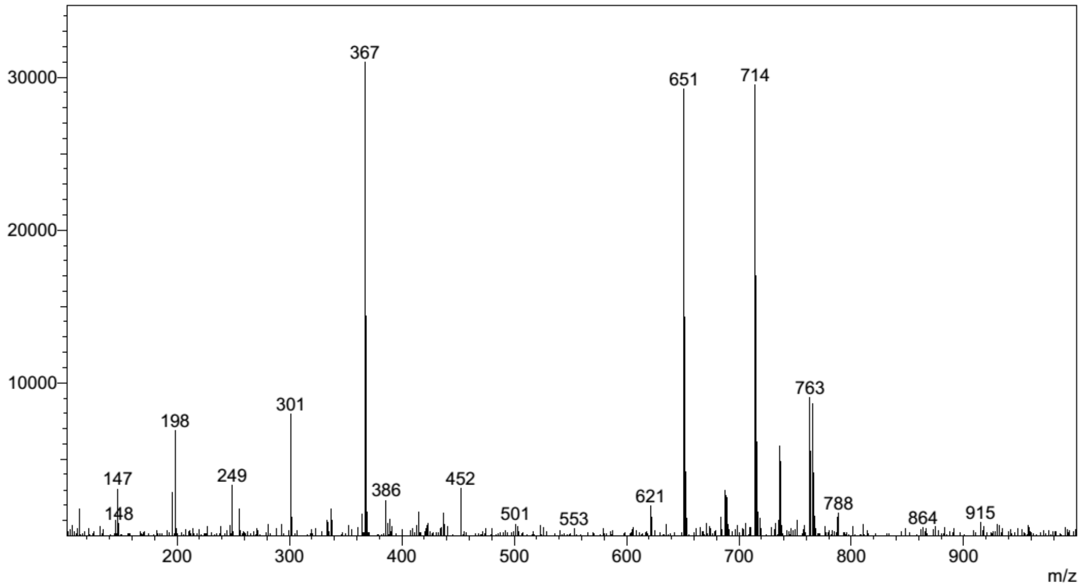


**Figure S77.** MS-ESI for compound **13**.

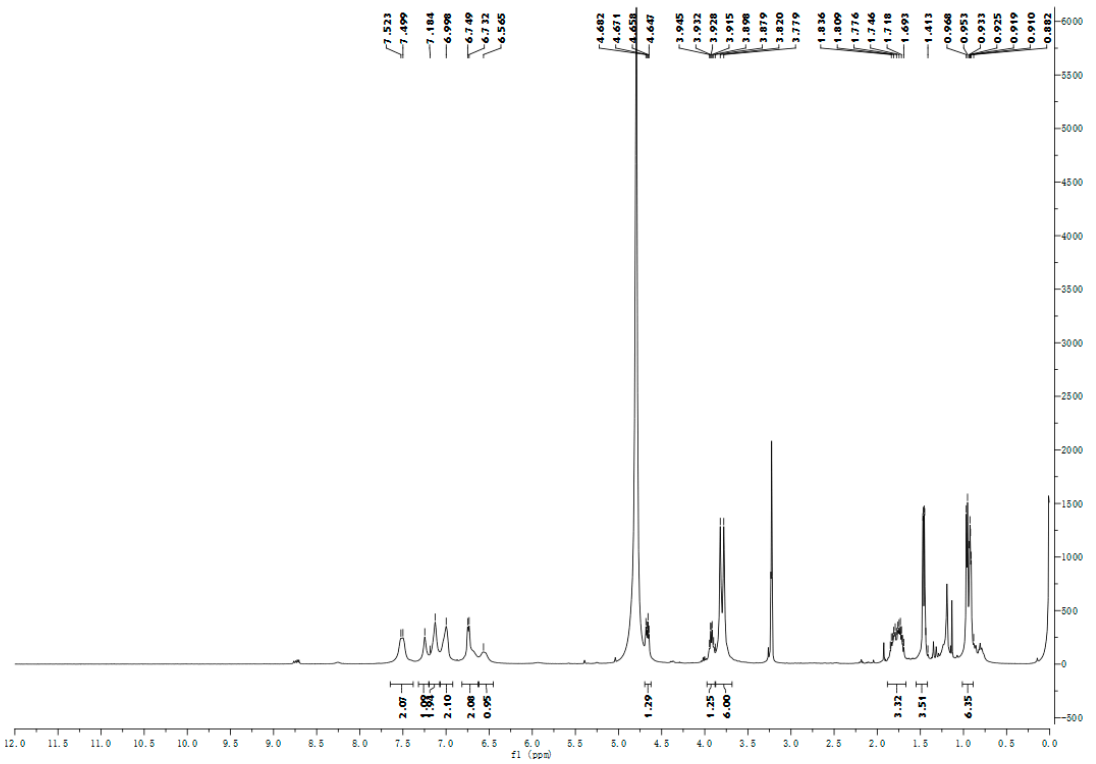

**Figure S78.** $^{1}$H-NMR for compound **14**.

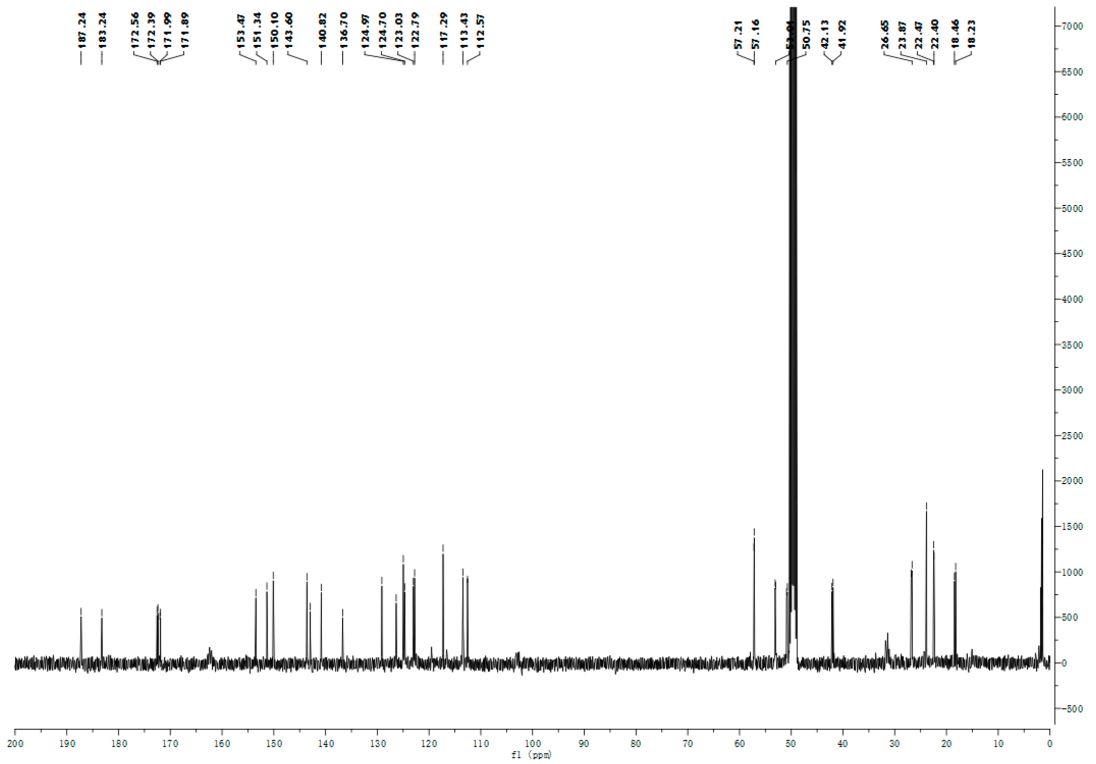

**Figure S79.** $^{13}$C-NMR for compound **14**.

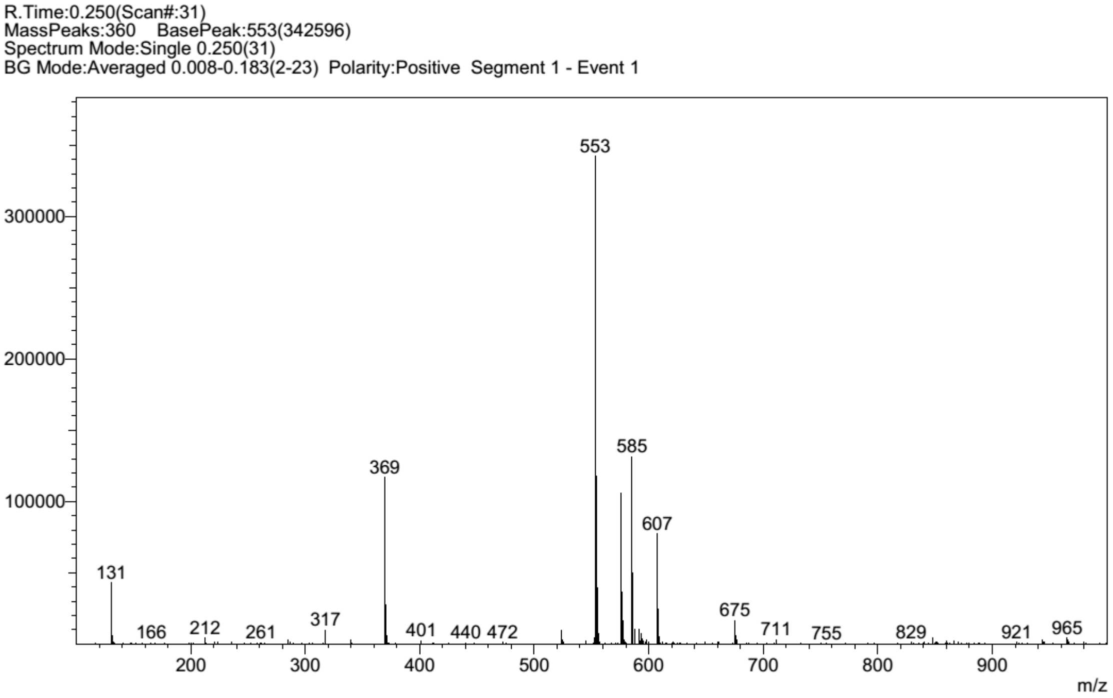


**Figure S80.** MS-ESI for compound **14**.

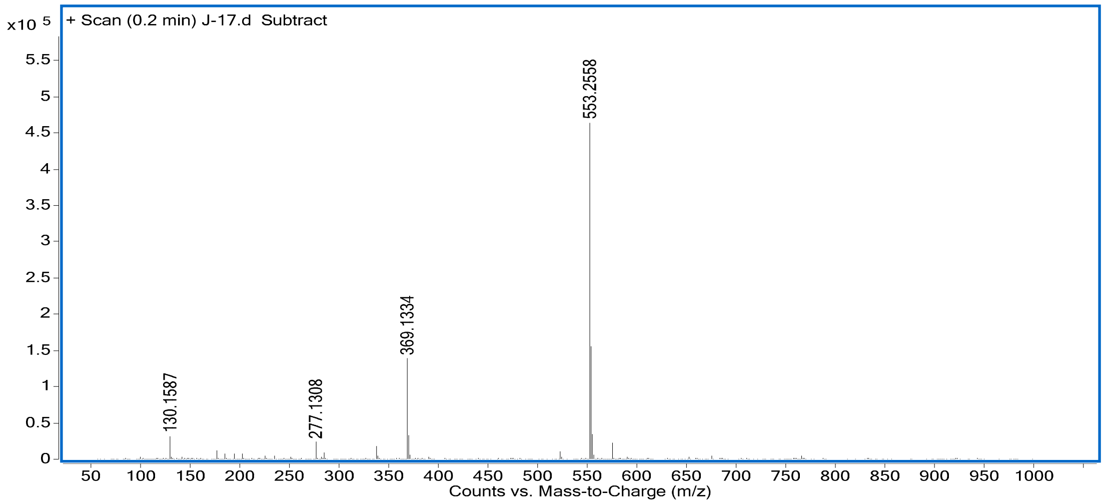


**Figure S81.** HRMS-ESI for compound **14**.

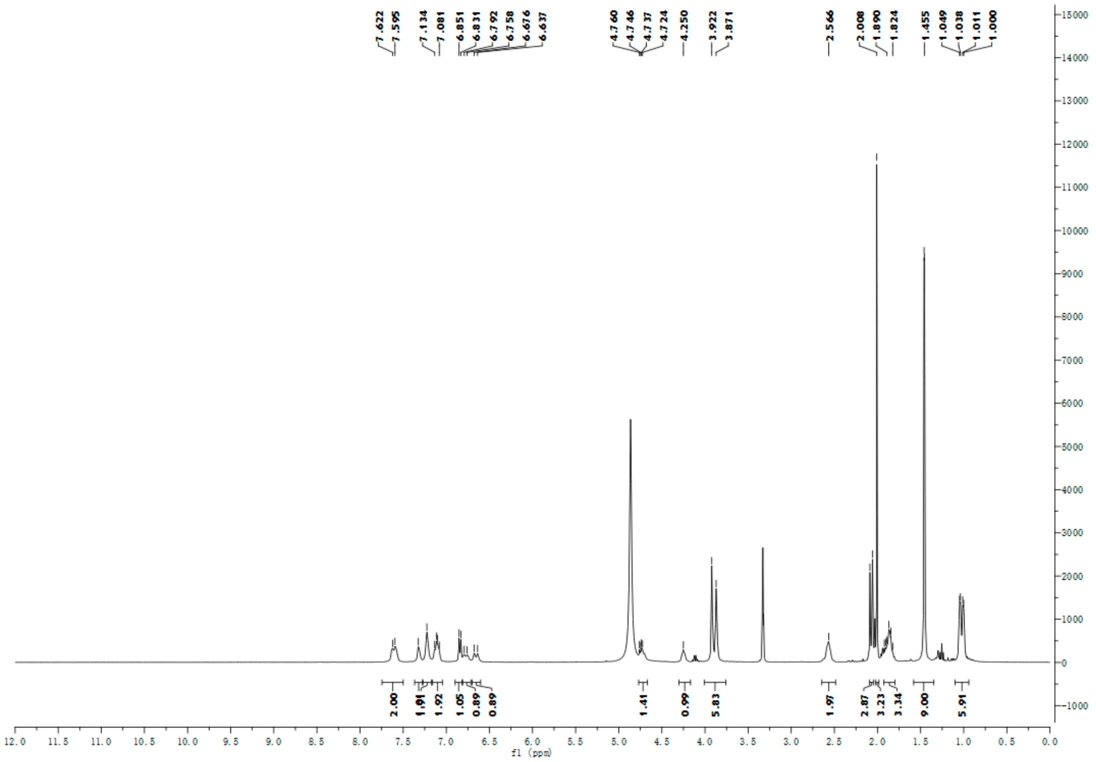

**Figure S82.** $^{1}$H-NMR for compound **15**.

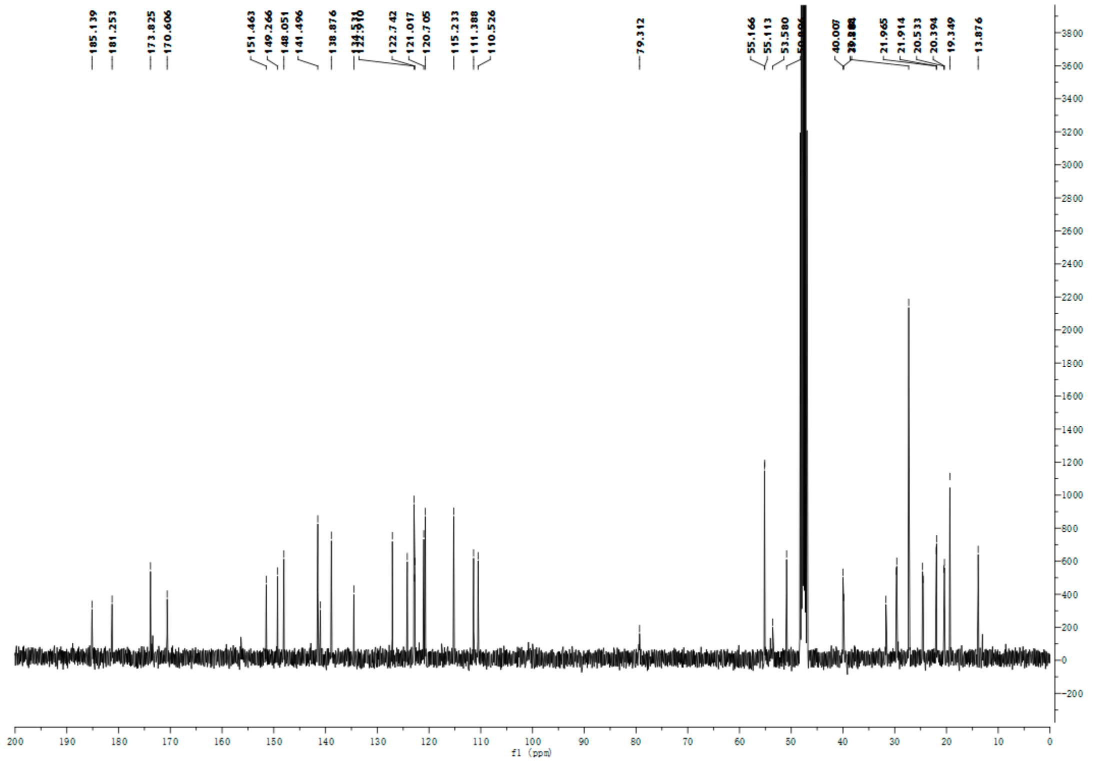

**Figure S83.** $^{13}$C-NMR for compound **15**.

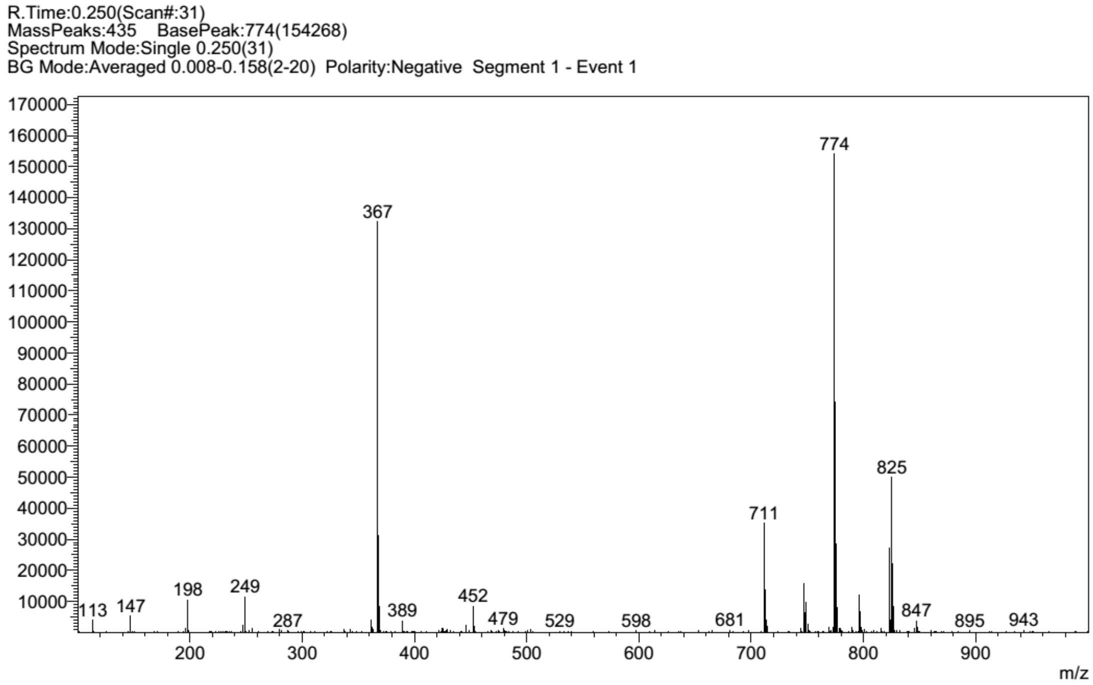


**Figure S84.** MS-ESI for compound **15**.

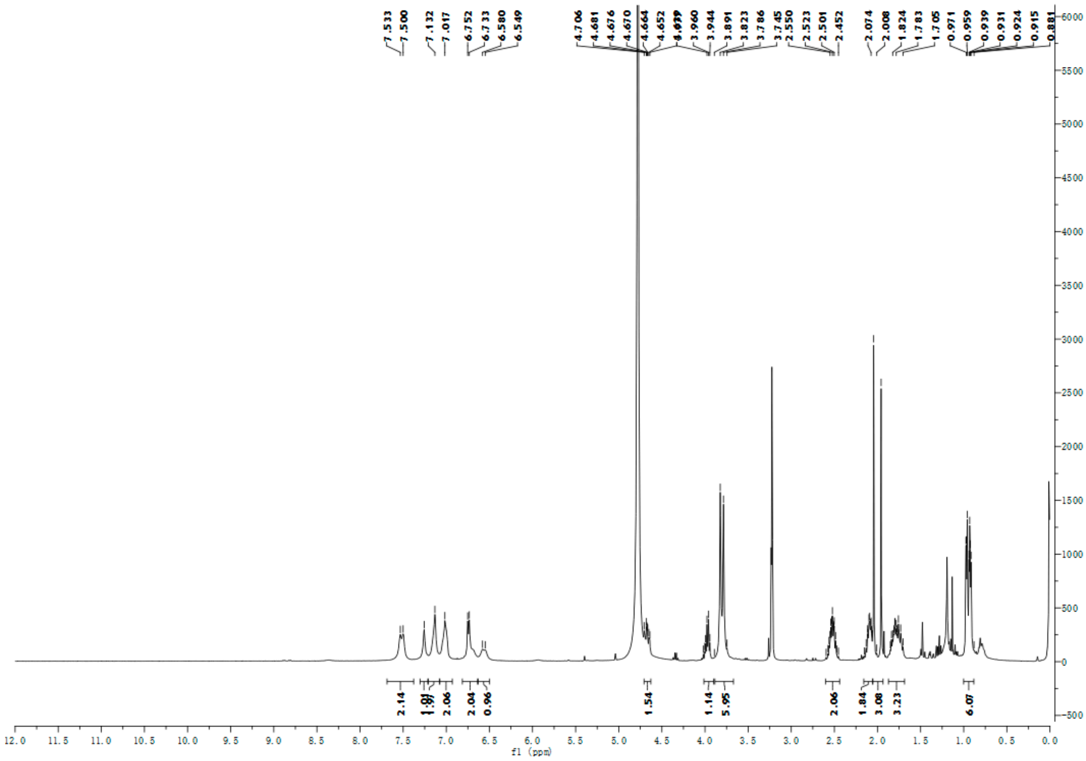


**Figure S85.** $^{1}$H-NMR for compound **16**.

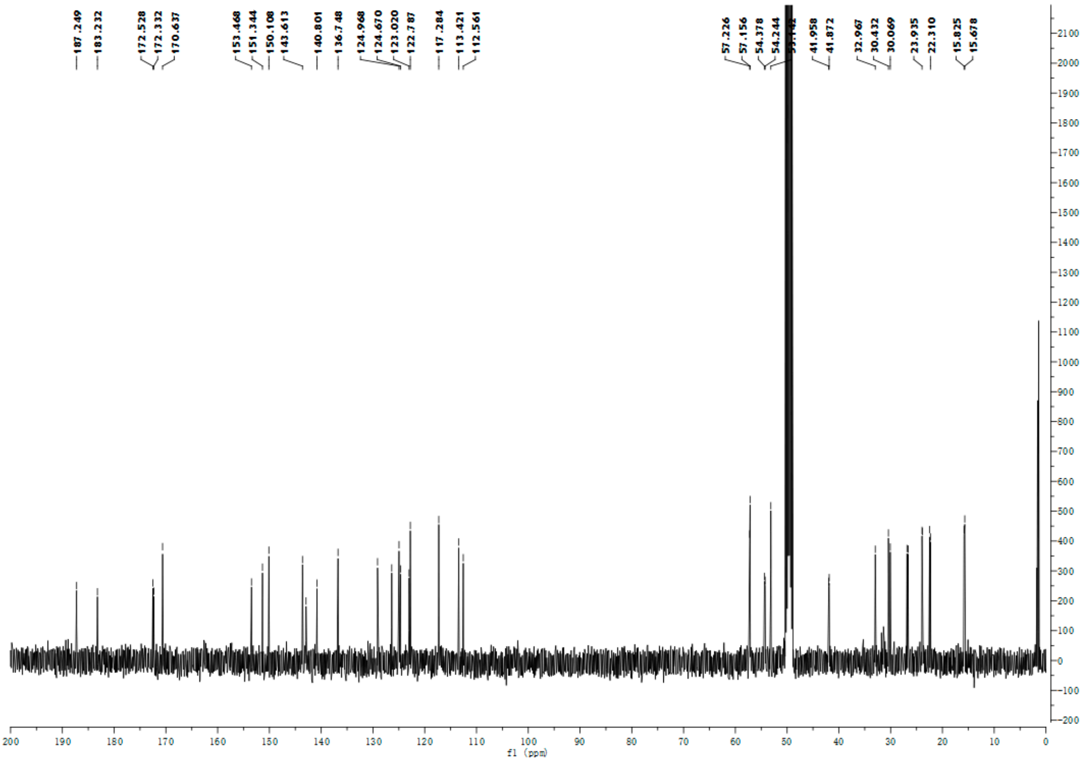


**Figure S86.** $^{13}$C-NMR for compound **16**.

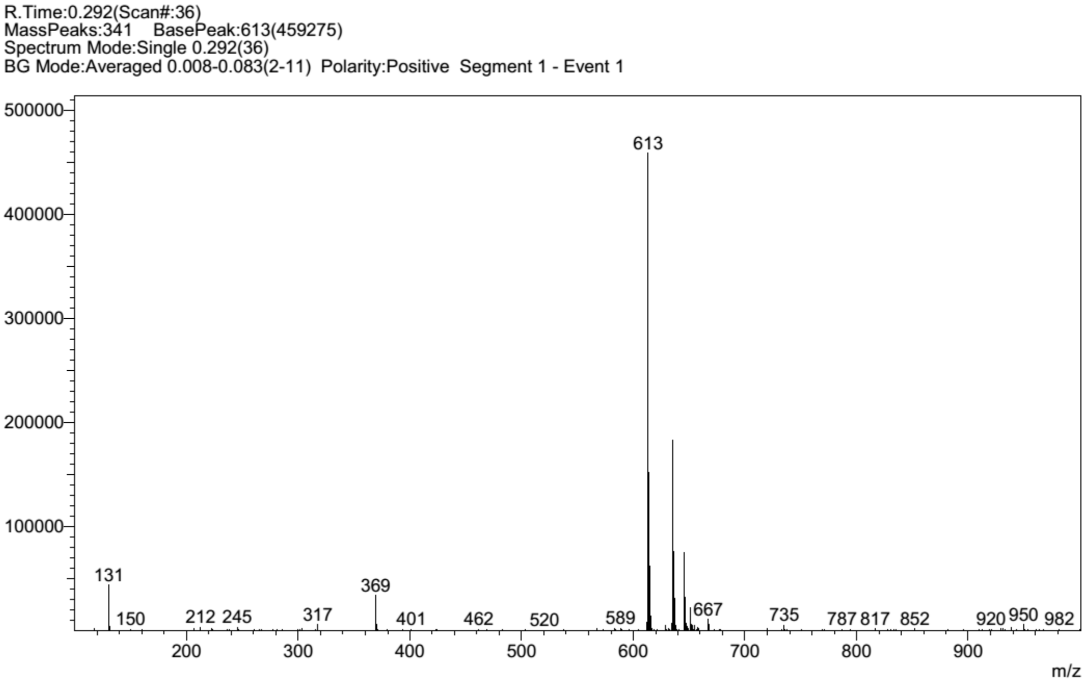


**Figure S87.** MS-ESI for compound **16**.

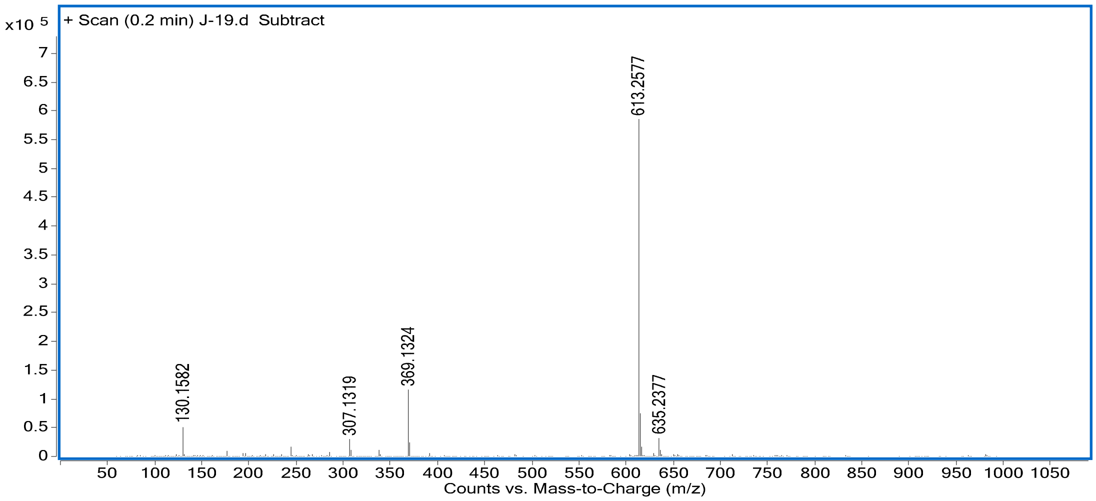


**Figure S88.** HRMS-ESI for compound **16**.

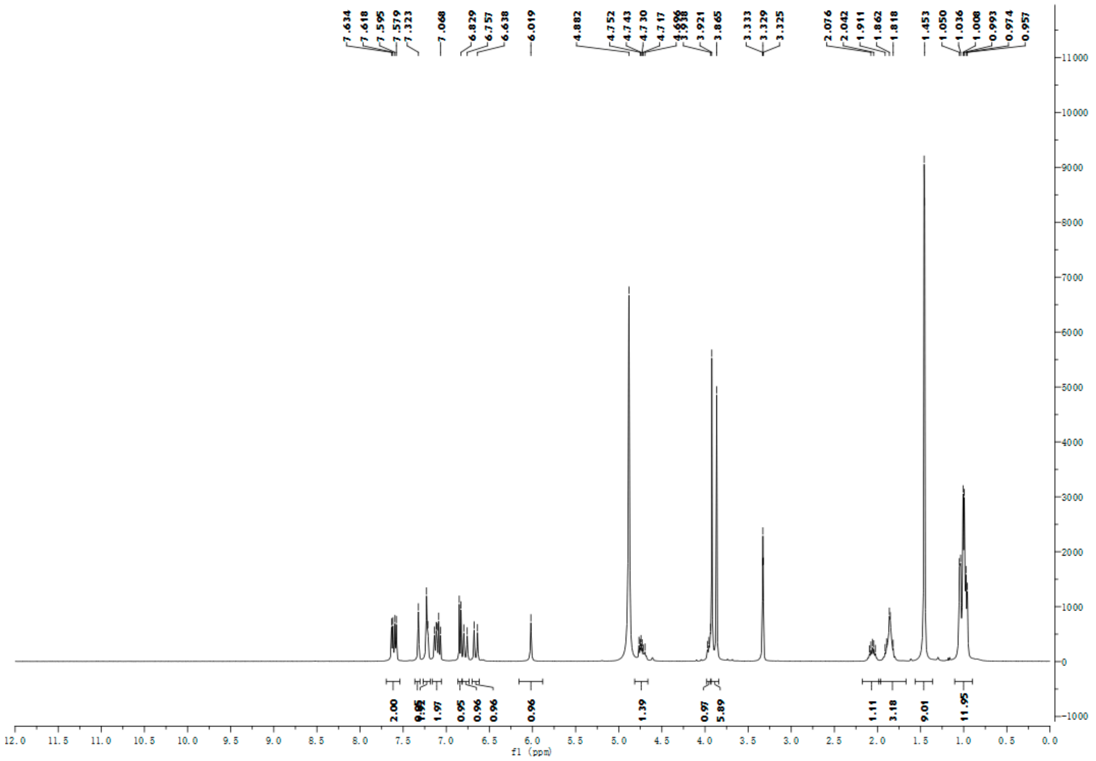


**Figure S89.** $^{1}$H-NMR for compound **17**.

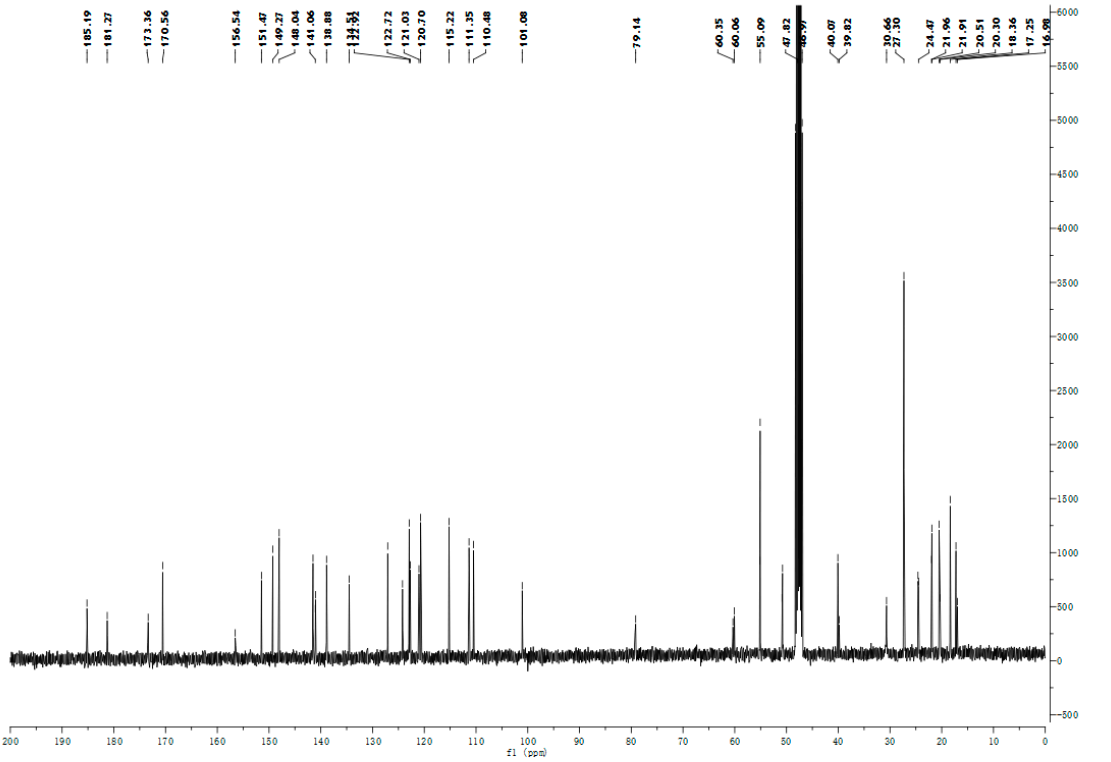


**Figure S90.** $^{13}$C-NMR for compound **17**.

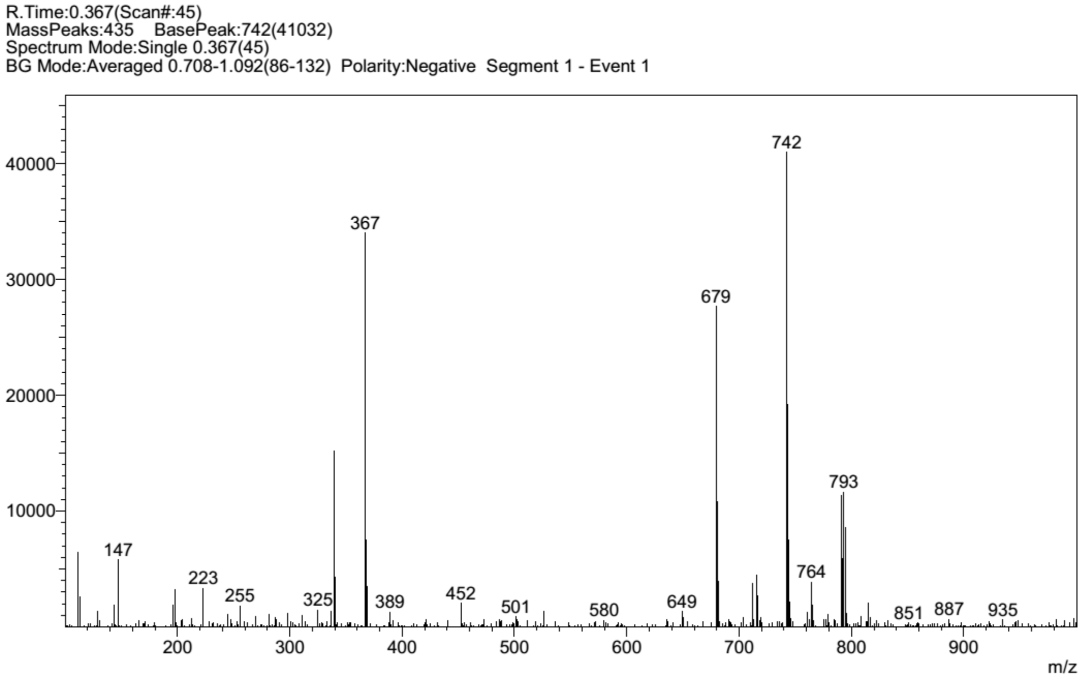


**Figure S91.** MS-ESI for compound **17**.

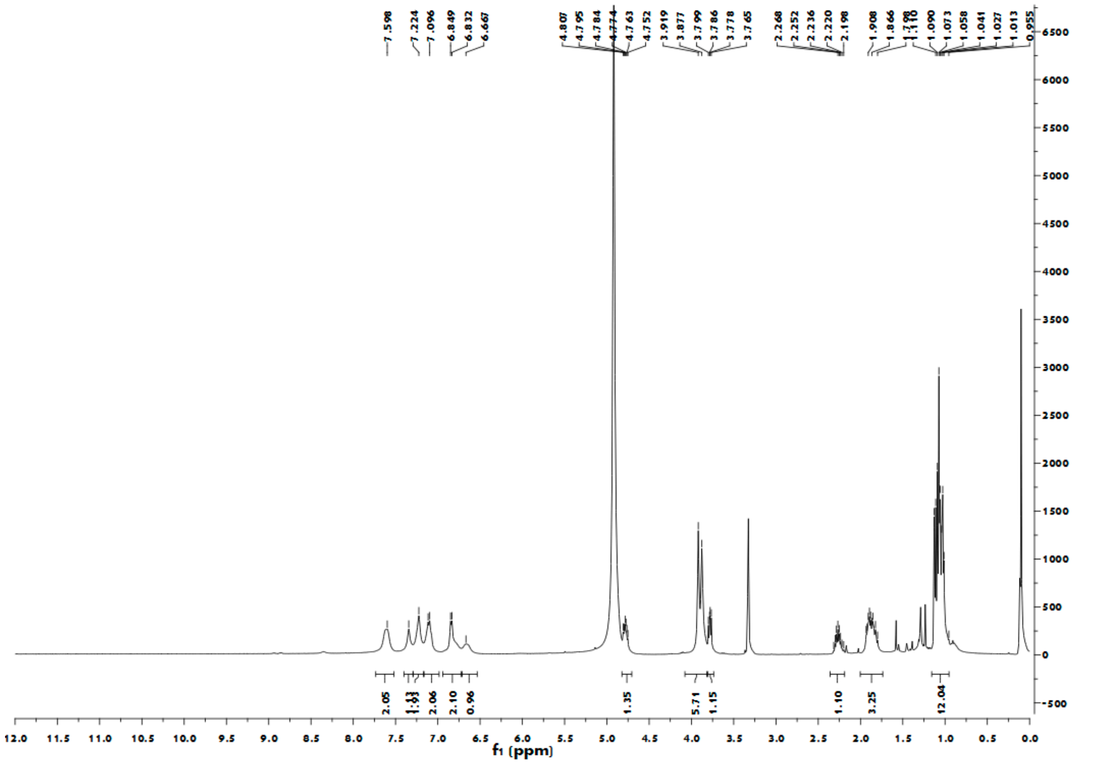

**Figure S92.** $^{1}$H-NMR for compound **18**.

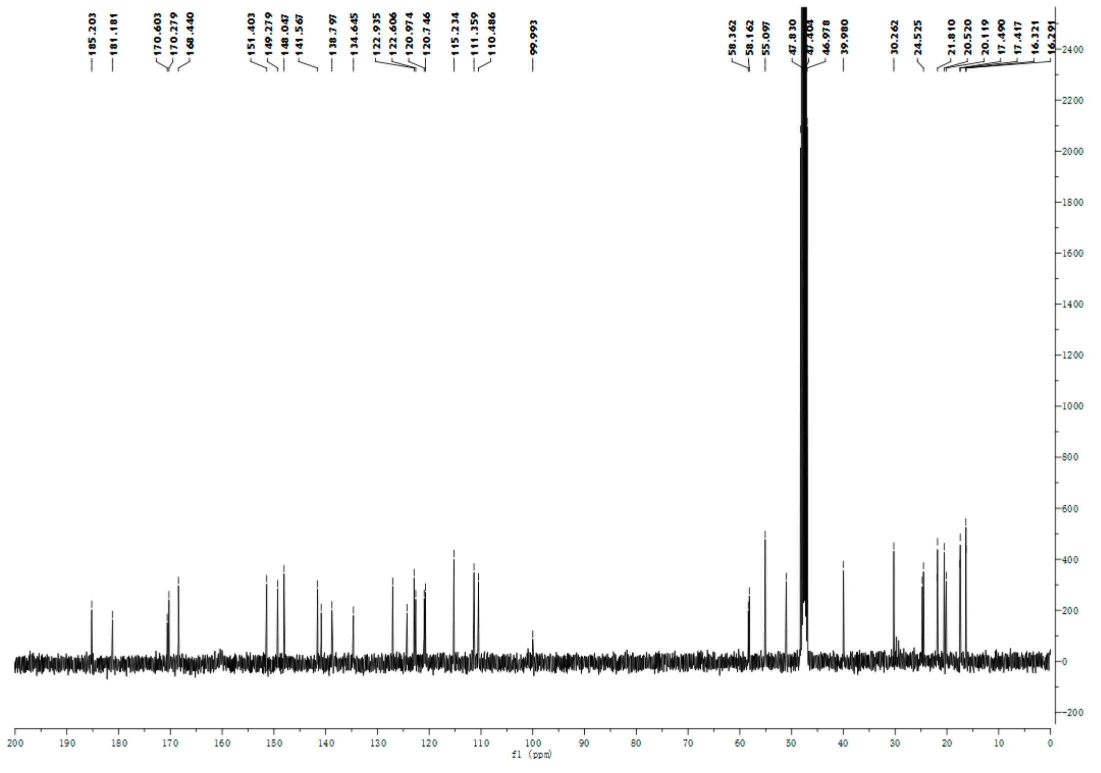

**Figure S93.** $^{13}$C-NMR for compound **18**.

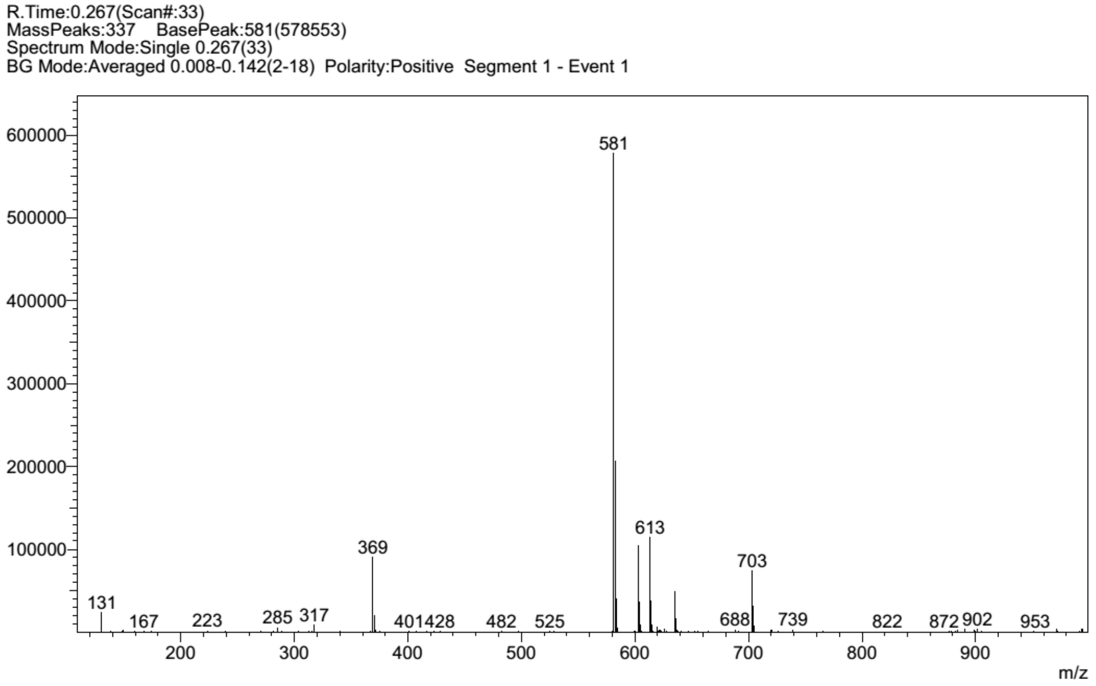


**Figure S94.** MS-ESI for compound **18**.

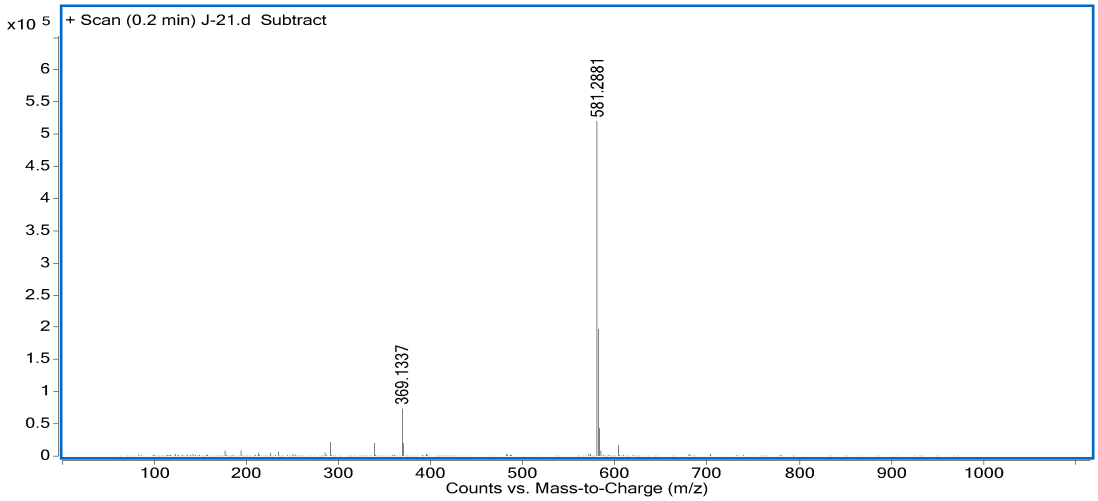


**Figure S95.** HRMS-ESI for compound **18**.
